# On resin synthesis of sulfated oligosaccharides†

**DOI:** 10.1039/d1sc06063e

**Published:** 2022-01-31

**Authors:** Theodore Tyrikos-Ergas, Eric T. Sletten, Jhih-Yi Huang, Peter H. Seeberger, Martina Delbianco

**Affiliations:** Department of Biomolecular Systems, Max-Planck Institute of Colloids and Interfaces Am Mühlenberg 1 14476 Potsdam Germany martina.delbianco@mpikg.mpg.de; Department of Chemistry and Biochemistry, Freie Universität Berlin Arnimallee 22 14195 Berlin Germany

## Abstract

Sulfated glycans are involved in many biological processes, making well-defined sulfated oligosaccharides highly sought molecular probes. These compounds are a considerable synthetic challenge, with each oligosaccharide target requiring specific synthetic protocols and extensive purifications steps. Here, we describe a general on resin approach that simplifies the synthesis of sulfated glycans. The oligosaccharide backbone, obtained by Automated Glycan Assembly (AGA), is subjected to regioselective sulfation and hydrolysis of protecting groups. The protocol is compatible with several monosaccharides and allows for multi-sulfation of linear and branched glycans. Seven diverse, biologically relevant sulfated glycans were prepared in good to excellent overall yield.

## Abstract 2

Well-defined sulfated oligosaccharides are important synthetic targets. We present an on resin approach for the synthesis of sulfated glycans with a broad reaction scope that overcomes previous limitations associated with on resin synthesis.

## Graphical abstract

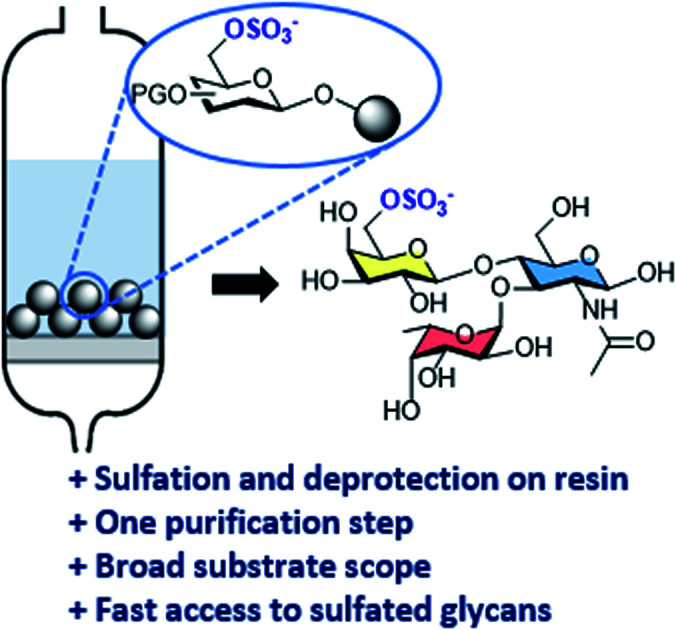

## Introduction

Sulfation of glycans is a significant post-glycosylation modification.^1^ The polyanionic nature of sulfated glycans results in strong affinity to positively charged surfaces on lectins, facilitating a multitude of interactions that mediate essential biological processes.^2^ The specific function of these sulfated compounds strongly depends on the degree of sulfation as well as on the sulfation pattern along the glycan chain.^3,4^ Protocols for the selective introduction of sulfate groups on complex oligosaccharide structures have been developed.^5^ Nevertheless, the synthesis of sulfated glycans remains exceedingly challenging. Selective sulfation mandates the rational placement of orthogonal protecting groups (PGs), thus adding extra complexity to the already challenging synthesis of the oligosaccharide backbone. Upon sulfation, the removal of the residual PGs can be troublesome and further synthetic manipulations must avoid migration and/or cleavage of the labile sulfate moieties.^6^

**Fig. 1:**
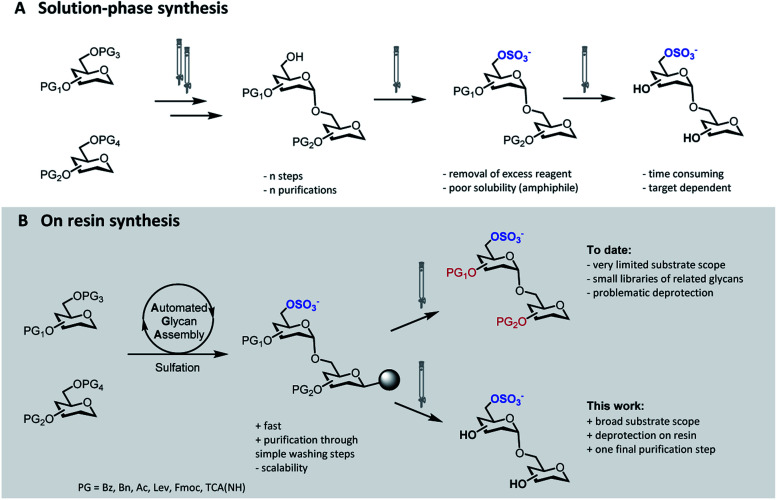
Synthetic approaches to access sulfated glycans: classical solution-phase synthesis (A), on resin synthesis (this work, B).

The identification of biologically active sulfation patterns requires versatile, adaptable, and rapid synthetic methodologies. Over the past decades, a variety of solution-phase methodologies has been developed, permitting the assembly of highly complex structures.^7–9^ Still, the production of these compounds is far from trivial, with each target often requiring further optimization.^10^ Tedious chromatographic purifications after each step (normal phase, reverse phase and/or size exclusion) are unavoidable, since the accumulation of unreacted reagents, side-products, and the presence of salts can affect the next reaction steps (Fig. 1A). Moreover, sulfation of multiple positions becomes progressively more difficult, due to steric hindrance and anion crowding commonly leading to incomplete reactions.^11^ The increase in product polarity impedes purifications due to the amphiphilic nature of the partially protected intermediates. Thus, the early stage introduction of sulfate diesters that can be deprotected in the final step of the synthesis has been explored.^5,12^

Solid-phase approaches (Fig. 1B) offer an alternative to traditional solution-phase synthesis,^13^ as exemplified by the preparation of a small collection of keratan sulfate (KS) tetrasaccharides.^14^ In this example, the backbone, prepared by Automated Glycan Assembly (AGA), was sulfated on resin, before solution-phase removal of all the PGs yielded the target compounds. Still, to date, solid-phase approaches were powerful to access neutral backbones,^15^ but have shown a limited substrate scope, poor reproducibility and, in most cases, proved unsuccessful in obtaining the final deprotected sulfated targets.^16,17^ Solid-phase approaches are currently less scalable than solution phase syntheses, making purification and deprotection of the “off resin” compound very challenging, often resulting in loss of compounds.

Here, we report a general strategy for the solid-phase synthesis of various sulfated oligosaccharides (Fig. 1B). The glycan backbone is constructed by AGA. Optimized sulfation and ester hydrolysis procedures yield the partially protected intermediate on solid support. Only simple washing steps are required for the removal of excess reagents. Cleavage from the solid support followed by hydrogenolysis of the remaining PGs allows for access to the desired sulfated glycan. Throughout the protocol, only a final purification step is required. Synthetic bottlenecks associated with particular structural features are identified and overcome. The general approach is showcased in the synthesis of seven diverse, biologically relevant targets.

## Results and discussion

**Fig. 2:**
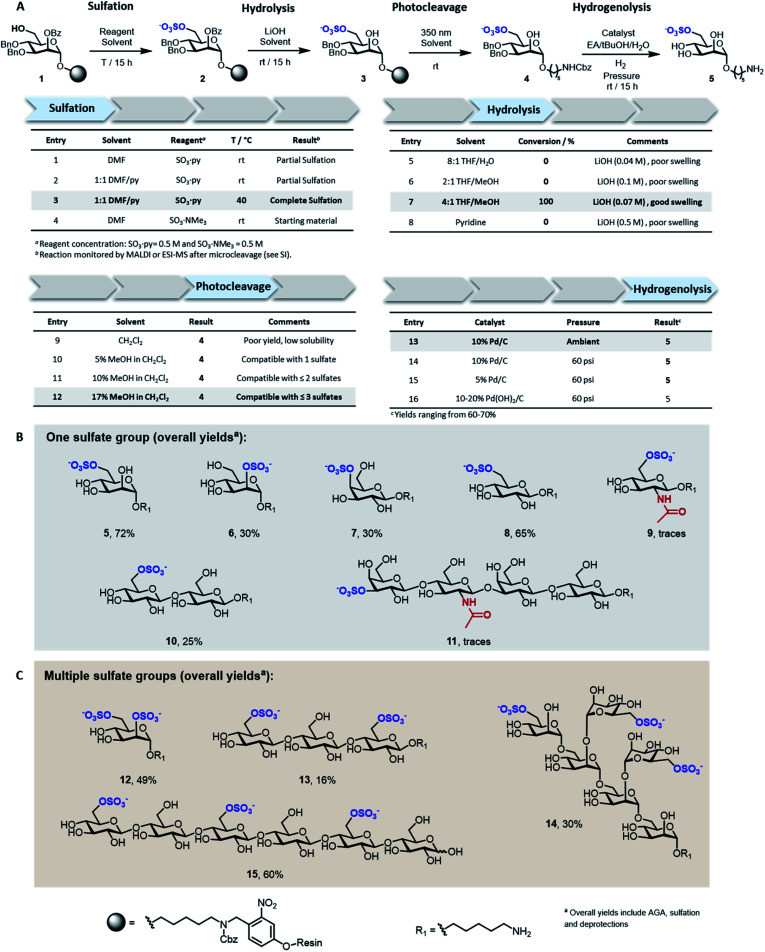
Discovery of solid-phase synthesis conditions for sulfated oligosaccharides exemplified for the resin-bound mannoside 1 (A). Collection of mono- (B) and multi-sulfated (C) compounds obtained with the optimized conditions highlighted in grey in panel A.

Solid-supported mannoside 1 bearing a free hydroxyl group at C-6 was prepared by AGA (see ESI†) and selected as model substrate for the development of the sulfation protocol (Fig. 2A). To obtain the sulfated target 5, four key steps (*i.e.* sulfation, hydrolysis, photocleavage, hydrogenolysis) had to be optimized to reach full conversion and avoid the accumulation of side-products.

In search for an efficient sulfating procedure compatible with the solid support, we screened different conditions based on sulfur trioxide amine complexes. Incomplete sulfation was observed using sulfur trioxide pyridine complex (SO_3_·py) at ambient temperature (Fig. 2A, entries 1 and 2). Compound 1 was readily sulfated when the reaction was conducted at 40 °C for 15 h in a DMF/Py mixture (Fig. 2A, entry 3). No sulfation occurred when SO_3_·NMe_3_ was employed (Fig. 2A, entry 4).

Lithium hydroxide (LiOH) is frequently utilized for ester hydrolysis of sulfated oligosaccharides.^18^ To translate this procedure to the solid-phase paradigm, we set to identify a solvent system that dissolves LiOH and swells the polystyrene resin. The compromise proved troublesome since LiOH is poorly soluble in apolar solvents, often employed to guarantee proper resin swelling. The low LiOH concentration accessible in an 8 : 1 THF/water mixture (0.04 M) together with poor swelling of the resin resulted in no conversion (Fig. 2A, entry 5). Replacing water with MeOH permitted to increase the LiOH concentration (0.07 M), while maintaining suitable resin swelling. Full conversion was obtained when hydrolysis was performed in a 4 : 1 THF/MeOH mixture (Fig. 2A, entry 7). In an effort to increase the concentration of LiOH further, pyridine was added, but despite higher concentrations (0.5 M), no product was observed (Fig. 2A, entry 8). The hydrophilic nature of compound 3 required the addition of MeOH to the photocleavage cocktail, generally performed^19^ in pure CH_2_Cl_2_ (Fig. 2A, entries 9–12).

The removal of the benzyl (Bn) groups was performed by catalytic heterogeneous hydrogenolysis. Different catalysts were tested, showing no significant differences in the reaction outcome (Fig. 2A, entries 13–16). The catalyst was removed by filtration and the product purified by a reverse phase C18 and LH-20 size exclusion chromatography. The target compound was obtained as sodium salt upon treatment with Na^+^ exchange resin for a 72% overall yield (over five steps including AGA, sulfation and deprotections).

To assess the generality of the protocol, six monosaccharides and five oligosaccharides based on different monosaccharide units, PGs, and glycosidic linkages were prepared (Fig. 2B and C). All glycan backbones were assembled following standard AGA protocols (see ESI† for complete protocols). The optimized sulfation procedure worked smoothly for all primary alcohols, regardless of the glycan length. Full conversion was also observed for secondary hydroxyl groups, including the poorly reactive hydroxyl groups at the C-2 position of mannose 6 and the C-4 position of galactose 7 (Fig. 2B). Multiple sulfate groups were introduced on a glycan (13–15) as well as on the same monosaccharide (12) proving ion crowding was not a challenge. Complex structures, including the trisulfated branched hexamannoside (14) and cellohexasaccharide (15), were successfully sulfated without any adjustments (Fig. 2C). Hydrolysis of the ester PGs was completed within 15 h for the majority of the sulfated targets. Longer reaction times were required for the sulfated cellulose oligomers 13 (24 h) and 15 (48 h) and lacto-*N*-tetraose (LNT) 11 (120 h), as previously reported for non-sulfated analogues.^20^ The mild conditions were compatible with long debenzoylation reactions as no side-products were observed. Reaction progress was easily monitored by ESI-mass spectrometry analysis on a minute reaction sample after microcleavage (Fig. S9†). Removal of Lev or Fmoc PGs could be achieved during the hydrolysis step or selectively using hydrazine or triethylamine, respectively (Fig. S11†).

Adjustments to the photocleavage solvent system were required for structures containing multiple sulfate groups to guarantee good solubility of the cleaved compound while maintaining ample resin swelling (Fig. 2A, entries 10–12). The final hydrogenolysis of the Bn ethers proceeded smoothly under standard conditions (Fig. 2B, entry 13). With this protocol, linear and branched oligosaccharides (5–10 and 12–15) were obtained in good to excellent overall yields. The entire protocol, including AGA, sulfation, and deprotections required less than 72 h, even for complex structures like 14 and 15. Simple access to collections of complex sulfated mannans (14), highly abundant in seaweeds, will fuel the characterization of marine glycans and their processing enzymes.^21^ Moreover, pattern-controlled sulfated cellulose analogues like 15 are interesting targets to mimic the bioactivity of heparin,^22,23^ in which the anticoagulant activity is influenced by the position of sulfates. As well, sulfated cellulose structures are important compounds in materials science.^24^

**Fig. 3:**
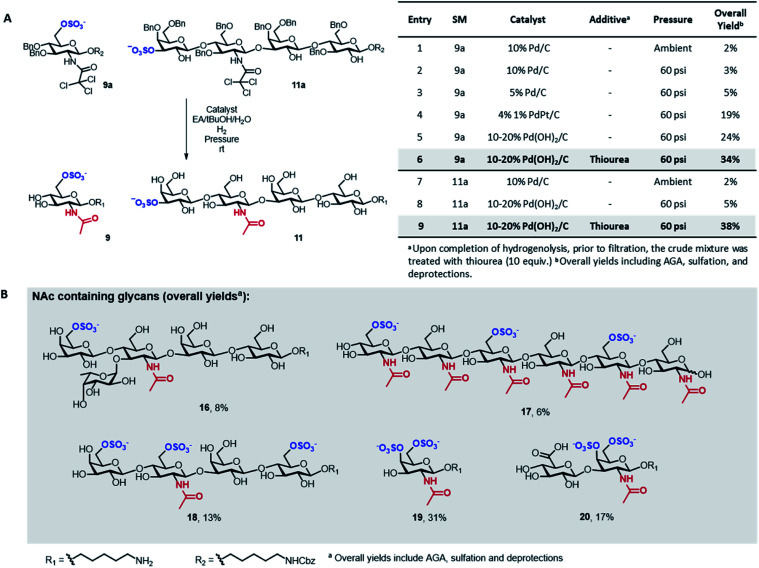
Screening of conditions for the hydrogenolysis of the Bn ethers in 9a and 11a (A). Biologically relevant GlcNAc and GalNAc containing glycans obtained with the optimized protocol highlighted in grey in panel A (B).

A major limitation of the approach was revealed when attempting to synthesize compounds containing an acetylamino-2-deoxy-glucoside (GlcNAc) in their backbone (9 and 11). In these instances, the desired products were only obtained in trace amounts. The problem seemed associated with the final hydrogenolysis step, as mass spectrometry indicated full conversions for all prior transformations and a significant amount of crude product was obtained after photocleavage (Fig. S8 and S9†). Only trace amounts of product were recovered upon hydrogenolysis of 9a using 10% Pd/C (Fig. 3A, entries 1 and 2) even though MS-QTOF indicated complete removal of the Bn PGs (Fig. S10 and S19†).

Since sulfated *N*-acetyl glucosamine residues are an important part of biologically active glycans,^25^ we set out to overcome this limitation by screening different catalysts and conditions using compound 9a as model system (Fig. 3A). The use of a more disperse Pd/C catalyst^26^ can improve hydrogenolysis yields; however, in our case, the use of 5% Pd/C was not beneficial (Fig. 3A, entry 3). A remarkable increase in yield was observed using PdPt/C or Pd(OH)_2_/C as catalysts in a pressurized reactor (Fig. 3A, entries 4 and 5). However, these catalysts failed when the reaction was performed with 11a as starting material (Fig. 3A, entry 8). Loss of sulfated compounds during hydrogenolysis is a known issue; similar observations have been reported during the synthesis of hyaluronans^27^ and in the synthesis of a 24-mer of chondroitin sulfate, where the yield drastically dropped during hydrogenolysis.^7^ The significant drop in yields are often tolerated because solution phase synthesis are commonly performed on a larger scale, but becomes much more dramatic when dealing with the smaller quantities compatible with solid-phase approaches, often resulting in complete loss of compound.^17^

We hypothesized that the low yields were the result of the strong coordination of the target compounds to the palladium catalyst. We envisioned the treatment of the reaction mixture, upon completion of hydrogenolysis, with a palladium scavenger to promote ligand exchange and release the desired product. Much higher yields were obtained when the crude mixture was treated with thiourea, a molecule with high affinity to palladium (Fig. 3A, entries 6 and 9). The overall yield of sulfated 11 significantly improved from 5 to 38% (Fig. 3A, entry 9). Size exclusion chromatography assured the complete removal of thiourea from the target compound (Fig. S20†).

Next, we explored the scope of the new protocol for the synthesis of biologically relevant compounds bearing the acetylamino moiety (Fig. 3B). Two sulfated LNT structures (11 and 18) as well as the 6-*O*-SO_3_^−^ Lewis^*x*^ antigen 16 were prepared to study their interaction with the human galectin-4 ^28^ and L-selectins,^29^ respectively. The synthesis of the trisulfated chitin oligomer 17 yielded a heparin-like anticoagulant structure with a defined degree and pattern of substitution, crucial to avoid toxicity.^30^

During the synthesis of tetrasaccharide 18, treatment with lithium hydroxide unexpectedly caused the cleavage of the TCA PGs. The modular methodology allowed for fast, selective acetylation of the free amino group directly on resin, prior to photocleavage, using 15% acetic anhydride in DMF. The loss of the TCA group was also observed for compounds 19 and 20, where the subsequent re-acetylation was not fully selective such that some hydroxyl groups were acetylated. Advantageously, the unwanted *O*-acetyls were chemoselectively removed by repeating the on resin LiOH hydrolysis step, facilitating the purification and ultimately yielding the final compounds in good overall yields. The successful synthesis of compounds 19 and 20 indicate that the protocol offers a promising approach for the assembly of well-defined glycosaminoglycan targets.

## Conclusions

In conclusion, we have addressed several issues related to the synthesis of sulfated glycans on solid support. We identified SO_3_·py as a generally effective reagent for on resin sulfation. We identified resin swelling related issues in the solid phase ester hydrolysis and overcame them by careful selection of reagents and solvent mixtures. We addressed solubility issues encountered with partially protected sulfated glycans during photocleavage. Moreover, we identified metal coordination as the most likely detriment to the high yielding debenzylation of acetylamino-containing sulfated glycans. The resolution of these problems resulted in a general on resin approach for the synthesis of sulfated glycans with a broad reaction scope. A diverse collection of sulfated glycans, including multi-sulfated linear and branched hexasaccharides was prepared. The robust approach will fuel the production of collections of sulfated glycans that are needed for systematic biological studies exploiting microarray technologies.^31^

## Data availability

All experimental and characterisation data are available in the ESI.†

## Author contributions

M. D. conceived this project. T. T. E. developed the protocols and synthesised all the target compounds. E. T. S. assisted with the protocol development and optimized the analytical characterization. J. Y. H. assisted with the synthesis of the BBs. M. D. supervised the project. T. T. E., E. T. S., J. Y. H., P. H. S., and M. D. contributed to and discussed the manuscript.

## Conflicts of interest

There are no conflicts to declare.

## Supplementary Material

SC-013-D1SC06063E-s001
