# Supplementary material for: On resin synthesis of sulfated oligosaccharides
Source: Chem Sci. 2022 Jan 31;13(7):2115–20. doi: 10.1039/d1sc06063e (PMC8848854; doi:10.1039/d1sc06063e)
Supplement: SC-013-D1SC06063E-s001 [file SC-013-D1SC06063E-s001.pdf]

Electronic Supplementary Material (ESI) for Chemical Science.
This journal is © The Royal Society of Chemistry 2022

Supporting Information

Table of Contents

1 General materials and methods ... 3

2 Building blocks for AGA ... 4

3 Synthesis of BB8 ... 5

3.1 Ethyl 4,6-*O*-benzylidene-3-*O*-benzyl-1-thio-β-glucopyranoside, S2 ... 5

3.2 Ethyl 4,6-*O*-benzylidene-3-*O*-benzyl-2-*O*-benzoyl-1-thio-β-glucopyranoside, S3 ... 6

3.3 Ethyl 3-*O*-benzyl-2-*O*-benzoyl-1-thio-β-glucopyranoside, S4 ... 7

3.4 Ethyl 3-*O*-benzyl-2-*O*-benzoyl-6-*O*-levulinoyl-1-thio-β-glucopyranoside, S5 ... 9

3.5 Ethyl 3-*O*-benzyl-2-*O*-benzoyl-6-*O*-levulinoyl-4-*O*-(9-fluorenylmethoxycarbonyl)-1-thio-β-glucopyranoside, BB8 ... 12

4 Synthesis of BB15b ... 15

4.1 Dibutoxyphosphoryloxy 3-*O*-benzyl-4-*O*-(9-fluorenylmethoxycarbonyl)-2-deoxy-2-*N*-trichloroacetyl-6-*O*-levulinoyl-α-glucopyranoside, BB15b ... 15

5 Synthesis of BB16 ... 18

5.1 Dibutoxyphosphoryloxy 3-*O*-benzyl-4-*O*-benzyl-6-*O*-(9-fluorenylmethoxycarbonyl) -2-deoxy-2-*N*-trichloroacetyl-α -glucopyranoside, BB16 ... 18

6 Synthesis of BB17 ... 21

6.1 Phenyl 2-deoxy-2-*N*-trichloroacetylamino-3-O-levulinoyl-1-thio-β-galactopyranoside, S7 ... 21

6.2 Phenyl 4,6-di-*O*-(9-fluorenylmethoxycarbonyl)-2-deoxy-2-*N*-trichloroacetylamino-3-*O*-levulinoyl-1-thio-β-galactopyranoside, S8 ... 24

6.3 Dibutoxyphosphoryloxy 4,6-di-*O*-(9-fluorenylmethoxycarbonyl)-2-deoxy-2-*N*-trichloroacetylamino-3-*O*-levulinoyl-α/β-galactopyranoside, BB17 ... 27

7 Synthesis of BB18b ... 30

7.1 Methyl (Dibutoxyphosphoryloxy 4-O-levulinoyl-3-O-benzyl-2-O-benzoyl-β-glucopyranosyluronate), BB20 ... 30

8 Automated Glycan Assembly ... 33

8.1 General materials and method ... 33

8.2 Preparation of stock solutions ... 33

8.3 Modules for automated synthesis ... 34

8.4 Post-synthesizer manipulations (Post-AGA) ... 38

9 Glycan backbones obtained by AGA ... 40

10 Oligosaccharides synthesis ... 41

10.1 Synthesis of 5 41
10.2 Synthesis of 6 44
10.3 Synthesis of 7 47
10.4 Synthesis of 8 50
10.5 Synthesis of 9 53
10.6 Synthesis of 10 56
10.7 Synthesis of 11 59
10.8 Synthesis of 12 62
10.9 Synthesis of 13 66
10.10 Synthesis of 14 70
10.11 Synthesis of 15 74
10.12 Synthesis of 16 78
10.13 Synthesis of 17 82
10.14 Synthesis of 18 86
10.15 Synthesis of 19 90
10.16 Synthesis of 20 93
11 Mass spectrometry and additional information 96
11.1 Mass spectrometry analysis of the intermediate steps for the synthesis of 5 96
11.2 Mass spectrometry analysis of the intermediate steps for the synthesis of 14 99
11.3 Mass spectrometry analysis of the intermediate steps for the synthesis of 11 102
11.4 Mass spectrometry analysis of the intermediate steps for the synthesis of 24 and 27 104
11.5 Analysis of the hydrogenolysis reaction of compound 9a 109
12 References 111

1 General materials and methods

All chemicals used were reagent grade and used as supplied unless otherwise noted. The automated syntheses were performed on a home-built synthesizer developed at the Max Planck Institute of Colloids and Interfaces. Analytical thin-layer chromatography (TLC) was performed on Merck silica gel 60 F254 plates (0.25 mm). Compounds were visualized by UV irradiation or dipping the plate in a staining solution (sugar stain: 10% H_2SO_4 in EtOH; CAM: 48 g/L ammonium molybdate, 60 g/L ceric ammonium molybdate in 6% H_2SO_4 aqueous solution). Flash column chromatography was carried out by using forced flow of the indicated solvent on Fluka Kieselgel 60 M (0.04 – 0.063 mm). Analysis and purification by normal and reverse phase HPLC were performed using the Agilent 1260 series equipped with a Multiple Wavelength Detector (MWD) and an Evaporative Light Scattering Detector (ELSD). Products were lyophilized using a Christ Alpha 2-4 LD plus freeze dryer. ^{1}H, ^{13}C and HSQC NMR spectra were recorded on a Varian 400-MR (400 MHz), a Varian 600-MR (600 MHz) or a Varian 700-MR (700 MHz) spectrometer. Spectra were recorded in $CDCl_3$ by using the solvent residual peak chemical shift as the internal standard ($CDCl_3$: 7.26 ppm ^{1}H, 77.0 ppm ^{13}C) or in D_2O using the solvent as the internal standard in ^{1}H NMR (D_2O: 4.79 ppm ^{1}H). The ^{1}H NMR were acquired without heteroatom decoupling. The ^{13}C and ^{31}P NMR were acquired with hydrogen atom decoupling. High resolution mass spectra were obtained using a 6210 ESI-TOF mass spectrometer (Agilent) and a MALDI-TOF autoflex™ (Bruker).

2 Building blocks for AGA

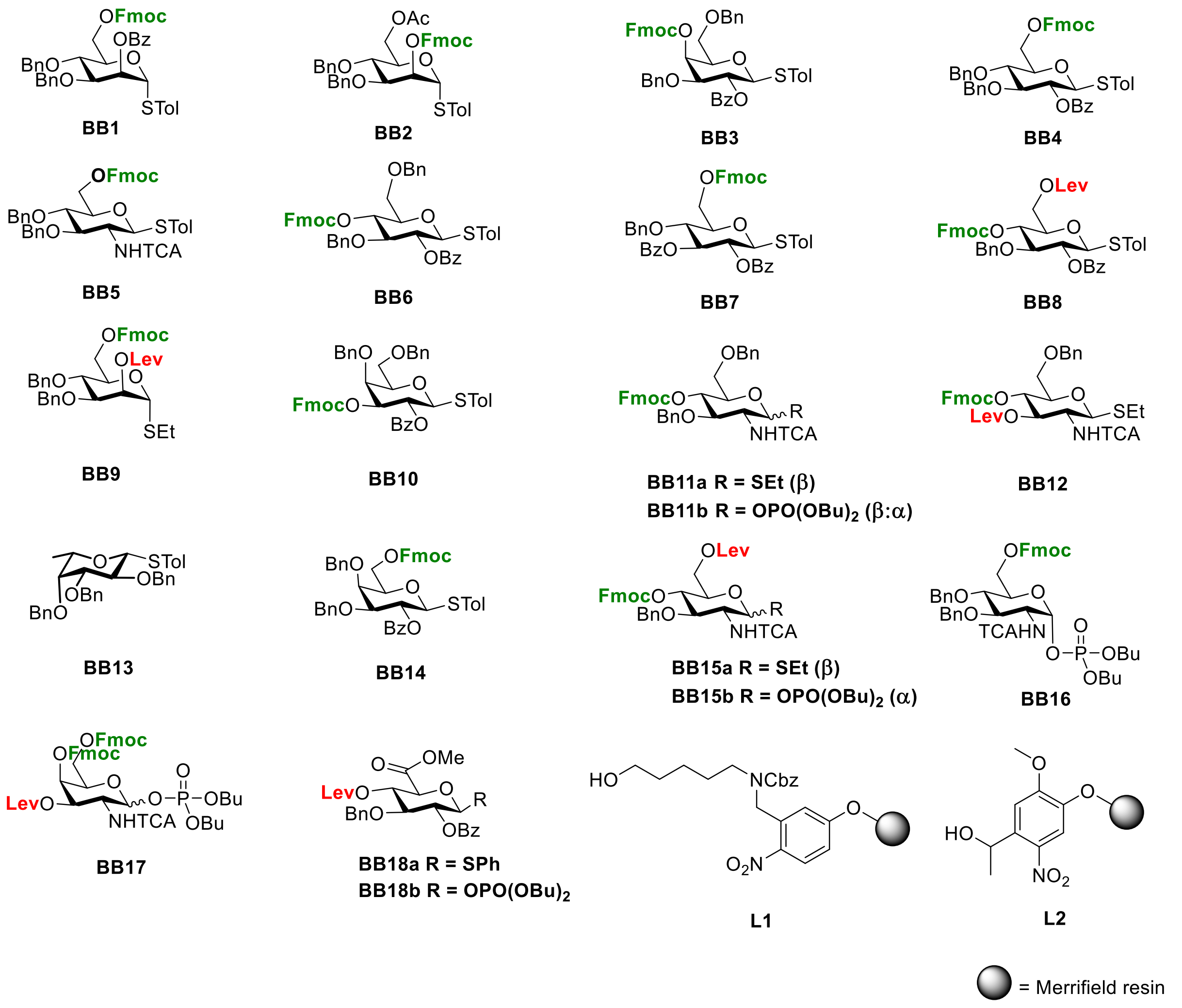

All BBs were purchased from GlycoUniverse apart from **BB8, BB11b, BB15b, BB16, BB17, BB18b.** Their synthesis is reported below. Merrifield resin equipped photocleavable linkers (**L1**, loading 0.30 mmol/g and **L2**, loading 0.34 mmol/g) were prepared according to previous literature.[1]

3 Synthesis of BB8

3.1 Ethyl 4,6-*O*-benzylidene-3-*O*-benzyl-1-thio-β-glucopyranoside, S2

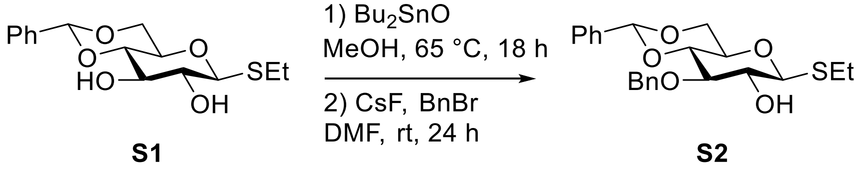

Compound **S1** (10.0 g, 32.0 mmol) was dissolved in MeOH (250 mL), di-*n*-butyltin oxide (9.6 g, 38.4 mmol) was added and the reaction mixture heated to 65 °C for 18 h. The reaction mixture was then cooled to rt, concentrated *in vacuo* and the residue dissolved in DMF (200 mL). Benzyl bromide (6.6 g, 38.4 mmol) and cesium(I) fluoride (6.32 g, 41.6 mmol) were added and the mixture stirred at rt for 24 h. The reaction mixture was concentrated *in vacuo* and the residue dissolved in CH_2Cl_2 (250 mL). The organic layer was washed with 1M potassium fluoride (100 mL, aq.), dried over ($MgSO_4$), filtered and concentrated *in vacuo*. The resulting crude was purified by column chromatography (Hexanes : EtOAc = 2:1) to give **S2** as a white solid (9.0 g, 22.3 mmol, 67%). ^{1}H NMR (400 MHz, $CDCl_3$) δ 7.51 – 7.47 (m, 2H), 7.41 – 7.30 (m, 8H), 5.58 (s, 1H), 4.98 (d, *J* = 11.6 Hz, 1H), 4.82 (d, *J* = 11.6 Hz, 1H), 4.47 (d, *J* = 9.7 Hz, 1H), 4.36 (dd, *J* = 10.5, 5.0 Hz, 1H), 3.83 – 3.64 (m, 3H), 3.62 – 3.44 (m, 2H), 2.82 – 2.68 (m, 2H), 1.32 (t, *J* = 7.4 Hz, 3H). NMR data were in agreement with previously reported.[2]

^{1}H NMR of S2 (400 MHz, $CDCl_3$)

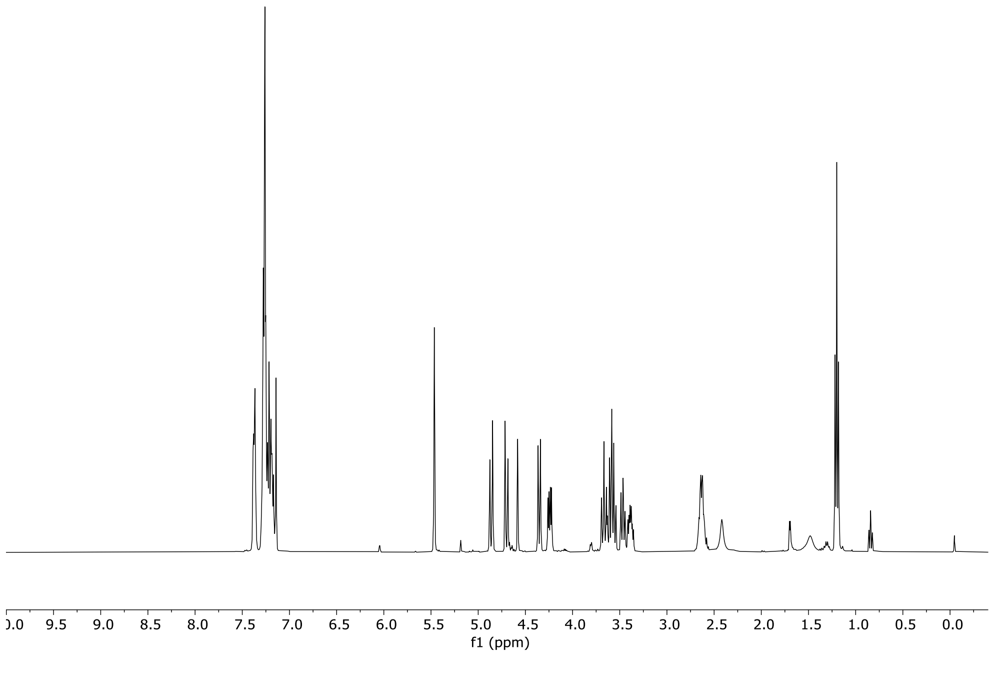

3.2 Ethyl 4,6-*O*-benzylidene-3-*O*-benzyl-2-*O*-benzoyl-1-thio-β-glucopyranoside, S3

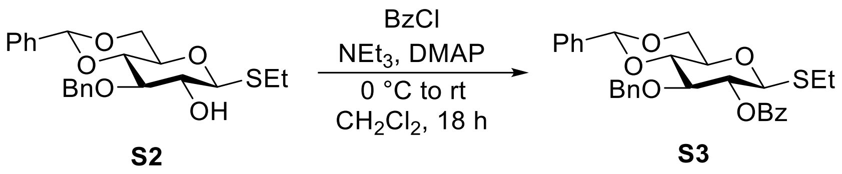

Compound **S2** (9.0 g, 22.3 mmol) was dissolved in anhydrous CH_2Cl_2 (200 mL). Triethylamine (8.7 mL, 67 mmol) and DMAP (825 mg, 6.7 mmol) were added slowly to the solution while stirring. Benzoyl chloride (3.9 mL, 33.5 mmol) was slowly added at 0 °C and the reaction allowed to rt. Upon completion (18 h) the reaction was quenched with sat. aq. solution of $NaHCO_3$. The mixture was washed three times with sat. aq. solution of $NaHCO_3$ and one time with brine. The organic layer was dried over $MgSO_4$ and concentrated under reduced pressure. The crude product was purified with flash chromatography (Hexanes : EtOAc = 6:1) to obtain **S3** as a white solid (10.5 g, 20.3 mmol, 92%). ^{1}H NMR (400 MHz, $CDCl_3$) δ 8.04 (dt, *J* = 8.4, 1.2 Hz, 2H), 7.64 – 7.38 (m, 9H), 7.20 – 7.08 (m, 5H), 5.64 (s, 1H), 5.37 (m, 1H), 4.90 – 4.70 (m, 2H), 4.65 (d, *J* = 10.1 Hz, 1H), 4.43 (dd, *J* = 10.5, 4.9 Hz, 1H), 3.98 – 3.80 (m, 3H), 3.58 (td, *J* = 9.5, 5.0 Hz, 1H), 2.74 (m, 2H), 1.24 (t, *J* = 7.4 Hz, 3H). NMR data were in agreement with previously reported.[3]

^{1}H NMR of S3 (400 MHz, $CDCl_3$)

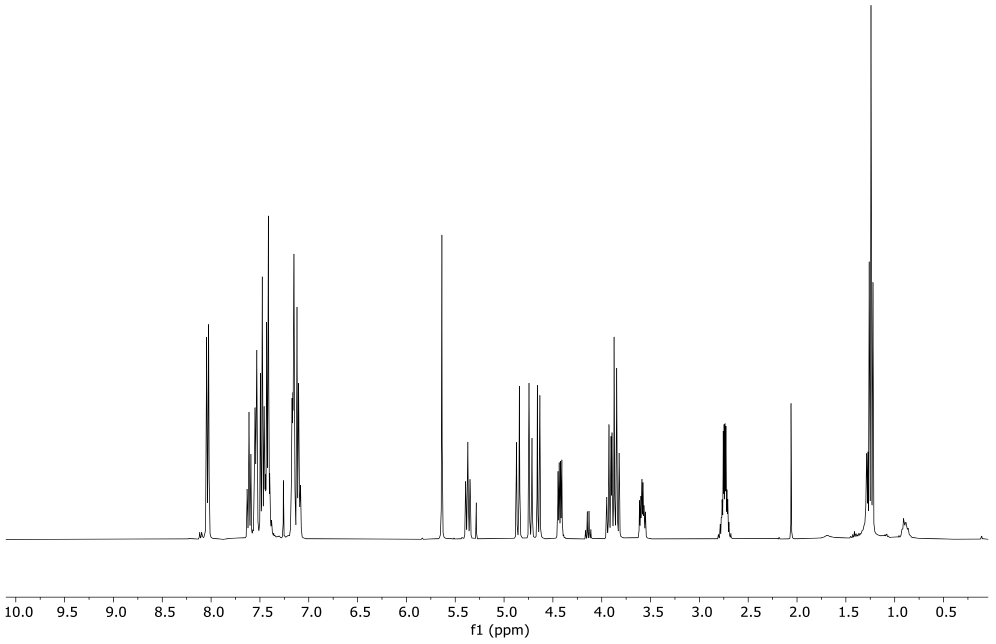

3.3 Ethyl 3-*O*-benzyl-2-*O*-benzoyl-1-thio-β-glucopyranoside, S4

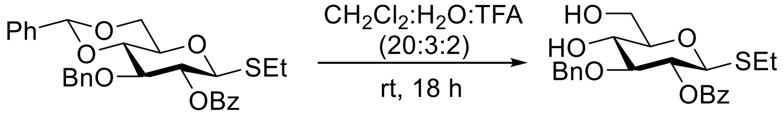

S3 **S4**

TFA (17 mL) and water (18 mL) were added to a solution of compound **S3** (10.5 g, 20.7 mmol) in CH_2Cl_2 (180 mL) and the mixture was stirred at rt for 18 h. The reaction was quenched with sat. aq. $NaHCO_3$ and extracted three times with CH_2Cl_2. The organic layer was dried over $MgSO_4$, filtered and concentrated under reduced pressure. The crude product was purified by flash chromatography (Hexanes : EtOAc = 1:1) to give compound **S4** as a white solid (7.0 g, 16.7 mmol, 80%). ^{1}H NMR (400 MHz, $CDCl_3$) δ 8.06 (dd, *J* = 8.0, 1.5 Hz, 2H), 7.60 (td, *J* = 7.3, 1.5 Hz, 1H), 7.47 (t, *J* = 7.8 Hz, 2H), 7.22 (m, 5H), 5.34 – 5.23 (m, 1H), 4.75 (d, *J* = 11.4 Hz, 1H), 4.66 – 4.54 (m, 2H), 3.98 – 3.67 (m, 4H), 3.48 (m, 1H), 2.71 (m, 2H), 2.20 (s, 2H), 1.23 (t, *J* = 7.4 Hz, 3H); ^{13}C NMR (101 MHz, $CDCl_3$) δ 165.4, 137.8, 133.5, 130.0, 129.8, 128.7, 128.7, 128.7, 128.2, 128.2, 128.2, 84.0, 84.0, 79.6, 74.9, 72.4, 70.6, 62.8, 24.3, 15.0; $[\alpha]_D^{20}$ = +87.09; IR (neat) ν_{max}= 2988, 1739, 1373, 1236, 1044; R_f = 0.15 (SiO_2, Hexanes : EtOAc = 1:1); HRMS (QToF): Calcd for $C_{22}H_{26}O_6SNa$ $[M+Na]^+$ 441.1362; found 441.1391.

^{1}H NMR of S4 (400 MHz, $CDCl_3$)

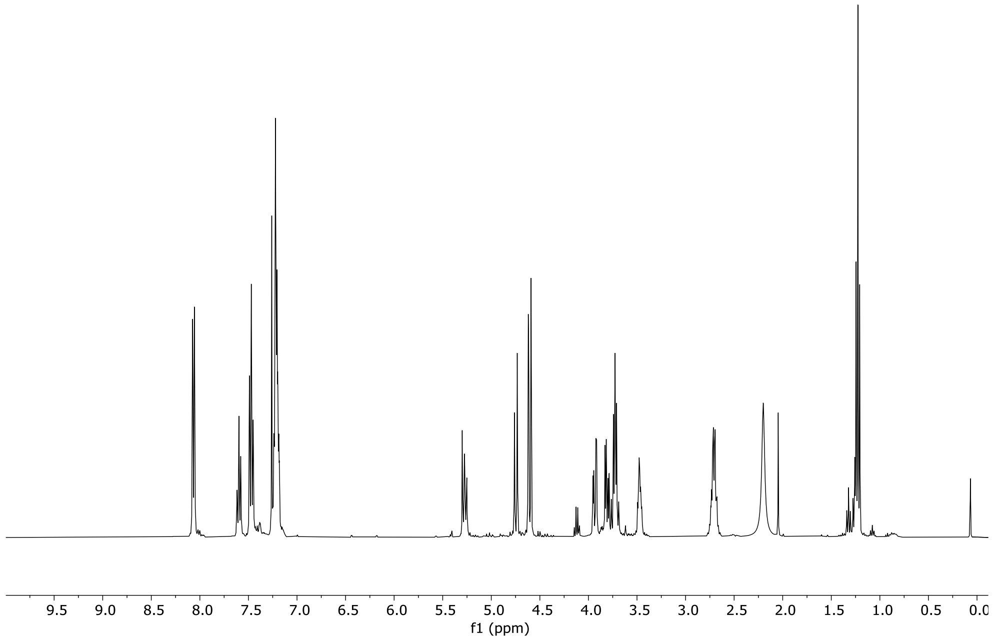

^{13}C NMR of S4 (101 MHz, $CDCl_3$)

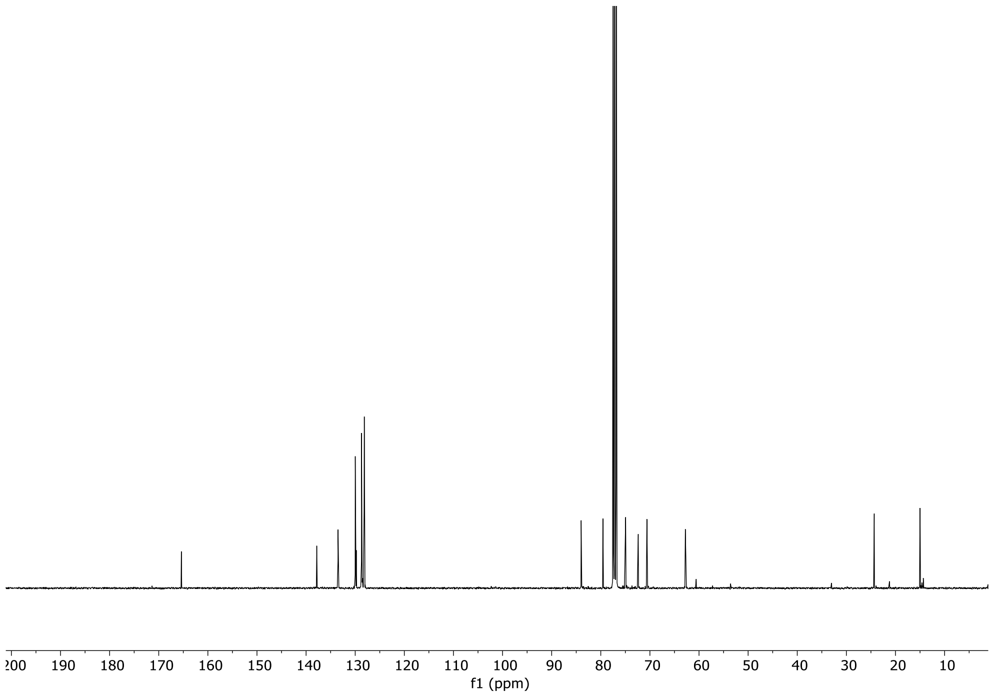

HSQC NMR of S4 ($CDCl_3$)

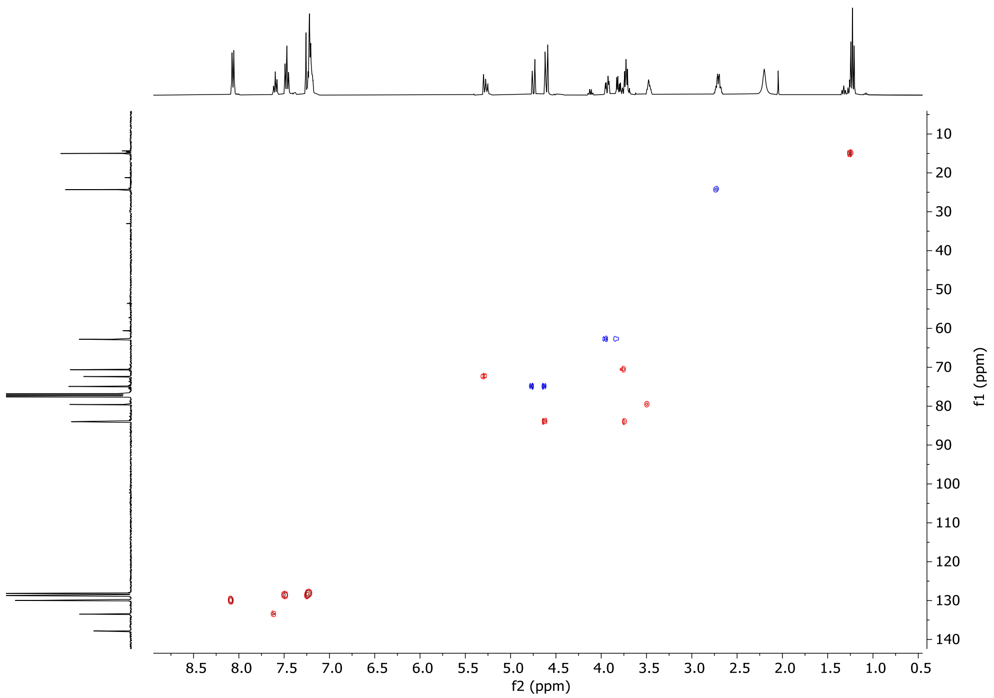

3.4 Ethyl 3-*O*-benzyl-2-*O*-benzoyl-6-*O*-levulinoyl-1-thio-β-glucopyranoside, S5

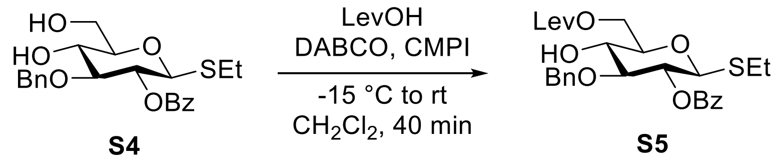

Compound **S4** (7.0 g, 16.75 mmol) was dissolved in CH_2Cl_2 (200 mL). Levulinic acid (3.45 mL, 33.5 mmol) and 2-chloro-1-methylpyridinium iodide (8.5 gr, 33.5 mmol) were added. The reaction was stirred for 15 min, then cooled to -15 °C and DABCO (7.5 g, 67 mmol) was added. The reaction mixture was stirred for 40 min and then filtered over a plug of celite and concentrated *in vacuo*. The reaction mixture was quenched with sat. aq. $NaHCO_3$ (200 mL) and extracted with CH_2Cl_2. The organic layer was washed with brine and dried over Na_2SO_4. Solvent removed by reduced pressure and purification by flash chromatography (Hexanes : EtOAc = 1:1) afforded compound **S5** as a white solid (7.3 g, 14.1 mmol, 84%). ^{1}H NMR (400 MHz, $CDCl_3$) δ 8.11 (dt, *J* = 8.1, 1.1 Hz, 2H), 7.70 – 7.63 (m, 1H), 7.57 – 7.49 (m, 2H), 7.26 (s, 5H), 5.39 – 5.29 (m, 1H), 4.78 (q, *J* = 11.4 Hz, 2H), 4.68 – 4.52 (m, 2H), 4.39 (dd, *J* = 12.2, 2.1 Hz, 1H), 3.82 – 3.70 (m, 2H), 2.98 – 2.66 (m, 7H), 2.27 (s, 3H), 1.41 – 1.25 (m, 3H); ^{13}C NMR (101 MHz, $CDCl_3$) δ 207.0, 173.5, 165.4, 137.9, 133.4, 130.0, 129.9, 128.6, 128.2, 128.0, 84.0, 83.4, 78.1, 75.0, 72.2, 70.2, 63.5, 38.14, 30.0, 28.0, 24.3, 15.0; $[\alpha]_D^{20}$ = + 35.51; IR (neat) v_{max}= 2930, 1721, 1361, 1273.8, 1070,749, 713; R_f = 0.5 (SiO_2, Hexanes : EtOAc = 1:1); HRMS (QToF): Calcd for $C_{27}H_{32}O_6SNa$ $[M+Na]^+$ 539.1716; found 539.1716.

^{1}H NMR of S5 (400 MHz, $CDCl_3$)

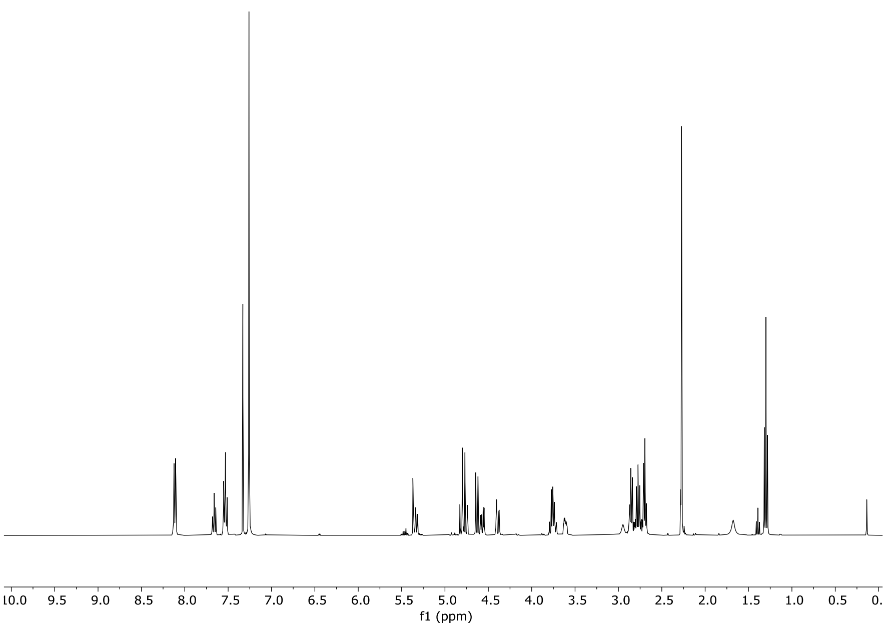

^{13}C NMR of S5 (101 MHz, $CDCl_3$)

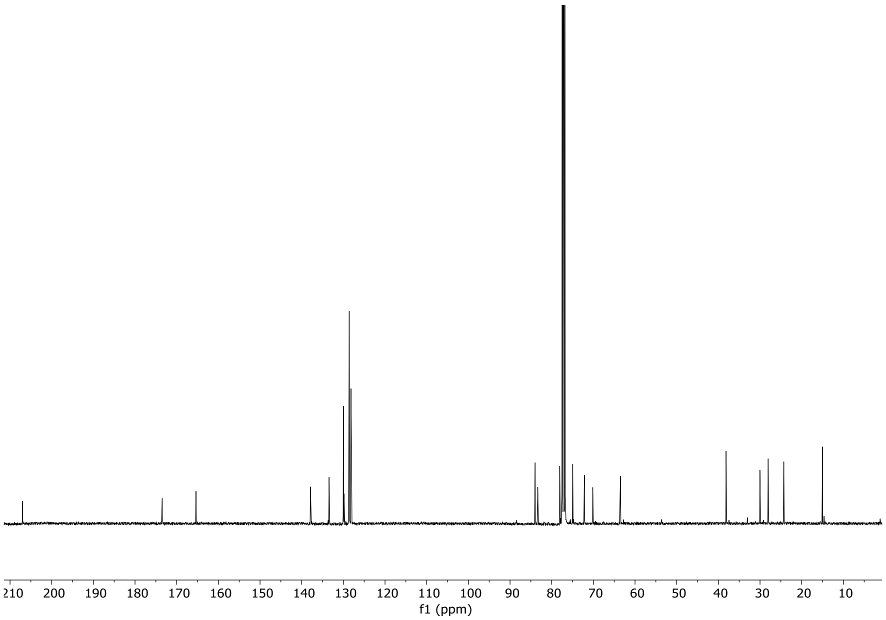

HSQC NMR of S5 ($CDCl_3$)

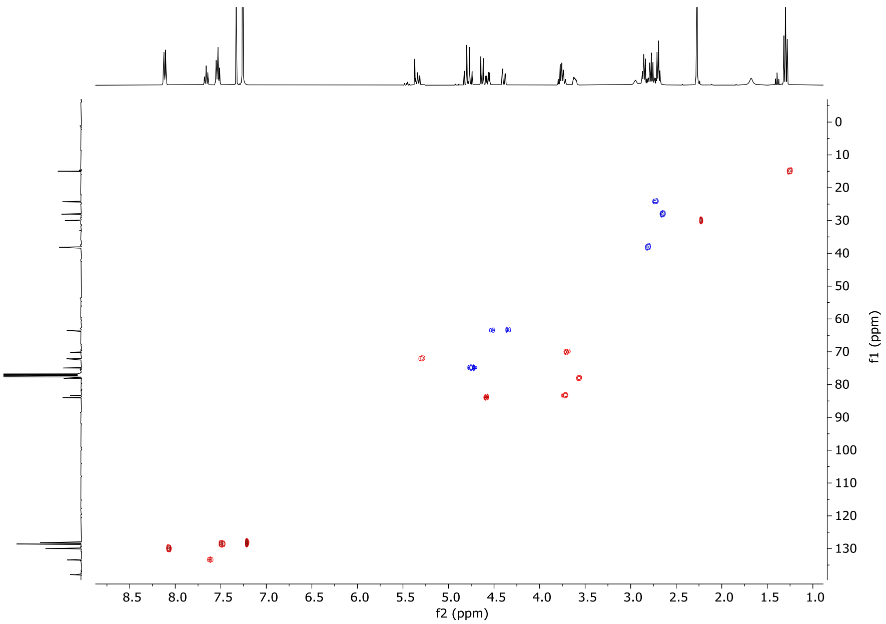

3.5 Ethyl 3-*O*-benzyl-2-*O*-benzoyl-6-*O*-levulinoyl-4-*O*-(9-fluorenylmethoxycarbonyl)-1-thio-β-glucopyranoside, BB8

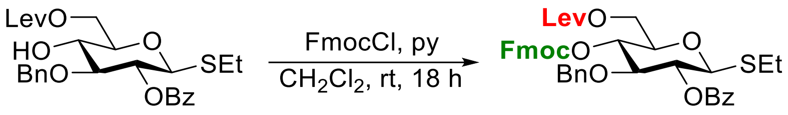

S5 **BB8**

Compound **S5** (7.3 g, 14.1 mmol) was dissolved in CH_2Cl_2 (100 mL) and pyridine was added (3.5 mL, 42.4 mmol). FmocCl (7.3 g, 28.3 mmol) was dissolved in CH_2Cl_2 (100 mL) and added to the reaction mixture. The yellow solution was stirred for 3 h and then quenched with 1 M solution of HCl. The organic layer was washed one time with 1 M HCl, one time with sat. aq. solution of $NaHCO_3$ and one time with brine. The crude compound was purified with flash column chromatography (Hexanes : EtOAc = 2:1) to give compound **BB8** as white solid (6.7 g, 10.7 mmol, 68%). ^1H NMR (400 MHz, $CDCl_3$) δ 8.29 – 8.19 (m, 2H), 7.97 (ddt, *J* = 7.2, 6.3, 0.9 Hz, 2H), 7.87 – 7.78 (m, 3H), 7.68 (t, *J* = 7.8 Hz, 2H), 7.65 – 7.57 (m, 2H), 7.55 – 7.47 (m, 2H), 7.33 – 7.22 (m, 5H), 5.57 (dd, *J* = 10.0, 9.1 Hz, 1H), 5.22 (dd, *J* = 10.1, 9.3 Hz, 1H), 4.85 – 4.66 (m, 4H), 4.61 (dd, *J* = 10.5, 7.2 Hz, 1H), 4.53 – 4.40 (m, 3H), 4.12 (t, *J* = 9.2 Hz, 1H), 3.97 (m, 1H), 3.06 – 2.89 (m, 4H), 2.89 – 2.76 (m, 2H), 2.40 (s, 3H), 1.45 (d, *J* = 7.4 Hz, 3H); ^{13}C NMR (101 MHz, $CDCl_3$) δ 206.5, 172.4, 165.0, 154.2, 143.3, 143.1, 141.3, 141.3, 137.2, 133.4, 129.9, 129.6, 128.5, 128.2, 128.0, 128.0, 127.9, 127.7, 127.3, 127.3, 125.1, 125.0, 120.1, 120.1, 83.8, 80.9, 75.8, 74.5, 74.4, 71.8, 70.2, 62.7, 46.7, 37.9, 29.9, 27.9, 24.2, 14.9; $[\alpha]_D^{20}$ = +29.48; IR (neat) ν_{max}= 3661, 2982, 1463, 1383, 1252, 1153, 1073, 955, 816; R_f = 0.5 (SiO_2, Hexanes : EtOAc = 2:1); HRMS (QToF): Calcd for $C_{42}H_{42}O_{10}SNa$ $[M+Na]^+$ 761.2396; found 761.2405.

^{1}H NMR of BB8 (400 MHz, $CDCl_3$)

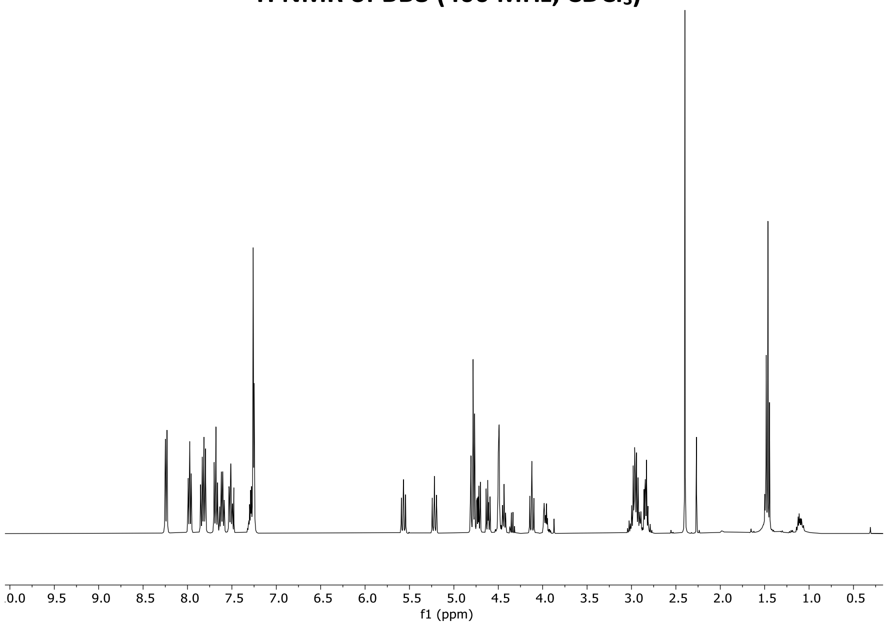

^{13}C NMR of BB8 (101 MHz, $CDCl_3$)

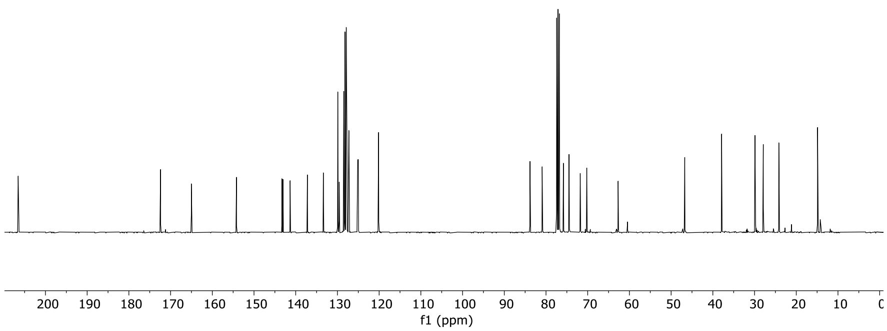

HSQC NMR of BB8 ($CDCl_3$)

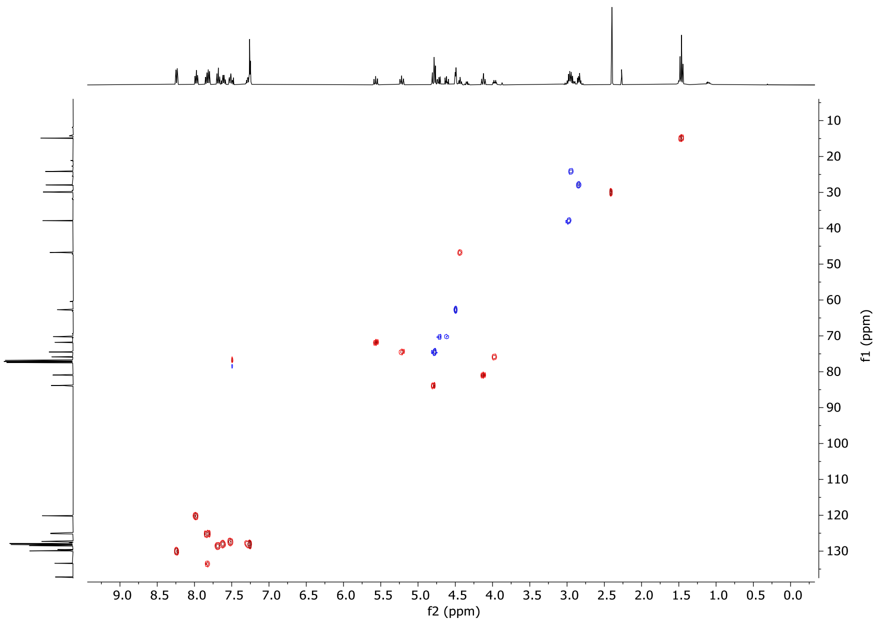

4 Synthesis of BB15b

4.1 Dibutoxyphosphoryloxy 3-*O*-benzyl-4-*O*-(9-fluorenylmethoxycarbonyl)-2-deoxy-2-*N*-trichloroacetyl-6-*O*-levulinoyl-α-glucopyranoside, BB15b

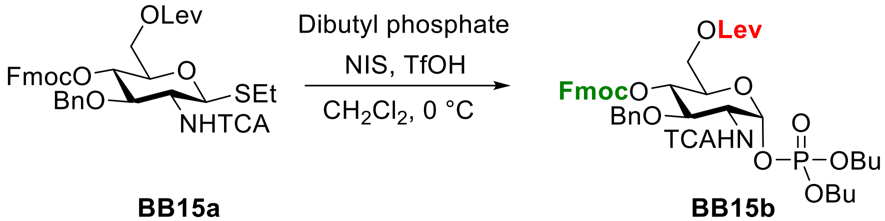

BB15a **BB15b**

An oven dried round bottom flask containing a solution of thioglycoside **BB15a** (0.5 g, 0.64 mmol) and dibutyl hydrogen phosphate (0.25 mL, 1.28 mmol, 2.0 equiv) in anhydrous CH_2Cl_2 (7 mL) was cooled to 0 °C under Ar atmosphere. After 15 min, *N*-iodosuccinimide (215 mg, 0.96 mmol, 1.5 equiv.) was added followed by the dropwise addition of TfOH (6 µL, 0.06 mmol, 0.1 equiv.) at 0 °C. The reaction progress was checked every 30 min until the starting material was fully consumed. The reaction mixture was diluted with CH_2Cl_2 (50 mL) and a sodium thiosulfate solution (10% w/w in water, 20 mL) was added. The organic layer was then separated, washed with a $NaHCO_3$ saturated solution (20 mL), dried over Na_2SO_4, filtered, and concentrated. Compound **BB15b** was obtained after purification by column chromatography (SiO_2, Hexanes : EtOAc = 2:1) as a white solid (0.25 g, 0.27 mmol, 42%). ^{1}H NMR (600 MHz, $CDCl_3$) δ 7.75 (ddt, *J* = 13.1, 7.6, 0.9 Hz, 2H), 7.59 (ddd, *J* = 22.7, 7.5, 1.0 Hz, 2H), 7.43 – 7.36 (m, 2H), 7.29 (tdd, *J* = 7.5, 3.2, 1.1 Hz, 2H), 7.22 (dd, *J* = 5.0, 1.9 Hz, 3H), 7.16 (hept, *J* = 3.0 Hz, 2H), 6.84 (d, *J* = 8.8 Hz, 1H), 5.72 (dd, *J* = 5.9, 3.3 Hz, 1H), 5.07 (dd, *J* = 10.2, 9.2 Hz, 1H), 4.61 – 4.49 (m, 3H), 4.39 – 4.29 (m, 3H), 4.22 – 4.14 (m, 3H), 4.14 – 4.01 (m, 5H), 3.94 (dd, *J* = 10.6, 9.2 Hz, 1H), 2.75 (qt, *J* = 18.4, 6.5 Hz, 2H), 2.69 – 2.53 (m, 2H), 2.18 (s, 3H), 1.66 (q, *J* = 7.5 Hz, 4H), 1.41 – 1.34 (m, 4H), 0.93 (q, *J* = 7.5 Hz, 6H); ^{13}C NMR (151 MHz, $CDCl_3$) δ 206.6, 172.4, 162.0, 154.0, 143.3, 142.9, 141.4, 141.4, 141.3, 136.9, 128.6, 128.5, 128.1, 128.1, 128.0, 128.0, 127.9, 127.3, 125.2, 125.0, 120.2, 120.2, 95.4, 95.4, 92.0, 75.9, 74.3, 73.7, 70.3, 69.7, 68.5, 68.5, 68.4, 61.6, 54.2, 54.2, 46.7, 37.9, 32.3, 32.3, 32.2, 30.0, 27.8, 18.8, 18.7, 13.7, 13.7, 13.7; ^{31}P NMR (243 MHz, D_2O) δ -2.64; $[\alpha]_D^{20}$ = + 0.19; IR (neat) ν_{max}= 3255, 2961, 2928, 2876, 1750, 1717, 1521, 1452, 1358, 1256, 1154, 1107, 1058, 1027, 955, 838, 823, 784, 760, 742; R_f = 0.37 (SiO_2, Hexanes : EtOAc = 1:1); HRMS (QToF): Calcd for $C_{43}H_{51}Cl_3NO_{13}PNa$ $[M+Na]^+$ 948.2061; found 948.2087.

^{1}H NMR of BB15b (600 MHz, $CDCl_3$)

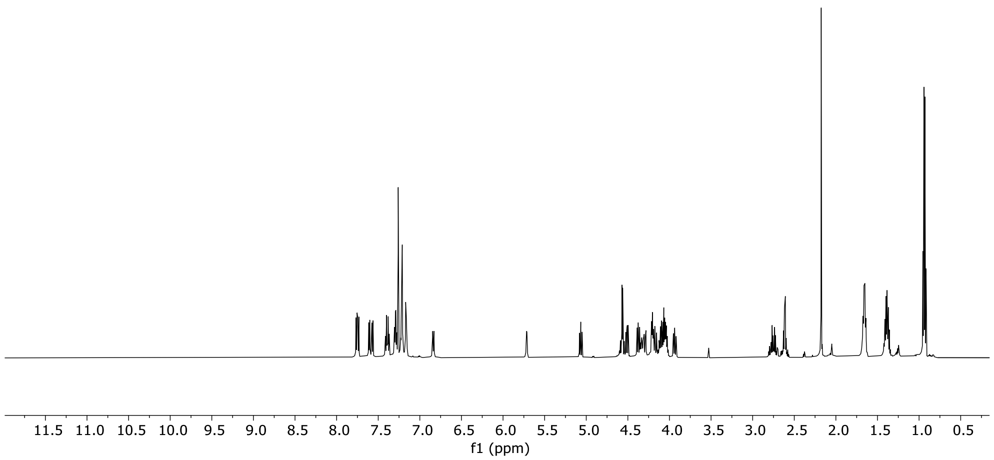

^{13}C NMR of BB15b (151 MHz, $CDCl_3$)

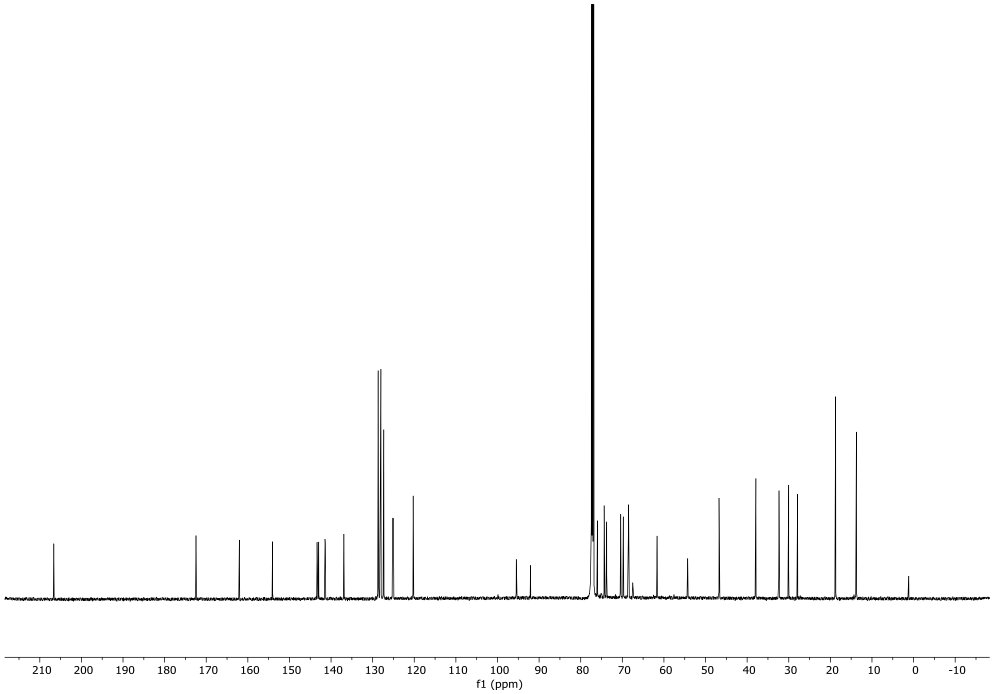

HSQC NMR of BB15b ($CDCl_3$)

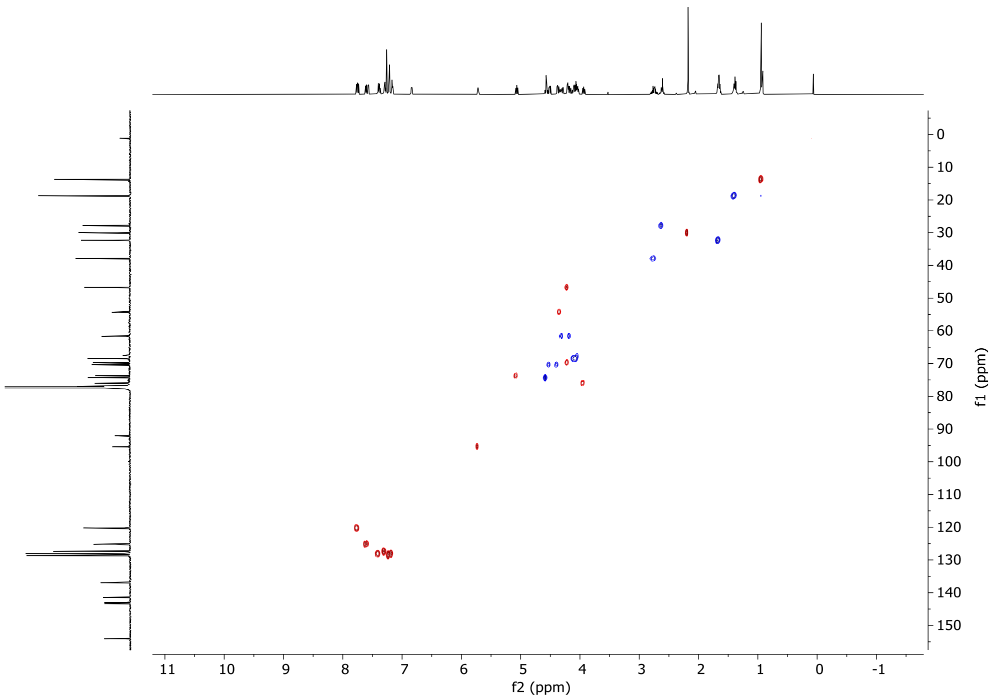

^{31}P NMR of BB15b (243 MHz, $CDCl_3$)

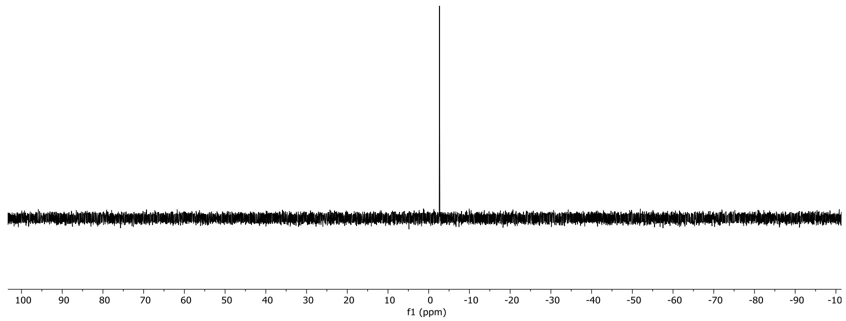

5 Synthesis of BB16

5.1 Dibutoxyphosphoryloxy 3-*O*-benzyl-4-*O*-benzyl-6-*O*-(9-fluorenylmethoxycarbonyl) -2-deoxy-2-*N*-trichloroacetyl-α -glucopyranoside, BB16

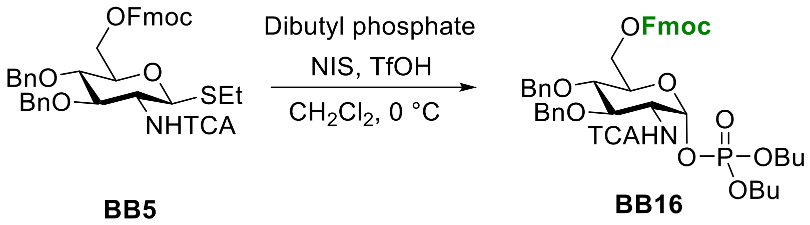

An oven dried round bottom flask containing a solution of thioglycoside **BB5** (0.05 g, 0.065 mmol) and dibutyl hydrogen phosphate (0.025 mL, 0.13 mmol, 2.0 equiv) in anhydrous CH_2Cl_2 (0.7 mL) was cooled to 0 °C under Ar atmosphere. After 15 min, *N*-iodosuccinimide (22 mg, 0.09 mmol, 1.5 equiv.) was added followed by the dropwise addition of TfOH (0.5 µL, 0.0065 mmol, 0.1 equiv.) at 0 °C. The reaction progress was checked every 30 min until the starting material was fully consumed. The reaction mixture was diluted with CH_2Cl_2 (50 mL) and a sodium thiosulfate solution (10% w/w in water, 20 mL) was added. The organic layer was then separated, washed with a $NaHCO_3$ saturated solution (20 mL), dried over Na_2SO_4, filtered, and concentrated. Compound **BB16** was obtained after purification by column chromatography (SiO_2, Hexanes : EtOAc = 2:1) as a white solid (0.046 g, 0.050 mmol, 77%). ^{1}H NMR (700 MHz, $CDCl_3$) δ 7.77 (dd, *J* = 7.6, 2.6 Hz, 2H), 7.64 – 7.59 (m, 2H), 7.43 – 7.39 (m, 2H), 7.35 – 7.29 (m, 11H), 6.91 (t, *J* = 10.2 Hz, 1H), 5.69 (dd, *J* = 5.9, 3.3 Hz, 1H, α -H1), 4.89 – 4.75 (m, 3H), 4.62 (d, *J* = 10.9 Hz, 1H), 4.44 – 4.30 (m, 5H), 4.26 (q, *J* = 8.5 Hz, 1H), 4.14 – 4.03 (m, 5H), 3.92 (dd, *J* = 10.6, 8.9 Hz, 1H), 3.78 (dd, *J* = 10.2, 8.9K Hz, 1H), 1.65 (dt, *J* = 14.8, 7.2 Hz, 4H), 1.41 – 1.33 (m, 4H), 0.91 (td, *J* = 7.4, 5.1 Hz, 6H); ^{13}C NMR (176 MHz, $CDCl_3$) δ 162.1, 155.0, 143.5, 143.3, 143.3, 141.5, 141.4, 137.5, 137.4, 128.8, 128.7, 128.72, 128.3, 128.2, 128.2, 128.2, 128.10, 128.04, 127.3, 125.3, 125.2, 120.2, 96.0, 95.9, 92.3, 79.2, 75.7, 75.4, 71.4, 70.2, 68.46, 65.7, 54.8, 46.8, 32.4, 32.4, 32.3, 32.3, 29.8, 18.7, 13.70; $[\alpha]_D^{20}$ = + 19.19; ^{31}P NMR (243 MHz, D_2O) δ -2.41; IR (neat) ν_{max}= 2931, 2358, 2213, 1752, 1717, 1515, 1454, 1258, 1028, 962, 697, 680, 663; R_f = 0.37 (SiO_2, Hexanes : EtOAc = 2:1); HRMS (QToF): Calcd for $C_{45}H_{51}Cl_3NO_{11}PNa$ $[M+Na]^+$ 940.2163; found 940.2272.

^{1}H NMR of BB16 (700 MHz, $CDCl_3$)

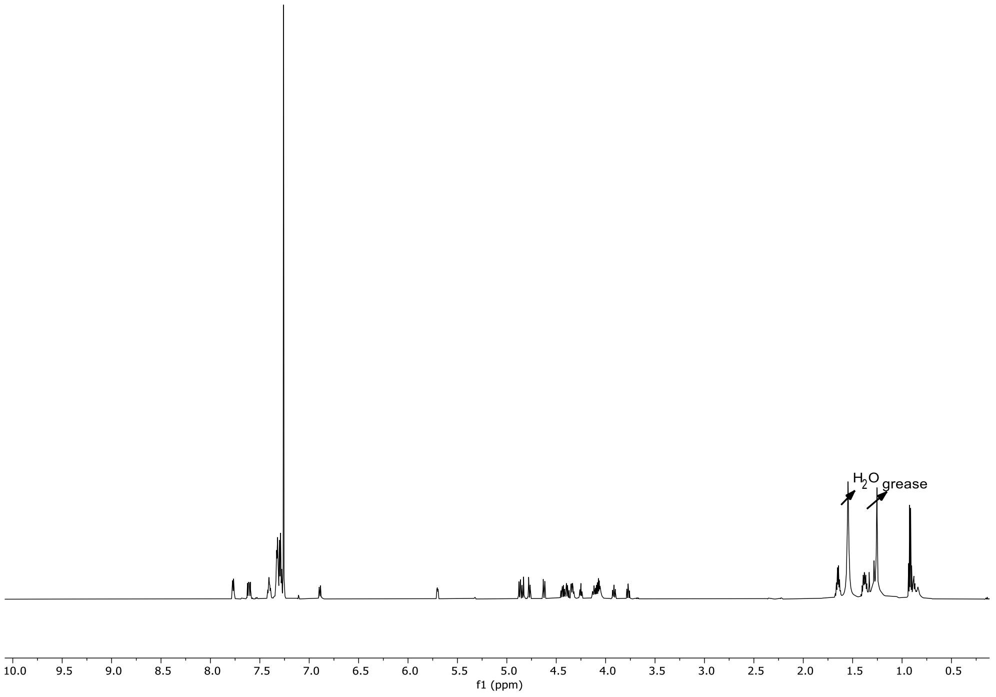

^{13}C NMR of BB16 (176 MHz, $CDCl_3$)

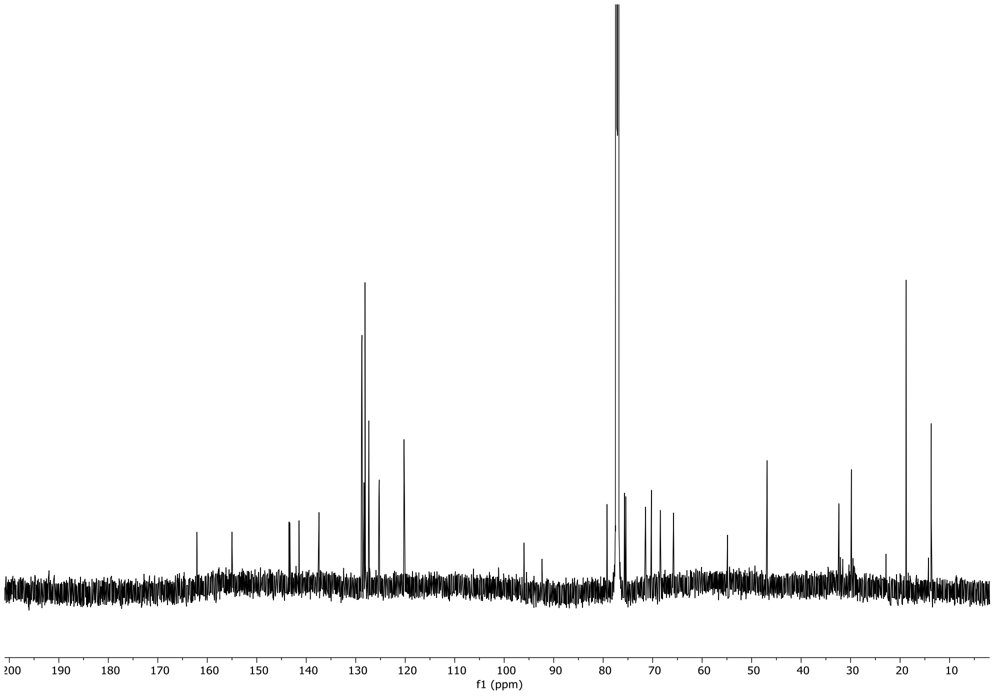

HSQC NMR of BB16 ($CDCl_3$)

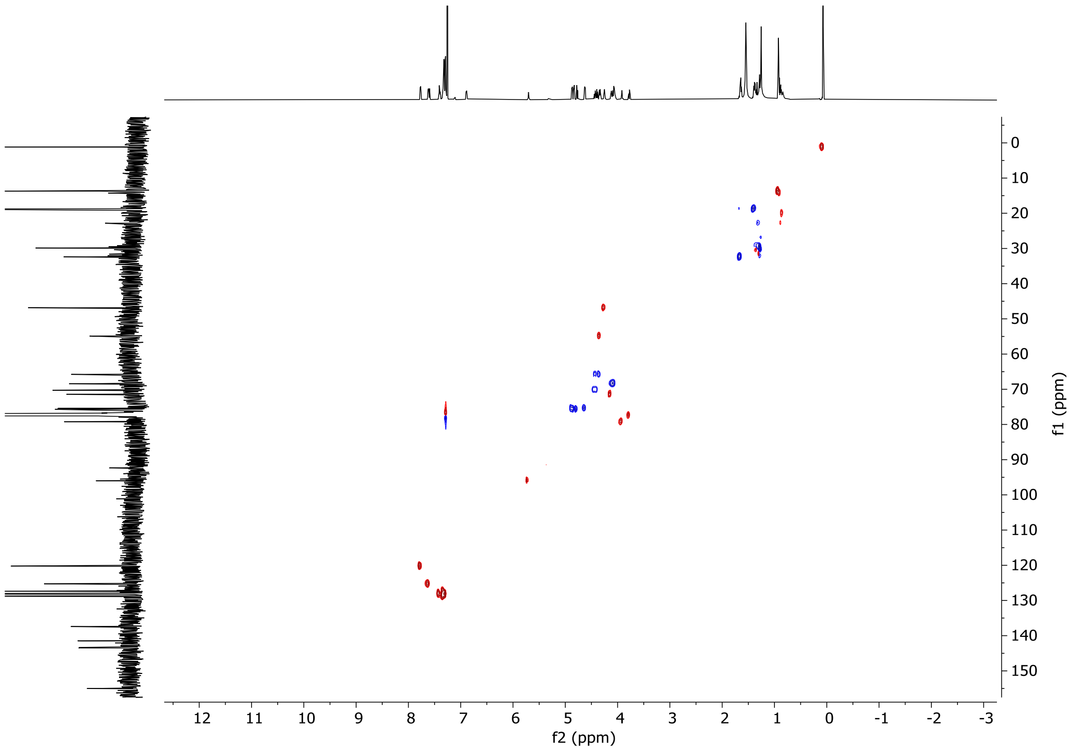

^{31}P NMR of BB16 (243 MHz, $CDCl_3$)

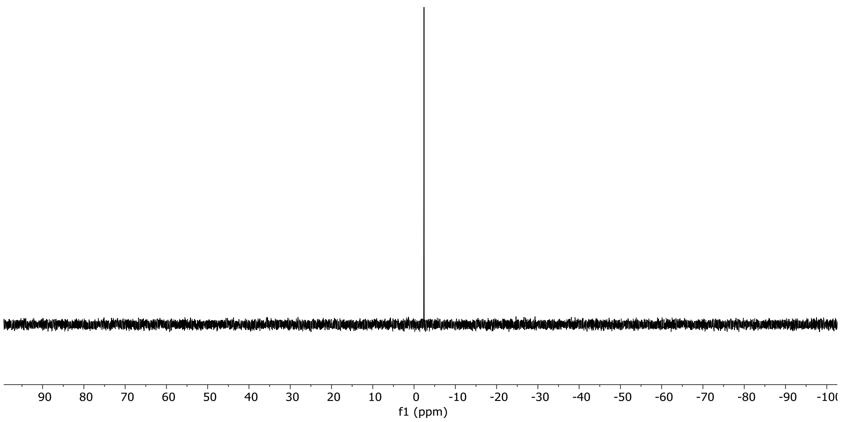

6 Synthesis of BB17

6.1 Phenyl 2-deoxy-2-*N*-trichloroacetylamino-3-O-levulinoyl-1-thio-β-galactopyranoside, S7

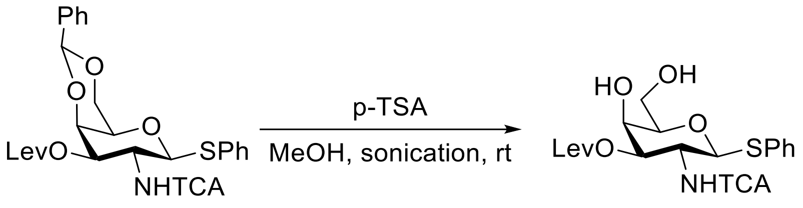

S6 **S7**

S6 was obtained following previously establish procedures.[4]

p-Toluenesulfonic acid (0.21 g, 1.1 mmol, 0.2 equiv) was added to a suspension of **S6** (3.4 g, 5.65 mmol) in MeOH (28 mL, 0.2 M) The reaction mixture was sonicated for 1.5 h at rt and monitored for completion by TLC (30% EtOAc in Hexanes). The reaction was then quenched with triethylamine until neutral. The solvent was removed and compound **S7** as obtained after purification by column chromatography (SiO_2, Hexanes : EtOAc = 1:1 to 0:1) as a white solid (2.1 g, 4.1 mmol, 72%). ^1H NMR (400 MHz, $CDCl_3$) δ 7.54 – 7.47 (m, 2H), 7.34 – 7.27 (m, 3H), 6.91 (d, *J* = 9.0 Hz, 1H), 5.16 (dd, *J* = 10.7, 3.0 Hz, 1H), 5.00 (d, *J* = 10.4 Hz, 1H), 4.29 (dd, *J* = 10.9, 9.0 Hz, 1H), 4.22 (t, *J* = 3.4 Hz, 1H), 4.01 – 3.92 (m, 1H), 3.92 – 3.83 (m, 1H), 3.68 (t, *J* = 5.3 Hz, 1H), 3.29 (d, *J* = 3.6 Hz, 1H), 3.14 (s, 1H), 2.80 – 2.73 (m, 2H), 2.66 – 2.43 (m, 3H), 2.17 (s, 3H); ^{13}C NMR (101 MHz, $CDCl_3$) δ 208.2, 172.4, 161.9, 132.7, 132.4, 129.3, 128.4, 92.5, 86.5, 78.2, 73.8, 67.5, 62.9, 51.2, 38.3, 30.0, 28.3. $[\alpha]_D^{20}$ = +46.85; IR (neat) ν_{max}= 3335, 2941, 1697, 1526, 1480, 1366, 1275, 1148, 1067, 819, 741, 690; R_f = 0.1 (SiO_2, Hexanes : EtOAc = 1:1); HRMS (QToF): Calcd for $C_{19}H_{22}Cl_3NO_7SNa$ $[M+Na]^+$ 536.0075; found 536.0075.

^1H NMR of S7 (400 MHz, $CDCl_3$)

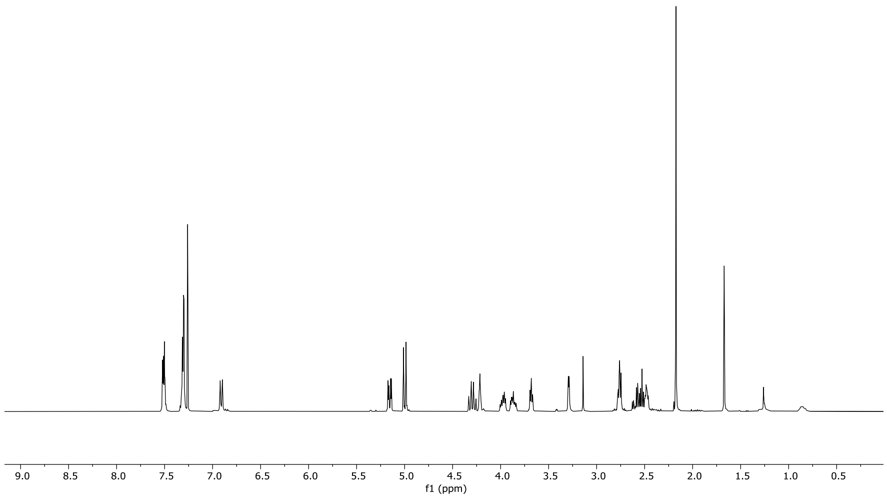

^{13}C NMR of S7 (101 MHz, $CDCl_3$)

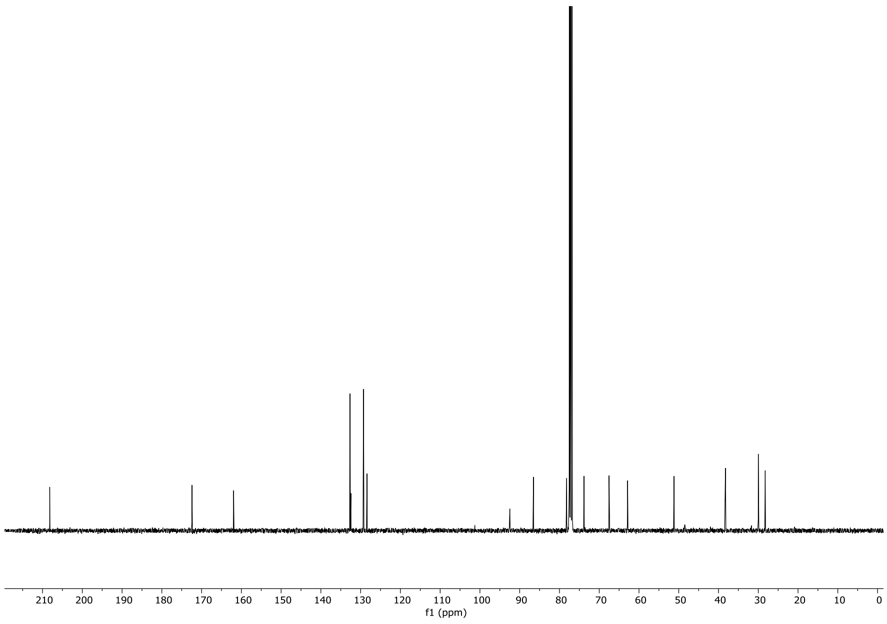

HSQC NMR of S7 ($CDCl_3$)

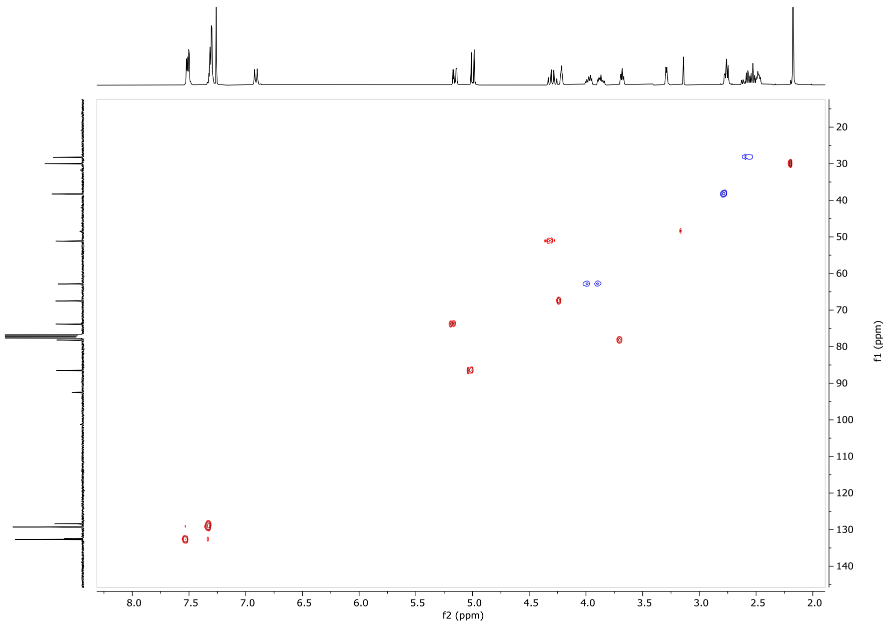

6.2 Phenyl 4,6-di-*O*-(9-fluorenylmethoxycarbonyl)-2-deoxy-2-*N*-trichloroacetylamino-3-*O*-levulinoyl-1-thio-β-galactopyranoside, S8

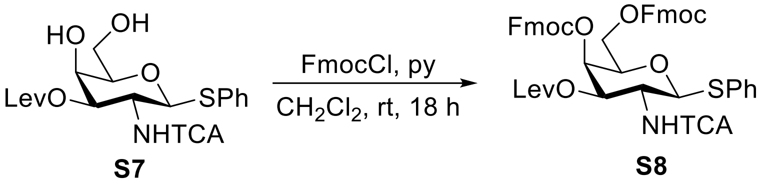

Pyridine (5 mL, 61.2 mmol, 15 equiv) and FmocCl (3.1 g, 12.2 mmol, 3 equiv) were added to a solution of **S7** (2.1 g, 4.1 mmol) in CH_2Cl_2 (40 mL, 0.1 M). The reaction mixture was stirred overnight at rt and monitored for completion by TLC (100% EtOAc). The solvent was removed and compound **S8** was obtained after purification by column chromatography (SiO_2, Hexanes : EtOAc = 1:1 to 0:1, containing 10% CH_2Cl_2) as a yellow solid (3.3 g, 84%). ^1H NMR (400 MHz, $CDCl_3$) δ 7.77 (t, *J =* 7.0 Hz, 4H), 7.66 – 7.55 (m, 6H), 7.45 – 7.26 (m, 12H), 6.86 (d, *J* = 8.7 Hz, 1H), 5.41 (dd, *J* = 10.8, 3.2 Hz, 1H), 5.30 (d, *J* = 3.4 Hz, 1H), 5.08 (d, *J* = 10.4 Hz, 1H), 4.48 – 4.35 (m, 5H), 4.34 – 4.17 (m, 4H), 4.09 – 4.04 (m, 1H), 2.69 – 2.29 (m, 4H), 2.03 (s, 3H)*;* ^{13}C NMR (101 MHz, $CDCl_3$) δ 206.3, 172.1, 161.9, 154.8, 154.8, 143.4, 143.3, 143.3, 143.1, 141.4, 141.4, 133.0, 132.1, 129.2, 128.6, 128.2, 128.1, 128.1, 127.5, 127.5, 127.4, 127.3, 125.5, 125.3, 125.3, 120.3, 120.2, 120.2, 92.3, 86.4, 74.6, 71.2, 70.7, 70.4, 65.4, 51.5, 46.8, 46.7, 37.7, 29.8, 29.7, 27.9; $[\alpha]_D^{20}$ = + 0.63; IR (neat) ν_{max}= 2385, 2359, 2344, 1748, 1525, 1450, 1241, 1147, 819, 783, 759, 738; R_f = 0.57 (SiO_2, Hexanes : EtOAc = 1:1); HRMS (QToF): Calcd for $C_{49}H_{42}Cl_3NO_{11}SNa$ $[M+Na]^+$ 980.1436; found 980.1507.

^{1}H NMR of S8 (400 MHz, $CDCl_3$)

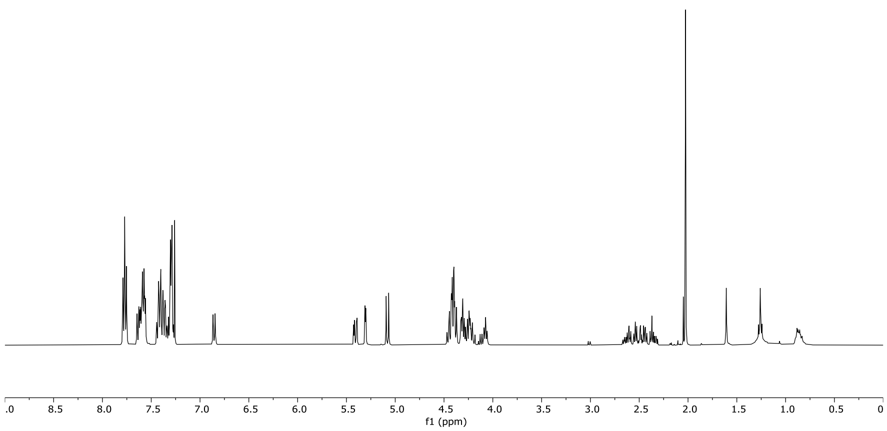

^{13}C NMR of S8 (101 MHz, $CDCl_3$)

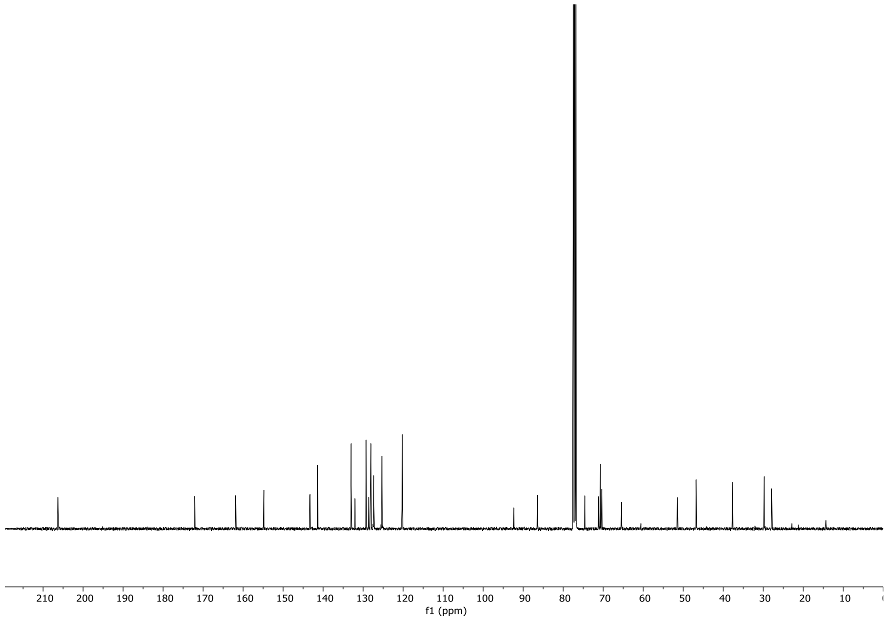

HSQC NMR of S8 ($CDCl_3$)

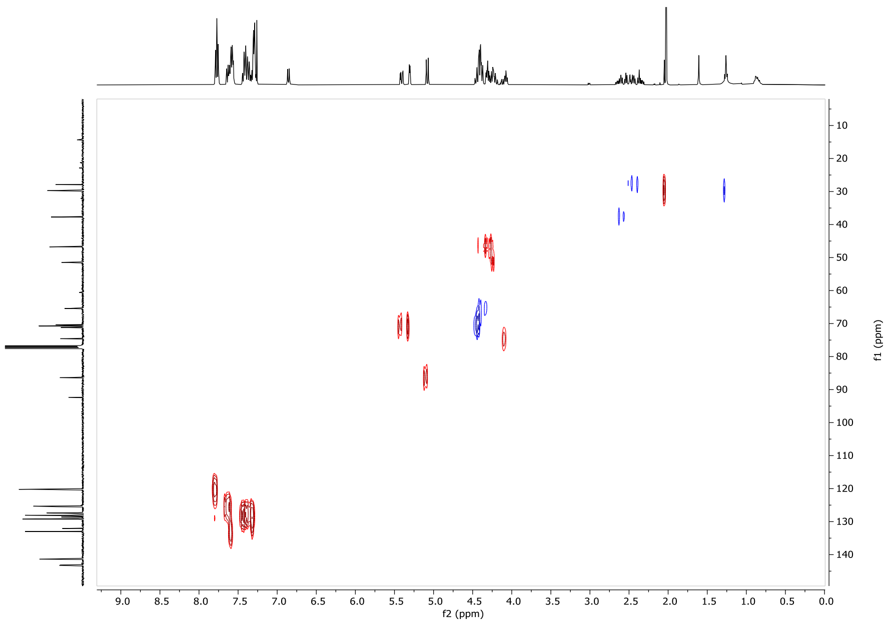

6.3 Dibutoxyphosphoryloxy 4,6-di-*O*-(9-fluorenylmethoxycarbonyl)-2-deoxy-2-*N*-trichloroacetylamino-3-*O*-levulinoyl-α/β-galactopyranoside, BB17

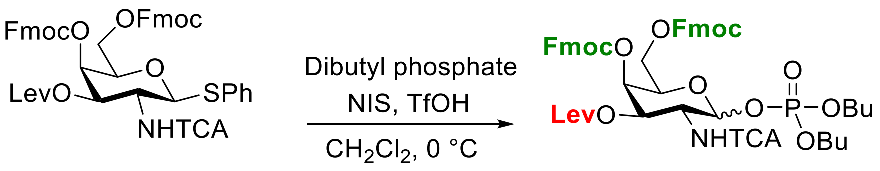

S8 **BB17**

An oven dried round bottom flask containing a solution of thioglycoside **S8** (1.5 g, 1.56 mmol) and dibutyl hydrogen phosphate (0.62 mL, 3.13 mmol, 2.0 equiv) in anhydrous CH_2Cl_2 (15 mL) was cooled to 0 °C under Ar atmosphere. After 15 min, *N*-iodosuccinimide (525 mg, 2.35 mmol, 1.5 equiv.) was added followed by the dropwise addition of TfOH (15 µL, 0.16 mmol, 0.1 equiv.) at 0 °C. The reaction progress was checked every 30 min until the starting material was fully consumed after 1 h. The reaction mixture was diluted with CH_2Cl_2 (50 mL) and a sodium thiosulfate solution (10% w/w in water, 20 mL) was added. The organic layer was then separated, washed with sat. aq. solution of $NaHCO_3$ (20 mL), dried over Na_2SO_4, filtered, and concentrated. **BB17** was obtained after purification by column chromatography (SiO_2, Hexanes : EtOAc = 2:1) as a white solid (1.1 g, 2.06 mmol, α:β = 3:7, 66%). ^{1}H NMR (400 MHz, $CDCl_3$) δ 7.80 – 7.73 (m, 4H), 7.67 (d, *J* = 6.8 Hz, 1H), 7.59 (dt, *J* = 13.9, 7.2 Hz, 3H), 7.43 – 7.30 (m, 8H), 6.98 (d, *J* = 9.9 Hz, 1H), 5.88 (d, *J* = 4.9 Hz, 1H, α-H1), 5.50 (t, *J* = 7.9 Hz, 1H, β-H1), 5.38 (d, *J* = 8.1 Hz, 1H), 5.30 (d, *J* = 9.4 Hz, 1H), 4.62 – 4.47 (m, 1H), 4.48 – 4.31 (m, 7H), 4.24 (d, *J* = 7.5 Hz, 1H), 4.19 – 3.98 (m, 5H), 2.66 – 2.30 (m, 4H), 2. 2.04 (s, 3H), 1.70 – 1.63 (m, 4H), 1.40 (p, *J* = 8.1 Hz, 4H), 0.92 (t, *J* = 7.3 Hz, 6H). ^{13}C NMR (151 MHz, $CDCl_3$) δ 205.9, 205.9, 172.5, 172.0, 162.6, 162.2, 154.7, 154.7, 154.6, 154.6, 143.3, 143.2, 143.2, 143.2, 143.1, 143.0, 142.9, 141.3, 141.2, 141.2, 141.2, 128.1, 128.05, 128.03, 128.01, 127.95, 127.93, 127.49, 127.47, 127.4, 127.3, 127.24, 127.22, 125.4, 125.3, 125.2, 125.2, 125.1, 120.1, 120.1, 120.0, 96.59, 96.56, 95.60, 95.5, 92.4, 91.0, 71.6, 70.9, 70.8, 70.7, 70.46, 70.44, 70.3, 70.2, 68.64, 68.62, 68.5, 68.47, 68.43, 68.41, 68.37, 67.8, 67.3, 65.0, 64.8, 52.3, 52.2, 49.9, 49.8, 46.6, 46.51, 46.4, 37.5, 37. 32.23, 32.20, 32.1, 32.03, 32.01, 31.9, 29.7, 29.6, 29.3, 27.8, 22.72, 18.7, 18.64, 18.63, 18.6, 14.1, 13.6, 13.5; ^{31}P NMR (243 MHz, D_2O) δ -3.34; IR (neat) v_{max}= 2960, 1751, 1719, 1529, 1451, 1385, 1247, 1026, 964, 822, 758, 739; R_f = 0.5 and 0.3 (SiO_2, Hexanes : EtOAc = 1:1); HRMS (QToF): Calcd for $C_{51}H_{55}Cl_3NO_{15}PNa$ $[M+Na]^+$ 1080.2267; found 1080.2279.

^{1}H NMR of BB17 (400 MHz, $CDCl_3$)

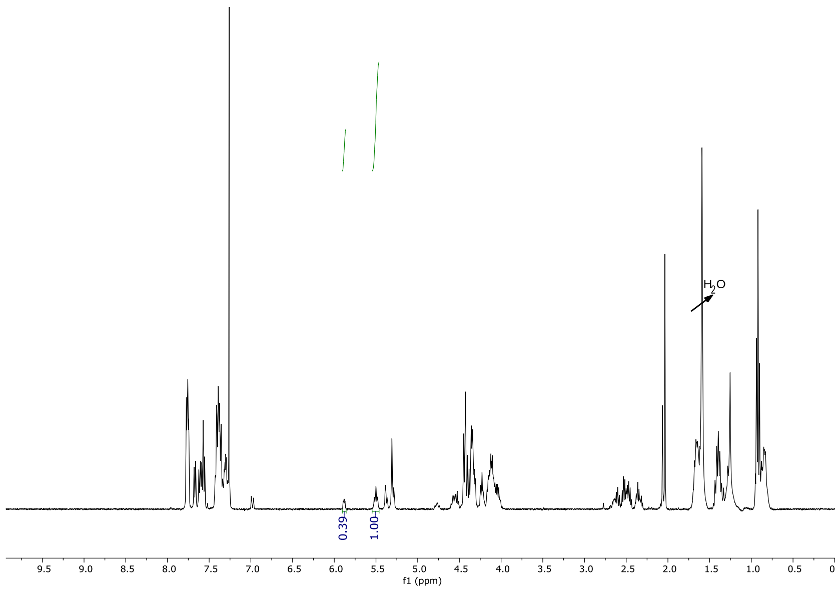

^{13}C NMR of BB17 (151 MHz, $CDCl_3$)

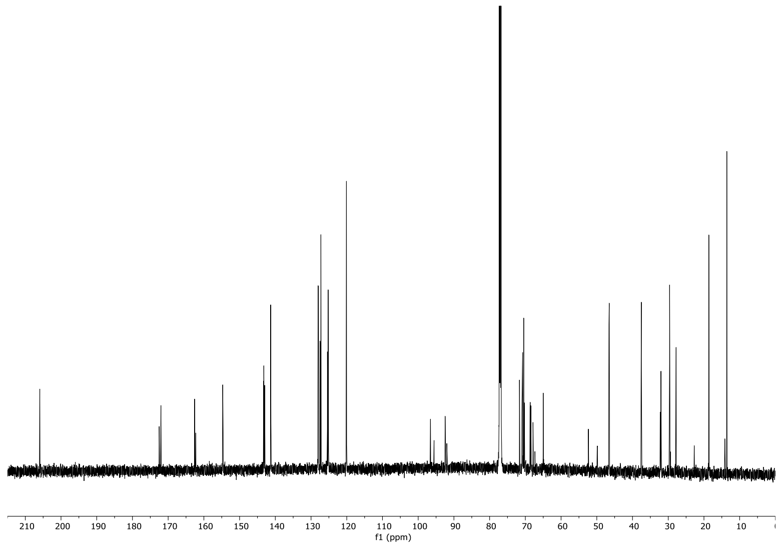

HSQC NMR of BB17 ($CDCl_3$)

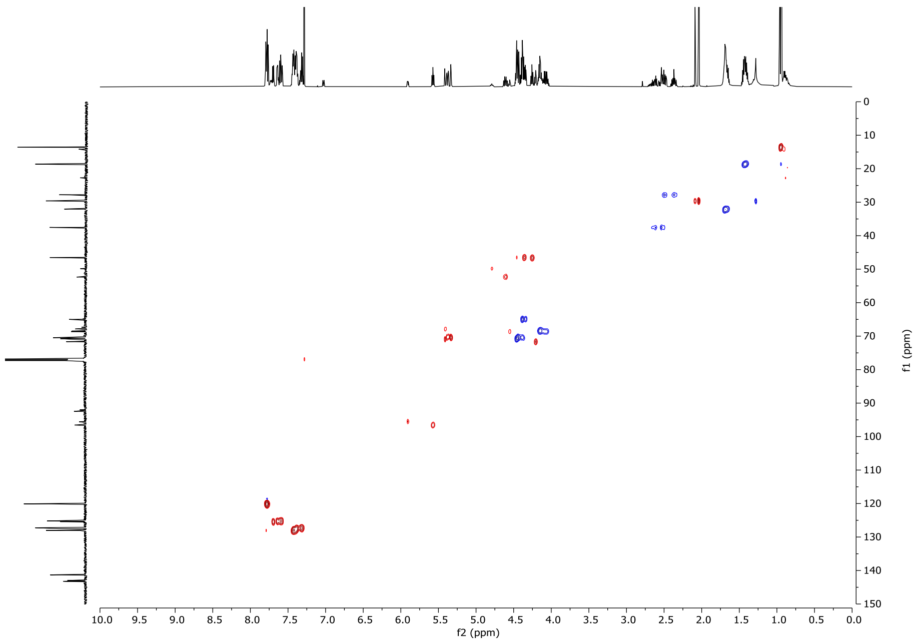

^{31}P NMR of BB17 (243 MHz, $CDCl_3$)

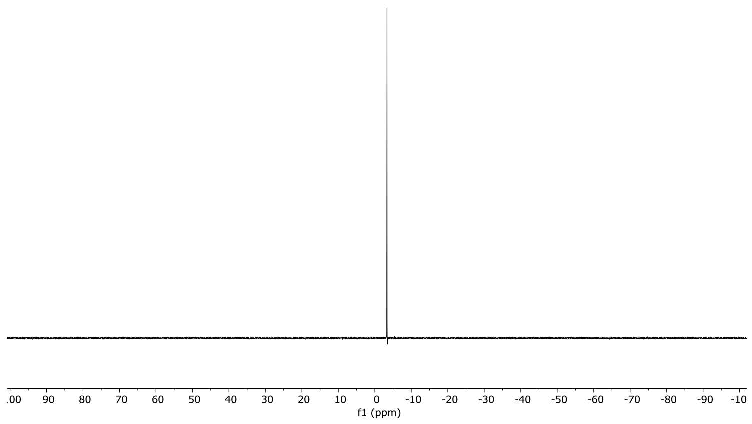

7 Synthesis of BB18b

7.1 Methyl (Dibutoxyphosphoryloxy 4-O-levulinoyl-3-O-benzyl-2-O-benzoyl-β-glucopyranosyluronate), BB20

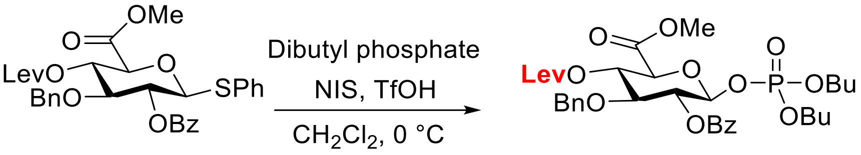

BB18a **BB18b**

An oven dried round bottom flask containing a solution of thioglycoside **BB18a*** (1.0 g, 1.69 mmol) and dibutyl hydrogen phosphate (0.67 mL, 3.38 mmol, 2.0 equiv) in anhydrous CH_2Cl_2 (16 mL) was cooled to 0 °C under Ar atmosphere. After 15 min, *N*-iodosuccinimide (567 mg, 2.53 mmol, 1.5 equiv.) was added followed by the dropwise addition of TfOH (15 µL, 0.16 mmol, 0.1 equiv.) at 0 °C. The reaction progress was checked every 30 min until the starting material was fully consumed. The reaction mixture was diluted with CH_2Cl_2 (50 mL) and a sodium thiosulfate solution (10% w/w in water, 20 mL) was added. The organic layer was then separated, washed with a $NaHCO_3$ saturated solution (20 mL), dried over Na_2SO_4, filtered, and concentrated. Compound **BB18b** was obtained after purification by column chromatography (SiO_2, Hexanes : EtOAc = 2:1) as a white solid (1.0 g, 1.4 mmol, 85%). ^{1}H NMR (600 MHz, $CDCl_3$) δ 7.99 (dd, *J* = 8.3, 1.4 Hz, 2H), 7.62 – 7.56 (m, 1H), 7.48 – 7.42 (m, 2H), 7.14 (s, 5H), 5.47 – 5.37 (m, 2H), 5.28 (dd, *J* = 9.9, 9.2 Hz, 1H), 4.67 (d, *J* = 11.8 Hz, 1H), 4.60 (d, *J* = 11.8 Hz, 1H), 4.13 – 3.99 (m, 3H), 3.89 (t, *J* = 9.0 Hz, 1H), 3.73 (s, 3H), 3.71 – 3.62 (m, 2H), 2.71 (td, *J* = 6.4, 1.9 Hz, 2H), 2.58 (ddd, *J* = 17.5, 7.0, 5.9 Hz, 1H), 2.53 – 2.43 (m, 1H), 2.18 (s, 3H), 1.65 – 1.61 (m, 2H), 1.42 – 1.33 (m, 2H), 1.24 (dq, *J* = 9.0, 6.8 Hz, 3H), 0.97 (ddd, *J* = 13.9, 8.3, 7.0 Hz, 2H), 0.89 (t, *J* = 7.4 Hz, 3H), 0.65 (t, *J* = 7.4 Hz, 3H); ^{13}C NMR (151 MHz, $CDCl_3$) δ 206.2, 171.64, 171.60, 167.9, 167.0, 164.8, 153.6, 143.5, 137.2, 133.7, 130.02, 129.1, 128.6, 128.6, 128.4, 128.29, 128.2, 128.0, 96.2, 96.2, 78.5, 74.4, 73.0, 72.4, 72.3, 71.1, 68.4, 68.3, 68.11, 53., 32.0, 31.8, 31.7, 30.0, 27.7, 18.6, 18.3, 13.7, 13.5; $[\alpha]_D^{20}$ = + 46.43 ^{31}P NMR (243 MHz, D_2O) δ -2.93; IR (neat) ν_{max}= 2962, 1722, 1454, 1365, 1267, 1151, 1028, 909, 713; R_f = 0.37 and 0.25 (SiO_2, Hexanes : EtOAc = 1:1); HRMS (QToF): Calcd for $C_{34}H_{45}O_{13}PNa$ $[M+Na]^+$ 715.2490; found 715.2509.

*(purchased from GlycoUniverse)

^{1}H NMR of BB18b (600 MHz, $CDCl_3$)

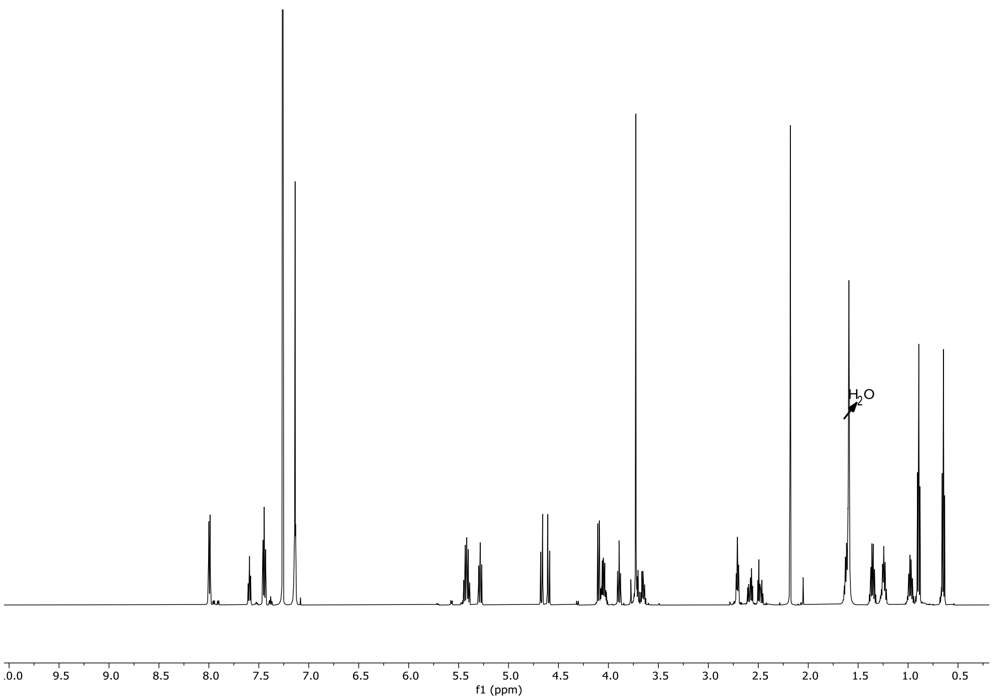

^{13}C NMR of BB18b (151 MHz, $CDCl_3$)

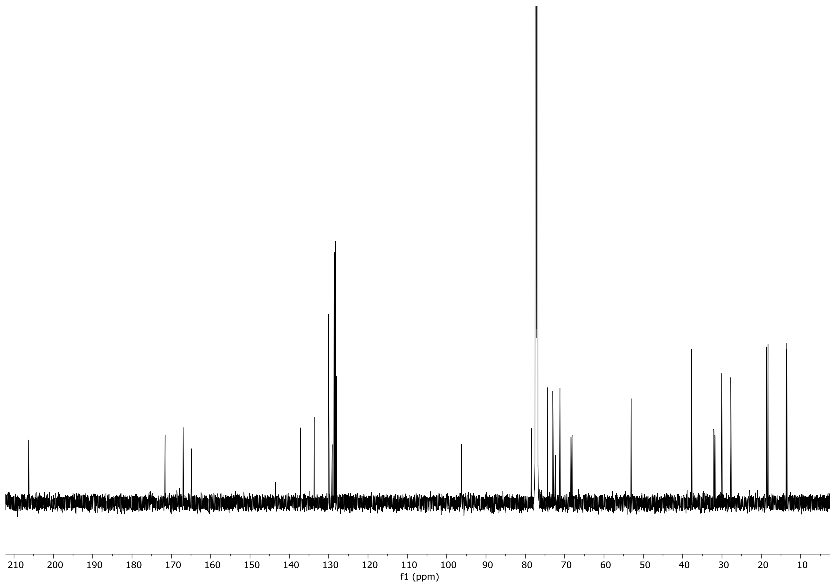

HSQC NMR of BB18b ($CDCl_3$)

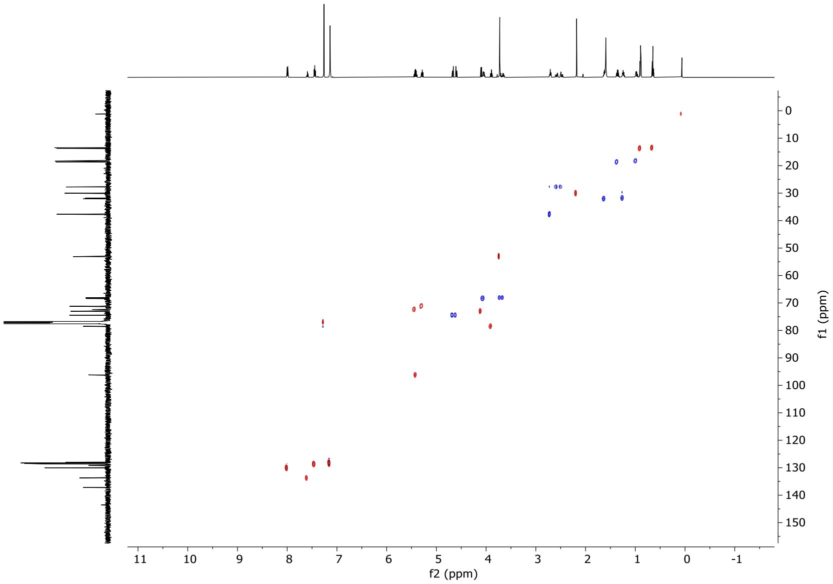

^{31}P NMR of BB18b (243 MHz, $CDCl_3$)

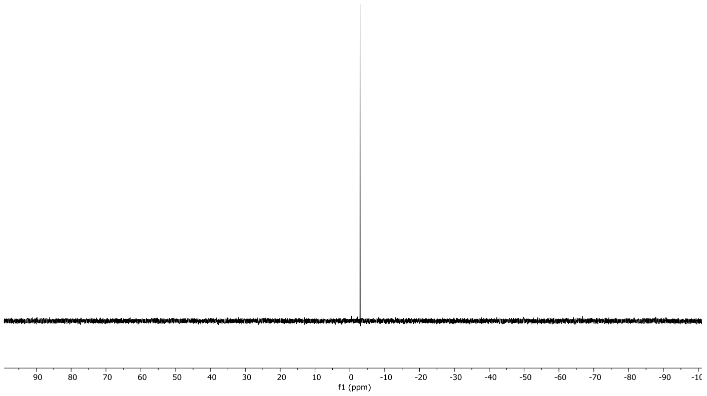

8 Automated Glycan Assembly

8.1 General materials and method

The automated syntheses were performed on a home built synthesizer developed at the Max Planck Institute of Colloids and Interfaces. All solvents used were HPLC-grade. The solvents used for the building block, activator, TMSOTf and capping solutions were taken from an anhydrous solvent system (J.C. Meyer) and further dried with molecular sieves (4 Å) for moisture sensitive solutions. The building blocks were co-evaporated three times with toluene and dried for 1 h on high vacuum before use. Oven dried, argon flushed flasks were used to prepare all moisture sensitive solutions. Activator, capping, deprotection, acidic wash, and building block solutions were freshly prepared and kept under argon during the automation run. All yields of products obtained by AGA were calculated on the basis of resin loading. Resin loading was determined following previously established procedures.

8.2 Preparation of stock solutions

- **Building block solution**: Between 0.06 and 0.10 mmol of building block (depending on the BB, see Module C1 and C2) was dissolved in CH_2Cl_2 (1 mL).
- **NIS/TfOH activator solution**: 1.35 g (6.0 mmol) of recrystallized NIS was dissolved in 40 mL of a 2:1 v/v mixture of anhydrous CH_2Cl_2 and anhydrous dioxane. Then triflic acid (55 µL, 0.6 mmol) was added. The solution was kept at 0 °C for the duration of the automation run.
- **Fmoc deprotection solution**: A solution of 20% piperidine in DMF (v/v) was prepared.
- **Lev deprotection solution**: Hydrazine acetate (550 mg, 5.97 mmol) was dissolved in pyridine/AcOH/H_2O (40 mL, v/v, 32:8:2) and sonicated for 10 min.
- **TMSOTf solution**: TMSOTf (0.45 mL, 2.49 mmol) was added to CH_2Cl_2 (40 mL) or for glycosyl phosphate activation; TMSOTf (0.9 mL, 5.0 mmol) was added to CH_2Cl_2 (40 mL).
- **Capping solution**: A solution of 10% acetic anhydride and 2% methanesulfonic acid in CH_2Cl_2 (v/v) was prepared.

8.3 Modules for automated synthesis

Module A: Resin preparation for synthesis (20 min)

All automated syntheses were performed on 0.0135 mmol scale. Resin (**L1**, 45 mg or **L2**, 35 mg) was placed in the reaction vessel and swollen in CH_2Cl_2 for 20 min at rt prior to synthesis. During this time, all reagent lines needed for the synthesis were washed and primed. After the swelling, the resin was washed with DMF, THF, and CH_2Cl_2 (three times each with 2 mL for 25 s).

Module B: Acidic wash with TMSOTf solution (20 min)

The resin was swollen in 2 mL CH_2Cl_2 and the temperature of the reaction vessel was adjusted to -20 °C. Upon reaching the low temperature, TMSOTf solution (1 mL) was added drop wise to the reaction vessel. After bubbling for 3 min, the acidic solution was drained and the resin was washed with 2 mL CH_2Cl_2 for 25 s.

Action	Cycles	Solution	Amount	T (°C)	Incubation time
Cooling	-	-	-	-20	(15 min)*
Deliver	1	CH_2Cl_2	2 mL	-20	-
Deliver	1	TMSOTf solution	1 mL	-20	3 min
Wash	1	CH_2Cl_2	2 mL	-20	25 sec

*Time required to reach the desired temperature.

Module C1: Thioglycoside glycosylation (35 - 55 min)

The building block solution was delivered to the reaction vessel. After the set temperature was reached, the reaction was started by dropwise addition of the NIS/TfOH activator solution (1.0 mL). After completion of the reaction, the solution was drained and the resin was washed with CH_2Cl_2 , CH_2Cl_2:dioxane (1:2, 3 mL for 20 s) and CH_2Cl_2 (two times, each with 2 mL for 25 s). The temperature of the reaction vessel was increased to 25 °C for the next module.

Action	Cycles	Solution	Amount	T (°C)	Incubation time
Cooling	-	-	-	-20	-
Deliver	1	BB solution	1 mL	-20	-
Deliver	1	NIS/TfOH activator solution	1 mL	-20	-
Reaction time	1			-20<br>to 0	5 min<br>20 min
Wash	1	CH_2Cl_2	2 mL	0	5 sec
Wash	1	CH_2Cl_2: Dioxane (1:2)	2 mL	0	20 sec
Heating	-	-	-	25	-
Wash	2	CH_2Cl_2	2 mL	> 0	25 sec

Module C2: Glycosyl phosphate glycosylation (45 min)

The building block solution (0.06 mmol of BB in 1 mL of CH_2Cl_2 per glycosylation) was delivered to the reaction vessel. After the set temperature was reached, the reaction was started by drop wise addition of the TMSOTf solution (1.0 mL, same equiv). After completion of the reaction, the solution was drained and the resin washed with CH_2Cl_2 (six times, each with 2 mL for 25 s). The temperature of the reaction vessel was increased to 25 °C for the next module.

Action	Cycles	Solution	Amount	T (°C)	Incubation time
Cooling	-	-	-	-30	-
Deliver	1	BB solution	1 mL	-30	-
Deliver	1	TMSOTf solution	1 mL	-30	-
Reaction time	1			-30<br>to -10	5 min<br>40 min
Wash	1	CH_2Cl_2	2 mL	-10	5 sec
Heating	-	-	-	25	-
Wash	6	CH_2Cl_2	2 mL	> 0	25 sec

Module D: Capping (30 min)

The resin was washed with DMF (two times with 2 mL for 25 s) and the temperature of the reaction vessel was adjusted to 25 °C. 2 mL of Pyridine solution (10% in DMF) was delivered into the reaction vessel. After 1 min, the reaction solution was drained and the resin washed with CH_2Cl_2 (three times with 3 mL for 25 s). 4 mL of capping solution was delivered into the reaction vessel. After 20 min, the reaction solution was drained and the resin washed with CH_2Cl_2 (three times with 3 mL for 25 s).

Action	Cycles	Solution	Amount	T (°C)	Incubation time
Heating	-	-	-	25	(5 min)*
Wash	2	DMF	2 mL	25	25 sec
Deliver	1	10% Pyridine in DMF	2 mL	25	1 min
Wash	3	CH_2Cl_2	2 mL	25	25 sec
Deliver	1	Capping Solution	4 mL	25	20 min
Wash	3	CH_2Cl_2	2 mL	25	25 sec

*Time required to reach the desired temperature.

Module E1: Fmoc deprotection (9 min)

The resin was washed with DMF (three times with 2 mL for 25 s) and the temperature of the reaction vessel was adjusted to 25 °C. 2 mL of Fmoc deprotection solution was delivered to the reaction vessel and kept under Ar bubbling. After 5 min, the reaction solution was drained and the resin washed with DMF (three times with 3 mL for 25 s) and CH_2Cl_2 (five times each with 2 mL for 25 s). The temperature of the reaction vessel was decreased to -20 °C for the next module.

Action	Cycles	Solution	Amount	T (°C)	Incubation time
Wash	3	DMF	2 mL	25	25 sec
Deliver	1	Fmoc depr. solution	2 mL	25	5 min
Wash	1	DMF	2 mL		
Cooling	-	-	-	-20	-
Wash	3	DMF	2 mL	< 25	25 sec
Wash	5	CH_2Cl_2	2 mL	< 25	25 sec

Module E2: Lev deprotection (65 min)

The resin was washed with CH_2Cl_2 (three times with 2 mL for 25 s). CH_2Cl_2 (1.3 mL) was delivered to the reaction vessel and the temperature of the reaction vessel was adjusted to 25 °C. 2 mL of Lev deprotection solution was delivered to the reaction vessel that was kept under pulsed Ar bubbling for 30 min. This procedure was repeated twice. The reaction solution was drained and the resin washed with DMF (three times with 3 mL for 25 s) and CH_2Cl_2 (five times each with 2 mL for 25 s).

Action	Cycles	Solution	Amount	T (°C)	Incubation time
Wash	3	DMF	2 mL	25	25 sec
Deliver	2	Lev depr. solution	2 mL	25	30 min
Wash	1	DMF	2 mL		
Cooling	-	-	-	-20	-
Wash	3	DMF	2 mL	< 25	25 sec
Wash	5	CH_2Cl_2	2 mL	< 25	25 sec

Note:

With the current setup the automated synthesizer has four BB lines. Therefore, for AGA of compounds requiring five BBs (*i.e.* AGA of **S-Le^x**) a first cycle with BB**6** was performed and, upon completion, BB**6** was replaced by BB**11** to continue the AGA.

8.4 Post-synthesizer manipulations (Post-AGA)

Module F: On-resin sulfation

The resin was suspended in 4 mL of a 0.5 M SO_3·py solution (DMF/pyridine, 1:1). The reaction was rotated for 12 h at 40 °C, after which time the resin was repeatedly washed with DMF (5 x 4 mL), MeOH (5 x 4 mL) and CH_2Cl_2 (5 x 4 mL).

Module G: On-resin hydrolysis

The resin was suspended in THF:MeOH (4:1, 4 mL) and a solution of LiOH in water (150 µL, 1 M) was added. The mixture was gently shaken at rt. After microcleavage (see Module G1) indicated the complete hydrolysis of all ester groups, the resin was repeatedly washed with MeOH (5 x 4 mL) and CH_2Cl_2 (5 x 4 mL). The reaction time is variable and it is indicated for each synthesis.

Module G1: On-resin acetylation

The resin was suspended in a 4 mL solution of acetic anhydride in DMF (15% v/v) and the mixture gently shaken at rt for 3 h, after which time the resin was repeatedly washed with DMF (5 x 4 mL), MeOH (5 x 4 mL) and CH2Cl2 (5 x 4 mL).

Module H: Cleavage from solid support

The oligosaccharides were cleaved from the solid support using a continuous-flow photoreactor as described previously. A 20% MeOH in CH_2Cl_2 solvent system was used due to the presence of sulfate groups in the glycan.

Module H1: Micro-cleavage from solid support

Trace amount of resin (around 20 beads) was dispersed in CH_2Cl_2 (0.1 mL) and irradiated with a UV lamp (6 watt, 356 nm) for 20 min. ACN was then added to the resin and the resulting solution analyzed by MS-Q-TOF or MALDI.

Module I: Hydrogenolysis at ambient pressure[a]

The crude compound obtained from Module H was dissolved in 2 mL of *t*-BuOH:H_2O (1:1). The Pd catalyst (2.5 times the weight of the starting material) was added and the reaction was stirred in a flask equipped with a H_2 balloon. The reaction progress was monitored to avoid undesired side products formation. Upon completion, the reaction was filtered and washed with *t*-BuOH and H_2O. The filtrates were concentrated *in vacuo.*

Module I1: Hydrogenolysis[a]

The crude compound obtained from Module H was dissolved in 2 mL of *t*-BuOH:H_2O (1:1). Pd catalyst (2.5 times the weight of starting material) was added and the reaction was stirred in a high pressure reactor (60 psi H_2). The reaction progress was monitored to avoid undesired side products formation. Upon completion, the reaction was filtered and washed with *t*-BuOH and H_2O. The filtrates were concentrated *in vacuo.* [b]

[a]Reaction times and type of catalyst are indicated for each synthesis.

[b]Upon completion of hydrogenolysis, prior to filtration, the crude mixtures of compounds containing GlcNAc or GalNAc were treated with thiourea (10 equiv.).

Module J: Purification

The final compounds after global deprotection were purified by **Method B_1 or Method C_2** followed by **Method A_1** and analyzed using analytical HPLC (Agilent 1200 Series spectrometer, **Method C_1**).

- **Method A_1:** Sephadex® LH-20 column with H_20:MeOH (1:1) as eluent, isocratic.
- **Method B_1:** (Manual reverse phase C_{18} silica gel column chromatography): H_2O (10 mL), 5% MeOH (10 mL), 7.5% MeOH (10 mL), 10% MeOH (10 mL), 15% MeOH (10 mL), 20% MeOH (10 mL).
- **Method C_1:** (Hypercarb column, 150 x 4.60 mm) flow rate of 1.0 mL / min. 0 to 70% of B in 30 min (A = 0.01 M NH_4HCO_3, B = ACN); ELSD Detector: 45 °C
- **Method C_2:** (Hypercarb column, 150 x 10 mm) flow rate of 3.5 mL / min. 0 to 70% of B in 30 min (A = 0.01 M NH_4HCO_3, B = ACN); ELSD Detector: 45 °C
- **Method C_3:** (Hypercarb column, 150 x 10 mm) flow rate of 3.5 mL / min. 0 to 60% of B in 40 min (A = 0.01 M NH_4HCO_3, B = ACN); ELSD Detector: 45 °C

Module K: Ion exchange

The final purified compounds were passed through an Amberlite resin-Na^+ bed (2 cm diameter x 10 cm length, pre-swollen in water, eluent system: water).

9 Glycan backbones obtained by AGA

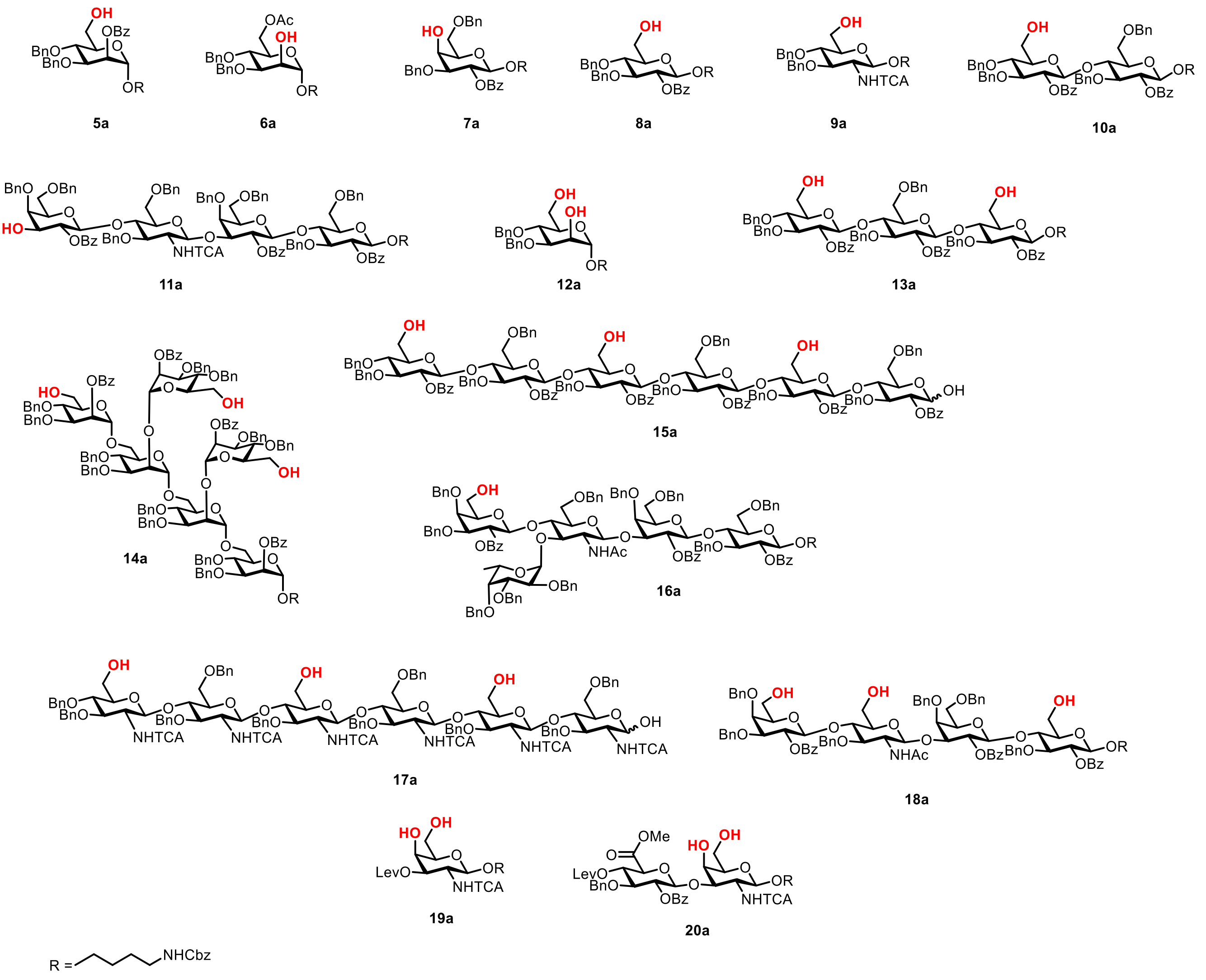

Figure S1. Glycan backbones synthesized on resin using AGA. The structures are reported after microcleavage from the solid support, performed to confirm the success of AGA. Sulfation sites are highlighted in red.

10 Oligosaccharides synthesis

10.1 Synthesis of 5

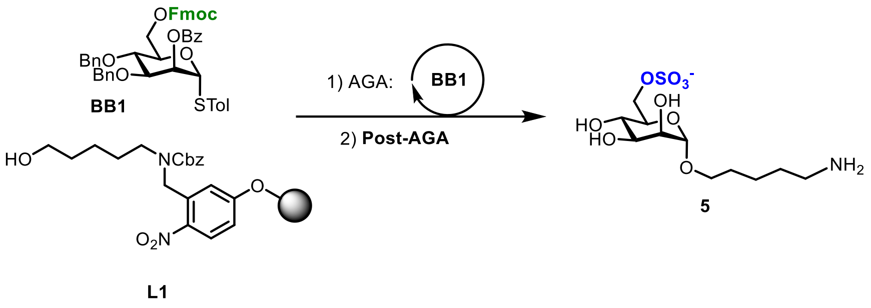

Step		Modules	Notes
AGA		**A**	
	**BB1**	**B, C1, D, E1**	**C1:** (**BB1**, -20 °C for 5 min, 0 °C for 20 min)
Post-AGA	**Sulfation**	**F**	
	**Hydrolysis**	**G**	**G:** (12 h)
	**Hydrogenolysis**	**I**	**I:** 10% Pd/C (12 h)
	**Purification**	**J(B1), J(A1), K**	

Compound **5** was obtained as a white solid (3.2 mg, 70% overall yield).

Analytical data for **5**: ^{1}H NMR (400 MHz, D_2O) δ 4.81 (d, *J* = 1.8 Hz, 1H), 4.34 (dd, *J* = 11.3, 2.1 Hz, 1H), 4.12 (dd, *J* = 11.3, 7.0 Hz, 1H), 3.89 (dd, *J* = 3.4, 1.7 Hz, 1H), 3.82 (m, 1H), 3.78 – 3.69 (m, 2H), 3.66 – 3.52 (m, 2H), 2.95 (t, *J* = 7.6 Hz, 2H), 1.64 (m, 4H), 1.49 – 1.35 (m, 2H); ^{13}C NMR (101 MHz, D_2O) δ 99.7, 70.8, 70.4, 69.8, 67.5, 66.4, 39.3, 27.8, 26.3, 22.2; HRMS (QToF): Calcd for $C_{11}H_{22}NO_9S$ $[M]^-$ 344.1021; found 344.1028.

^{1}H NMR of 5 (400 MHz, D_2O)

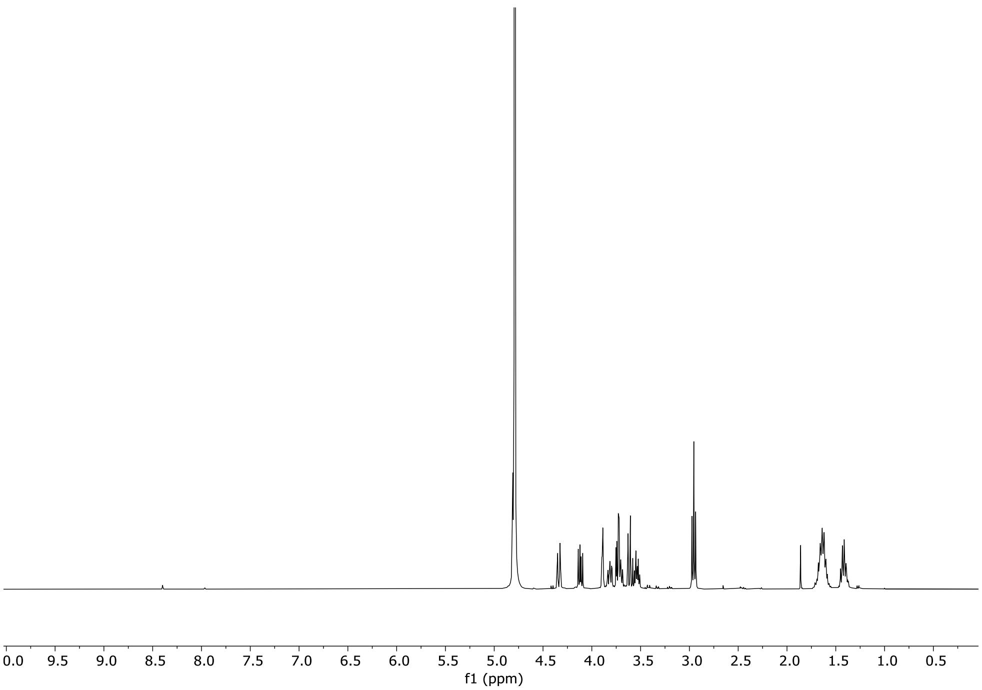

^{13}C NMR of 5 (101 MHz, D_2O)

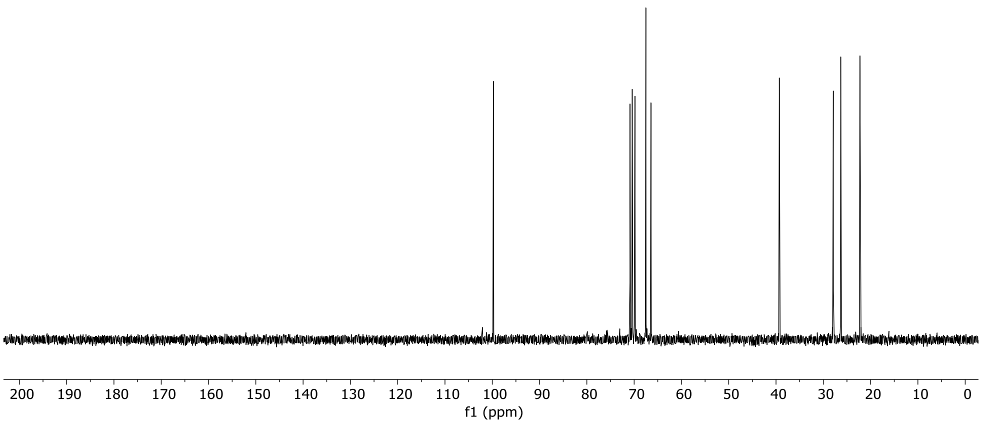

HSQC NMR of 5 (D_2O)

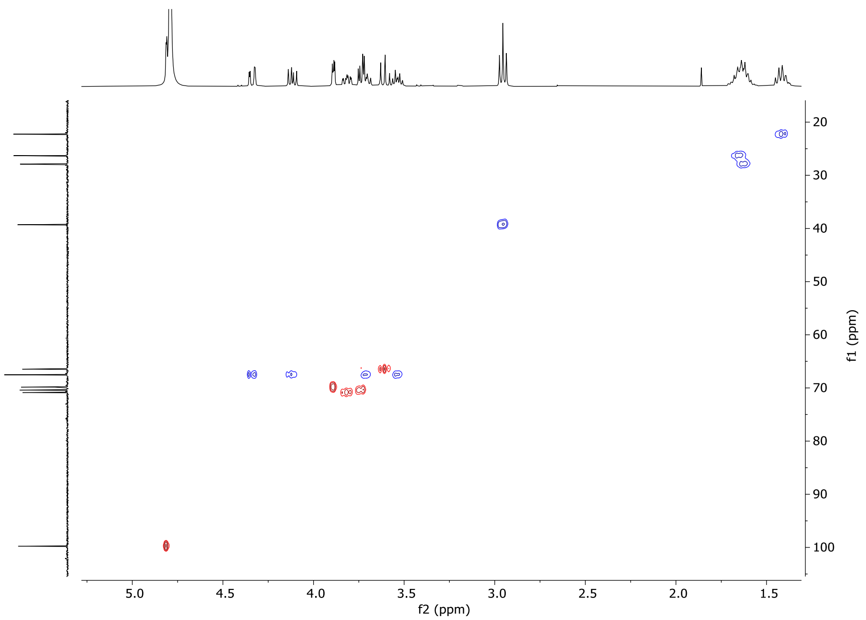

RP-HPLC of 5 (ELSD trace, Method C_1, t_R= 12.82 min)

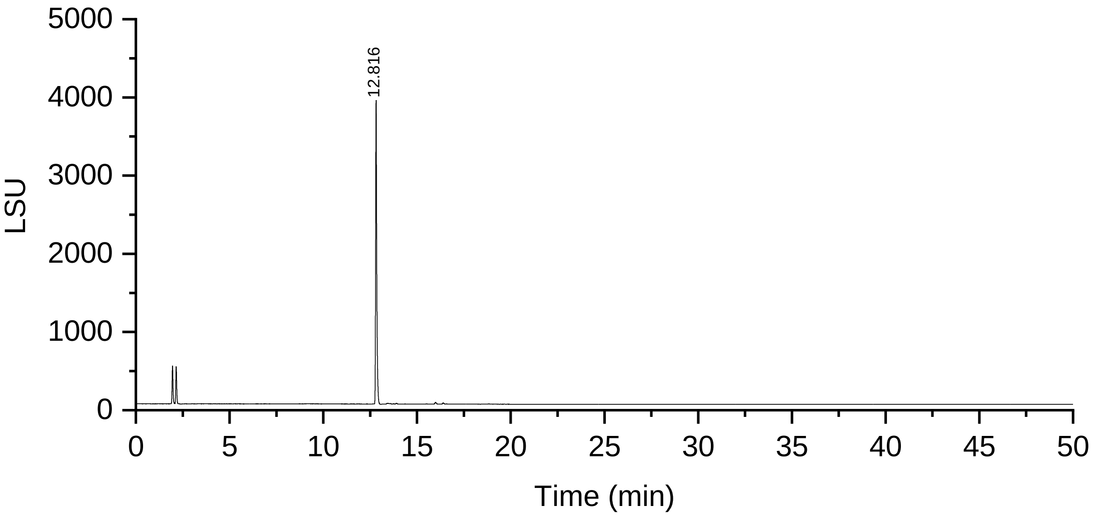

10.2 Synthesis of 6

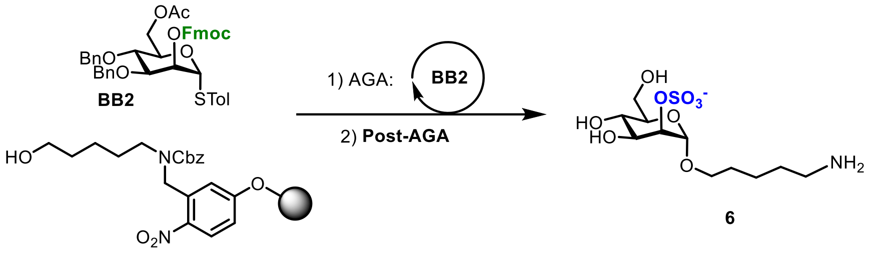

L1

Step		Modules	Notes
AGA		**A**	
	**BB2**	**B, C1, D, E1**	**C1:** (**BB2**, -20 °C for 5 min, 0 °C for 20 min)
Post-AGA	**Sulfation**	**F**	
	**Hydrolysis**	**G**	**G:** (12 h)
	**Hydrogenolysis**	**I**	**I:** 10% Pd/C (12 h)
	**Purification**	**J(B1), J(A1), K**	

Compound **6** was obtained as a yellowish solid (1.4 mg, 30% overall yield).

Analytical data for **6**: ^{1}H NMR (400 MHz, D_2O) δ 5.10 (d, *J* = 1.8 Hz, 1H), 4.47 (dd, *J* = 3.6, 1.7 Hz, 1H), 3.92 – 3.79 (m, 2H), 3.76 – 3.67 (m, 2H), 3.65 – 3.51 (m, 3H), 3.01 – 2.92 (m, 2H), 1.64 (m, 4H), 1.50 – 1.36 (m, 2H); ^{13}C NMR (101 MHz, D_2O) δ 97.1, 76.9, 72.6, 69.0, 67.5, 66.5, 60.6, 39.2, 27.7, 26.4, 22.2; HRMS (QToF): Calcd for $C_{11}H_{22}NO_9S$ $[M]^-$ 344.1021; found 344.1013

^{1}H NMR of 6 (400 MHz, D_2O)

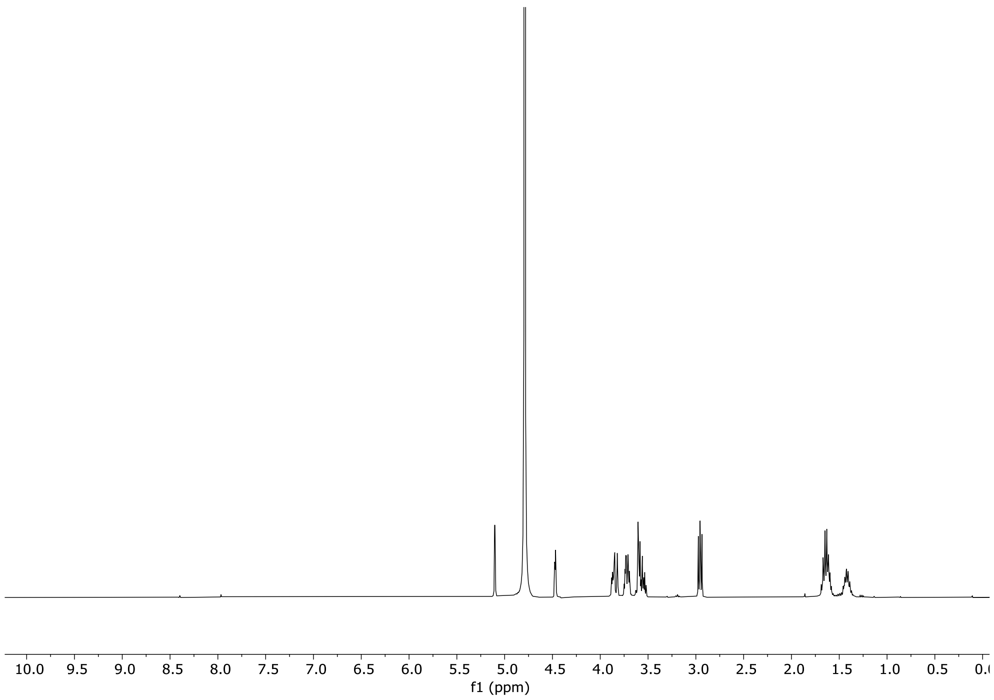

^{13}C NMR of 6 (101 MHz, D_2O)

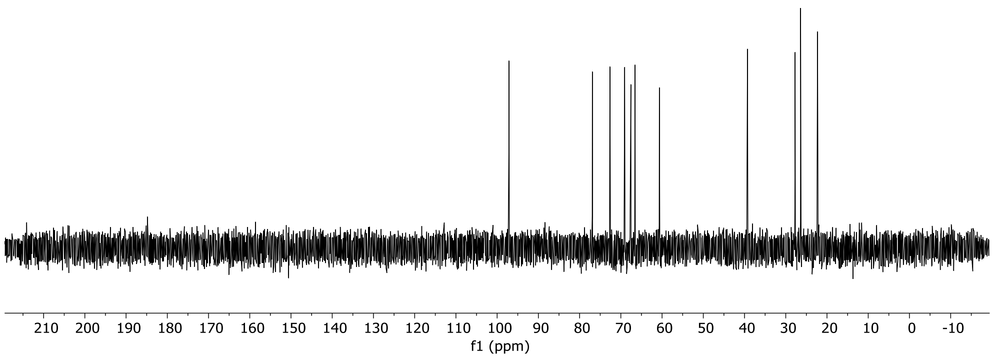

HSQC NMR of 6 (D_2O)

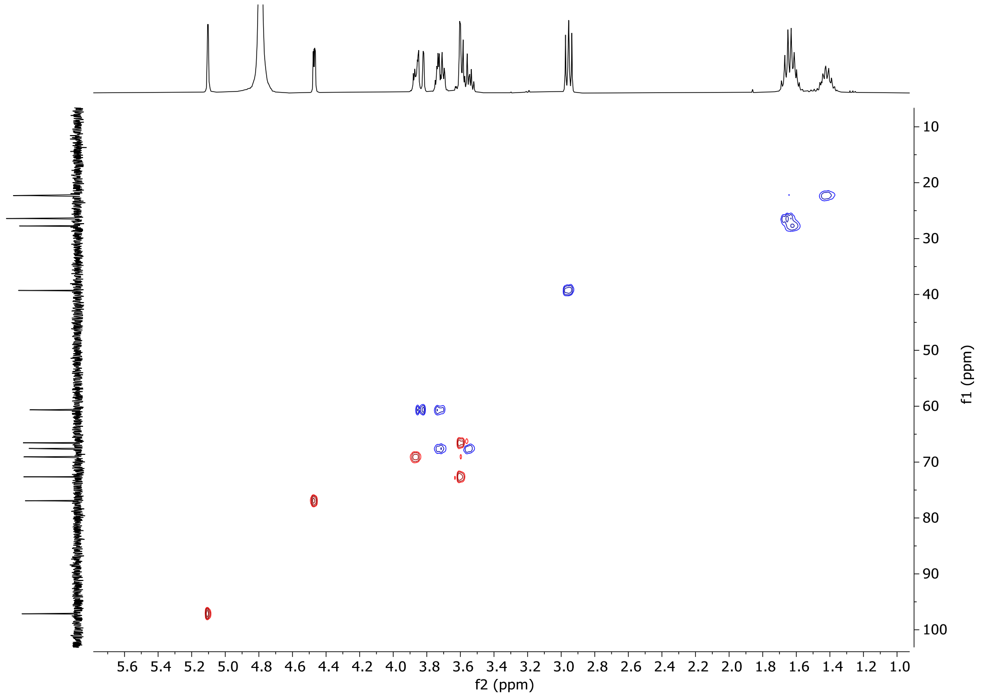

RP-HPLC of 6 (ELSD trace, Method C_1, t_R= 12.39 min)

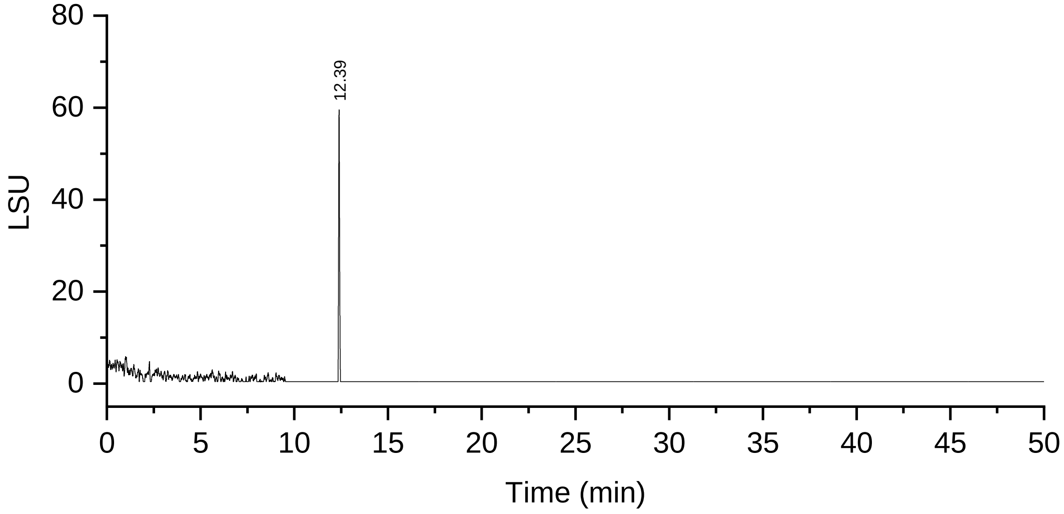

10.3 Synthesis of 7

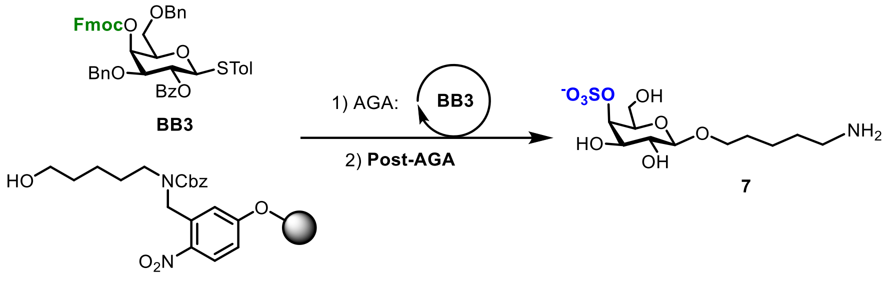

Step		Modules	Notes
AGA		A	
	BB3	B, C1, D, E1	**C1:** (**BB3**, -20 °C for 5 min, 0 °C for 20 min)
Post-AGA	**Sulfation**	F	
	**Hydrolysis**	G	**G:** (12 h)
	**Hydrogenolysis**	I	**I:** 10% Pd/C (12 h)
	**Purification**	J(B1), J(A1), K	

Compound **7** was obtained as a white solid (1.4 mg, 30% overall yield).

Analytical data for **7**: ^{1}H NMR (400 MHz, D_2O) δ 4.61 (d, *J* = 3.3 Hz, 1H), 4.39 (dd, *J* = 7.9, 3.3 Hz, 1H), 3.90 (dt, *J* = 10.0, 6.5 Hz, 1H), 3.82 – 3.60 (m, 7H), 3.51 – 3.44 (m, 1H), 2.96 (dd, *J* = 9.0, 6.0 Hz, 2H), 1.68 – 1.58 (m, 4H), 1.45 – 1.39 (m, 2H); ^{13}C NMR (101 MHz, D_2O) δ 102.5, 76.4, 74.2, 71.7, 70.7, 69.9, 60.8, 39.2, 28.0, 26.3, 22.0; HRMS (QToF): Calcd for $C_{11}H_{22}NO_9S$ $[M]^-$ 344.1021; found 344.1020.

^{1}H NMR of 7 (400 MHz, D_2O)

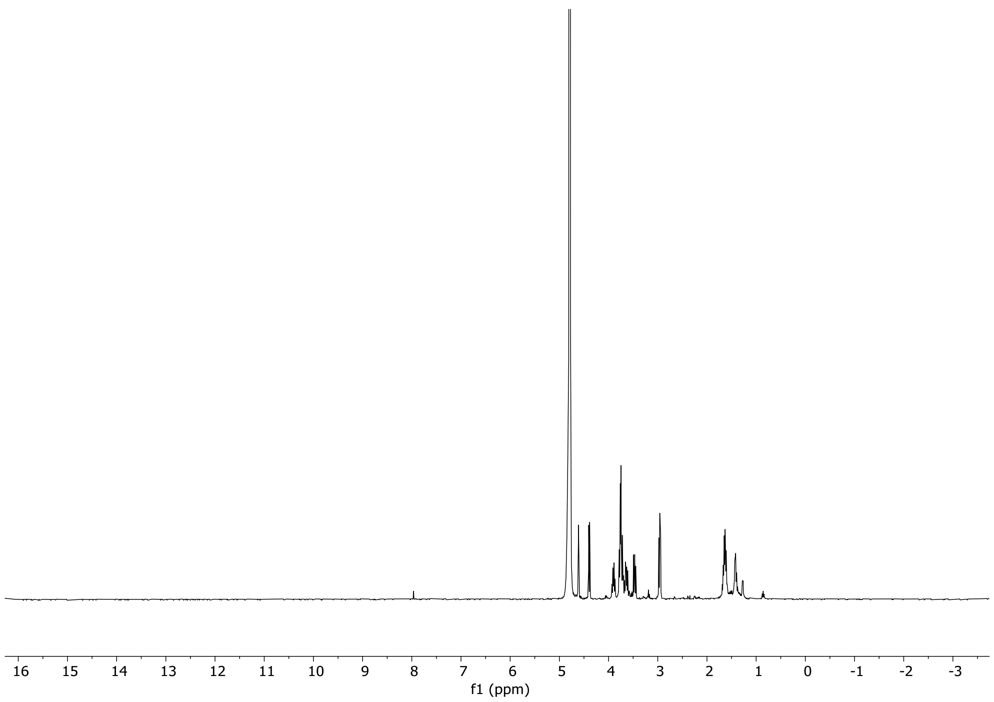

^{13}C NMR of 7 (101 MHz, D_2O)

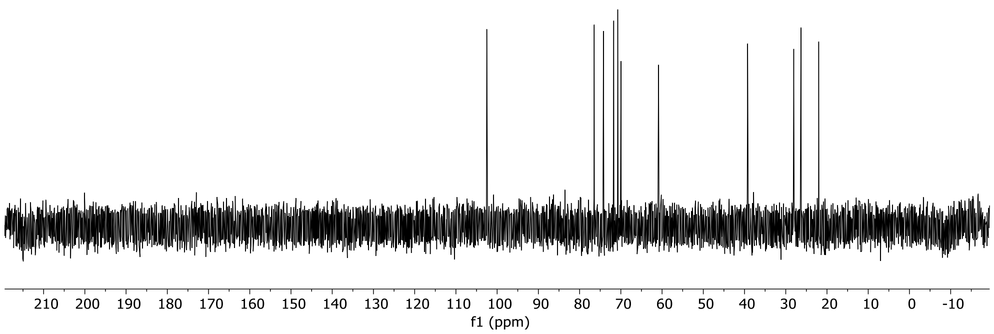

HSQC NMR of 7 (D_2O)

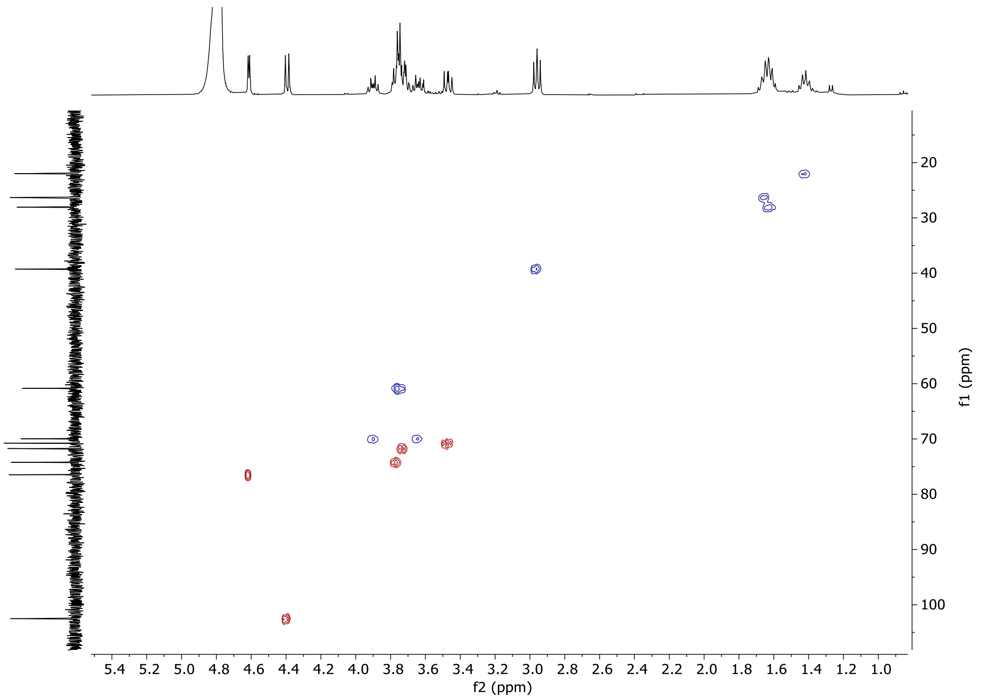

RP-HPLC of 7 (ELSD trace, Method C_1, t_R= 13.52 min)

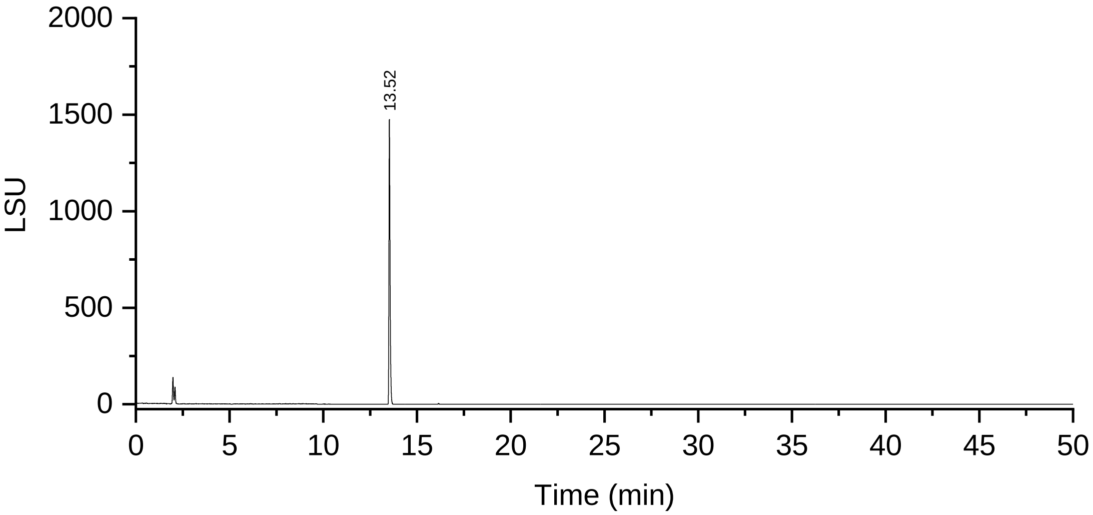

10.4 Synthesis of 8

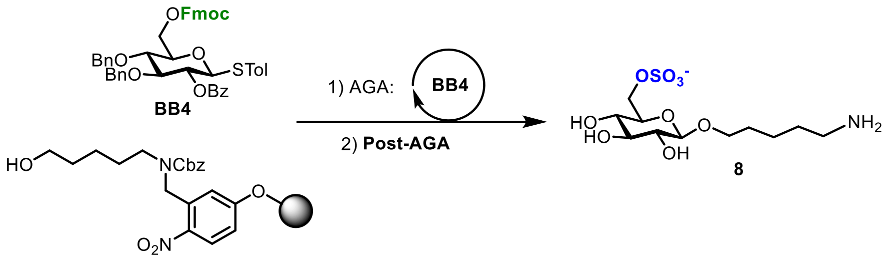

L1

Step		Modules	Notes
AGA		**A**	
	**BB4**	**B, C1, D, E1**	**C1:** (**BB5**, -20 °C for 5 min, 0 °C for 20 min)
Post-AGA	**Sulfation**	**F**	
	**Hydrolysis**	**G**	**G:** (12 h)
	**Hydrogenolysis**	**I**	**I:** 10% Pd/C (12 h)
	**Purification**	**J(B1), J(A1), K**	

Compound **8** was obtained as a white solid (3 mg, 65% overall yield).

Analytical data for **8**: ^{1}H NMR (400 MHz, D_2O) δ 4.42 (d, *J* = 8.0 Hz, 1H), 4.29 (dd, *J* = 11.2, 2.1 Hz, 1H), 4.14 (dd, *J* = 11.2, 5.6 Hz, 1H), 3.86 (dt, *J* = 10.2, 6.5 Hz, 1H), 3.75 – 3.58 (m, 2H), 3.51 – 3.35 (m, 2H), 3.23 (dd, *J* = 9.0, 8.0 Hz, 1H), 2.95 (t, *J* = 7.5 Hz, 2H), 1.75 – 1.54 (m, 4H), 1.51 – 1.33 (m, 2H).; ^{13}C NMR (101 MHz, D_2O) δ 102.9, 76.2, 74.2, 73.6, 70.9, 69.8, 67.6, 39.9, 28.8, 26.9, 22.5; HRMS (QToF): Calcd for $C_{11}H_{22}NO_9S$ $[M]^-$ 344.1021; found 344.1016.

^{1}H NMR of 8 (400 MHz, D_2O)

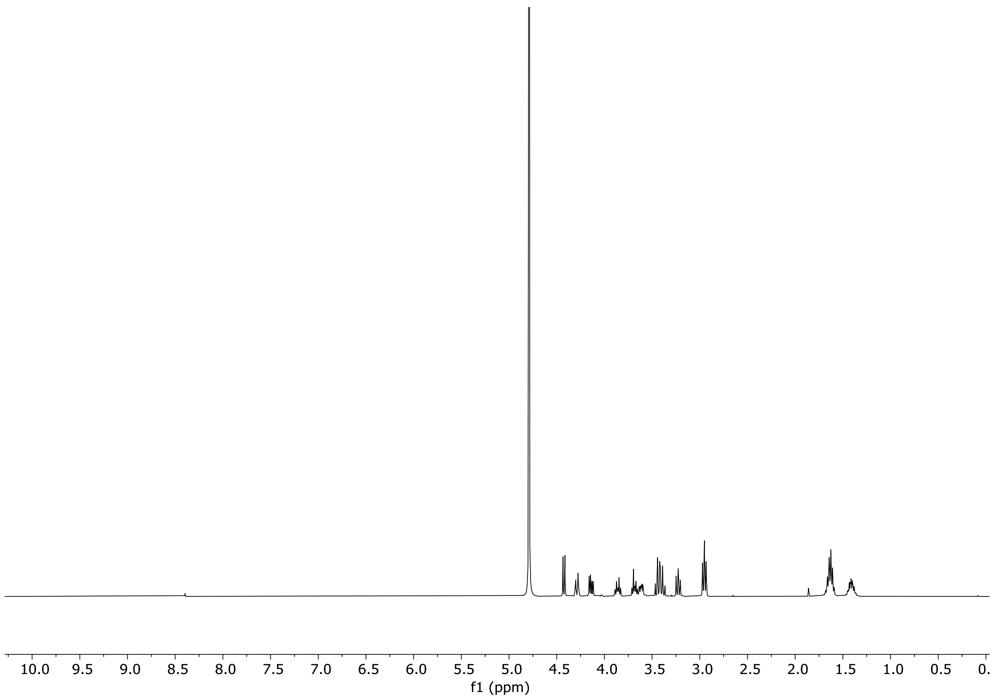

^{13}C NMR of 8 (101 MHz, D_2O)

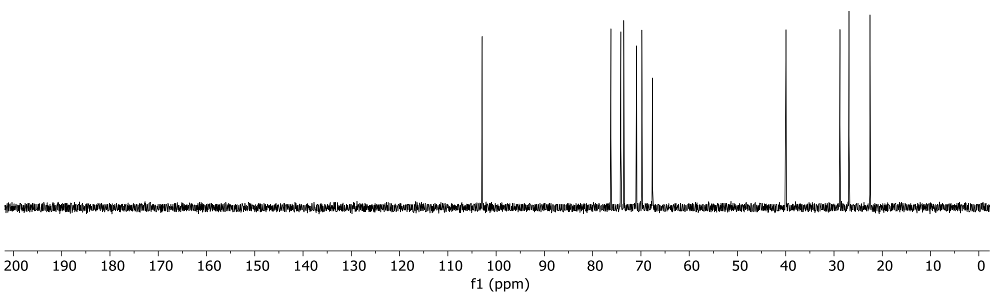

HSQC NMR of 8 (D_2O)

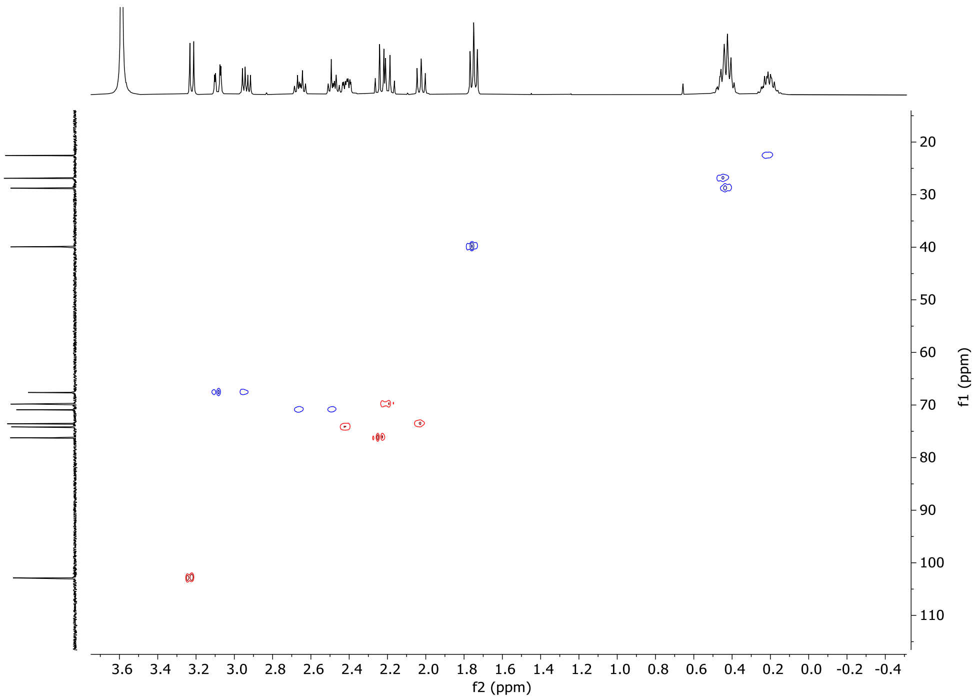

RP-HPLC of 8 (ELSD trace, Method C1, t_R= 12.38 min)

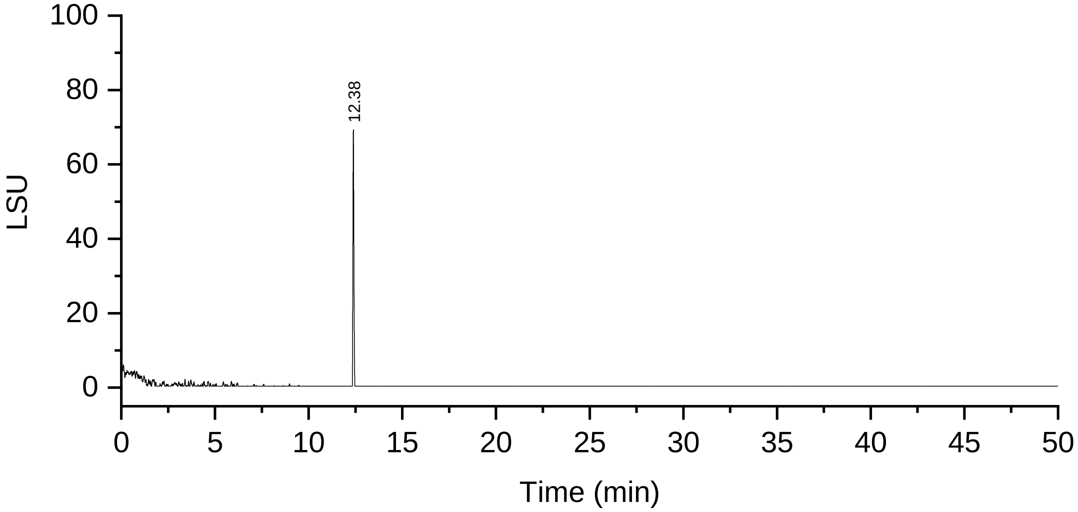

10.5 Synthesis of 9

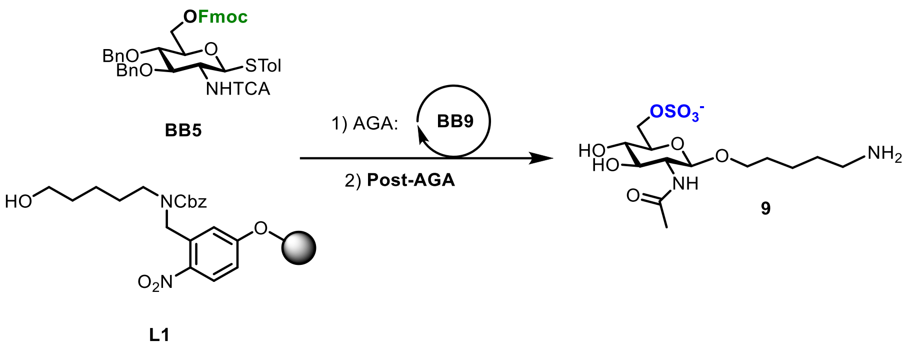

Step	Modules		Notes
AGA		**A**	
	**BB5**	**B, C1, D, E1**	**C1:** (**BB5**, -20 °C for 5 min, 0 °C for 20 min)
Post-AGA	**Sulfation**	**F**	
	**Hydrogenolysis**	**I1**[a]	**I1:** 10-20% $Pd(OH)_2/C$ (12 h)
	**Purification**	**J(B1), J(A1), K**	

[a] treated with thiourea upon completion of hydrogenolysis

Compound **9** was obtained as a white solid (2 mg, 38% overall yield).

Analytical data for **9**: ^{1}H NMR (600 MHz, D_2O) δ 4.54 (d, *J* = 8.5 Hz, 1H), 4.37 (dd, *J* = 11.3, 2.1 Hz, 1H), 4.22 (dd, *J* = 11.2, 5.8 Hz, 1H), 3.89 (dt, *J* = 10.3, 6.2 Hz, 1H), 3.74 – 3.60 (m, 4H), 3.60 – 3.47 (m, 3H), 3.01 (t, *J* = 7.6 Hz, 2H), 2.05 (s, 3H), 1.69 (m, 2H), 1.63 (m, 2H), 1.43 (m, 2H); ^{13}C NMR (151 MHz, D_2O) δ 174.5, 101.2, 73.7, 73.6, 70.2, 69.6, 67.1, 55.5, 39.3, 28.1, 26.2, 22.1, 22.0; HRMS (QToF)**:** Calcd for $C_{13}H_{25}N_2O_9S$ $[M]^-$ 385.1286; found 385.1288.

^{1}H NMR of 9 (600 MHz, D_2O)

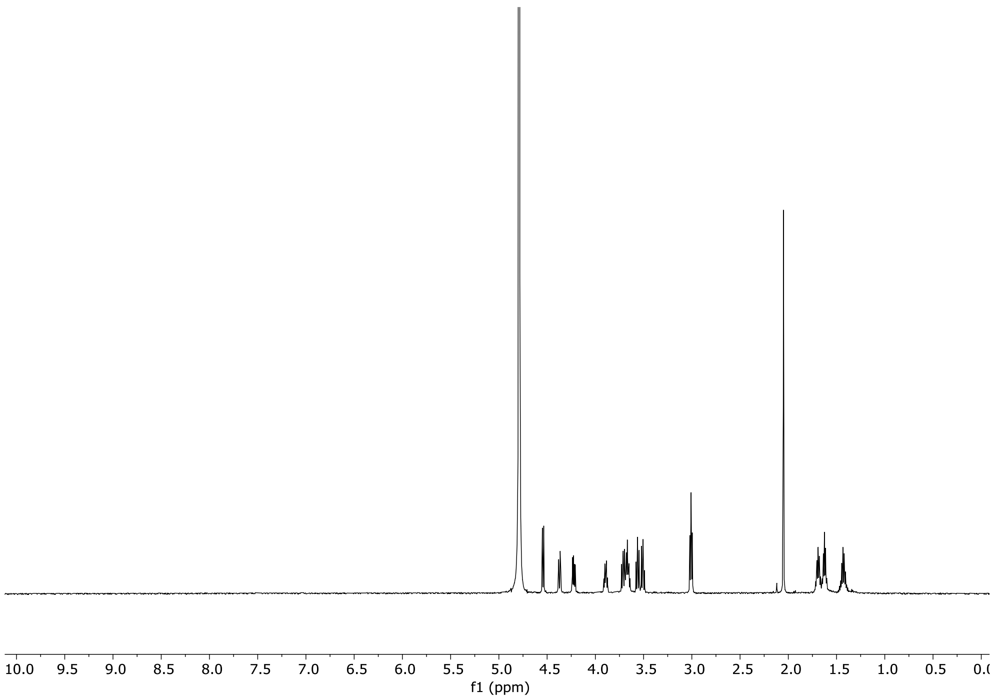

^{13}C NMR of 9 (151 MHz, D_2O)

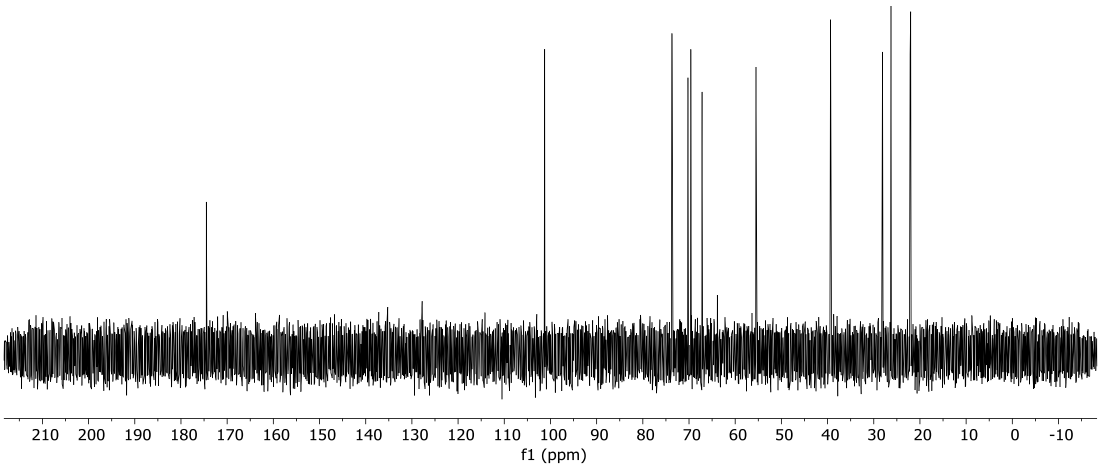

HSQC NMR of 9 (D_2O)

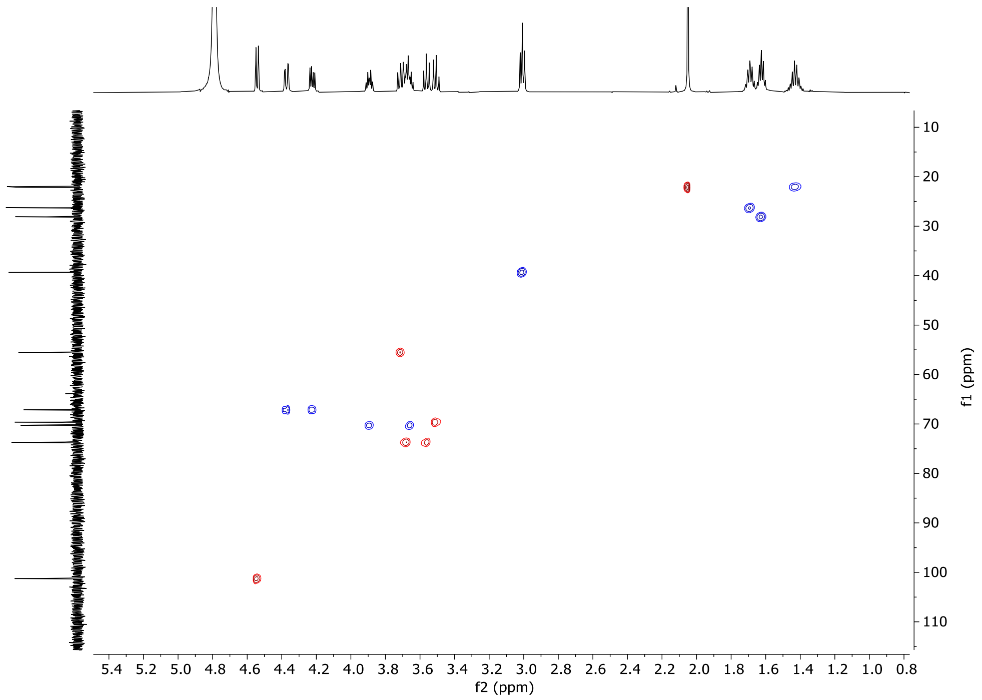

RP-HPLC of 9 (ELSD trace, Method C1, t_R= 13.3 min)

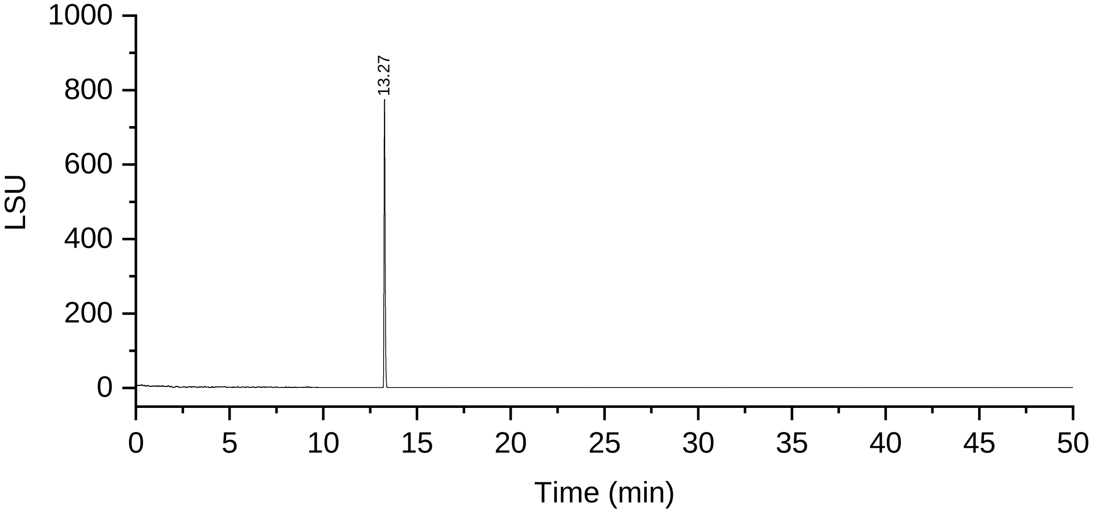

10.6 Synthesis of 10

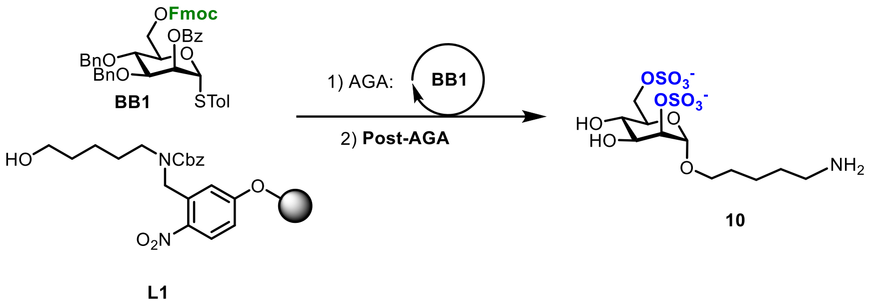

Step		Modules	Notes
AGA		**A**	
	**BB1**	**B, C1, D, E1**	**C1:** (**BB1**, **-**20 °C for 5 min, 0 °C for 20 min)
Post-AGA	**Hydrolysis**	**G**	**G:** (12 h)
	**Sulfation**	**F**	
	**Hydrogenolysis**	**I**	**I:** 10% Pd/C (12 h)
	**Purification**	**J(B1), J(A1), K**	

Compound **10** was obtained as yellowish solid (2.7 mg, 49% overall yield).

Analytical data for **10**: ^{1}H NMR (400 MHz, D_2O) δ 5.09 (d, *J* = 1.7 Hz, 1H), 4.47 (dd, *J* = 3.5, 1.7 Hz, 1H), 4.34 (dd, *J* = 11.3, 2.1 Hz, 1H), 4.13 (dd, *J* = 11.4, 7.0 Hz, 1H), 3.92 – 3.82 (m, 2H), 3.73 (m, 1H), 3.65 – 3.53 (m, 2H), 2.96 (t, *J* = 7.6 Hz, 2H), 1.64 (m, 4H), 1.42 (m, 2H); ^{13}C NMR (101 MHz, D_2O) δ 97.3, 76.7, 70.8, 69.0, 67.7, 67.4, 66.4, 39.3, 27.7, 26.2, 22.2; HRMS (QToF): Calcd for $C_{11}H_{22}NO_{12}S_2$ $[M+H]^-$ 424.0589; found 424.0598.

^{1}H NMR of 10 (400 MHz, D_2O)

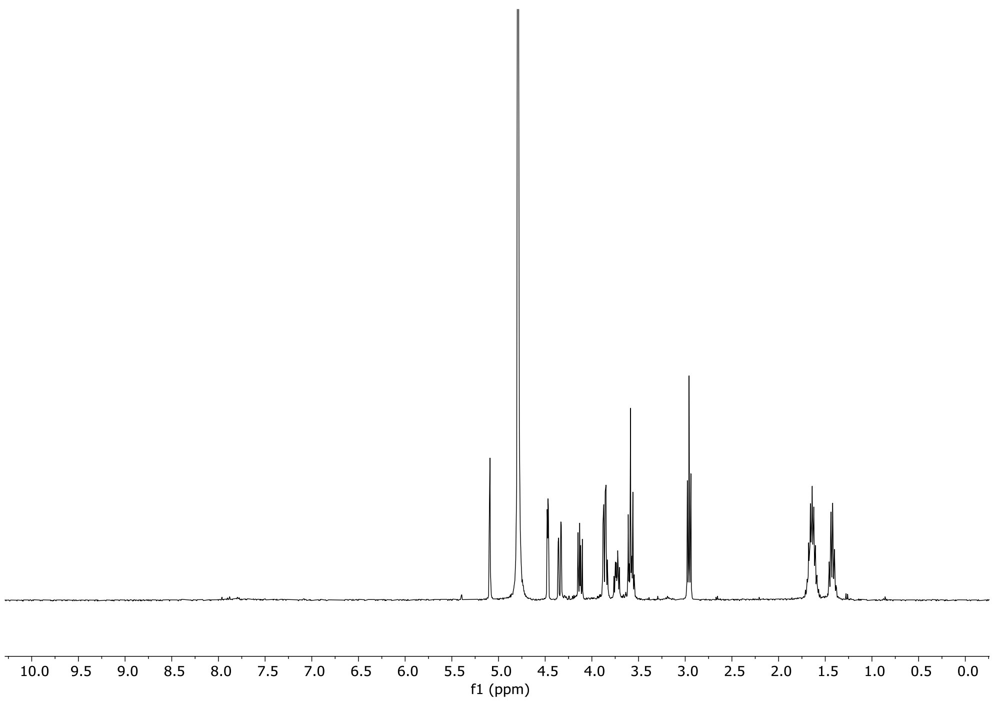

^{13}C NMR of 10 (101 MHz, D_2O)

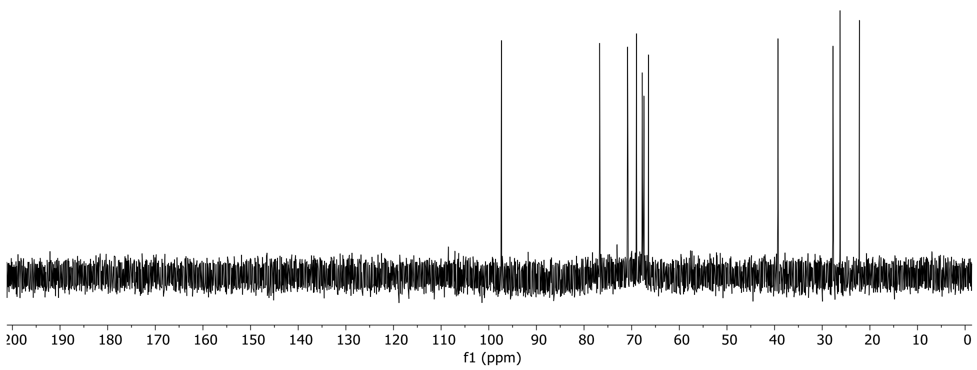

HSQC NMR of 10 (D_2O)

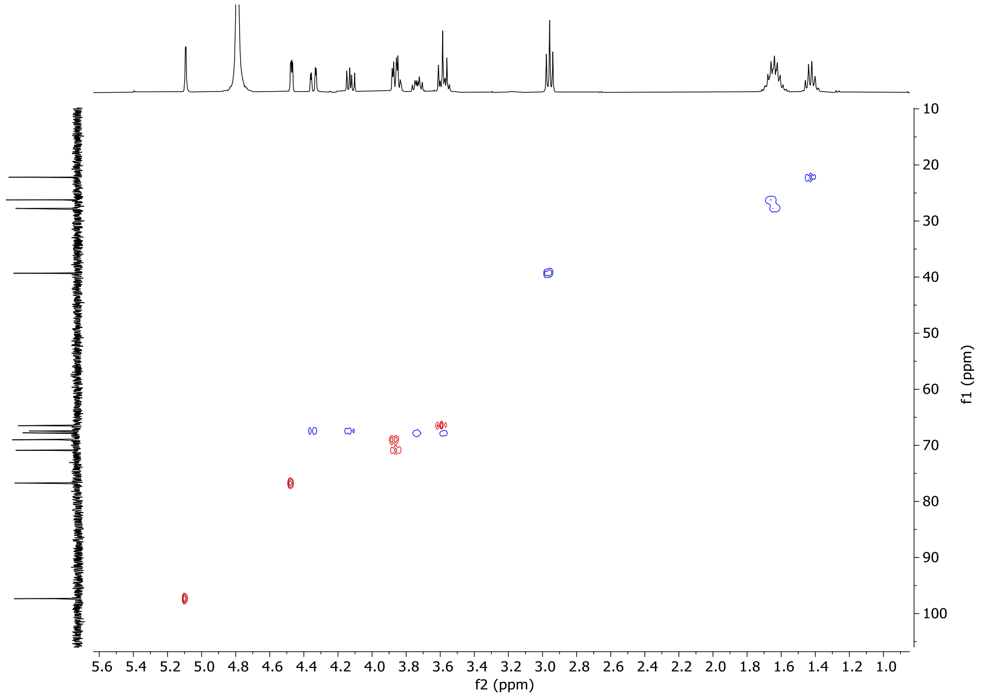

RP-HPLC of 10 (ELSD trace, Method C_1, t_R= 13.0 min)

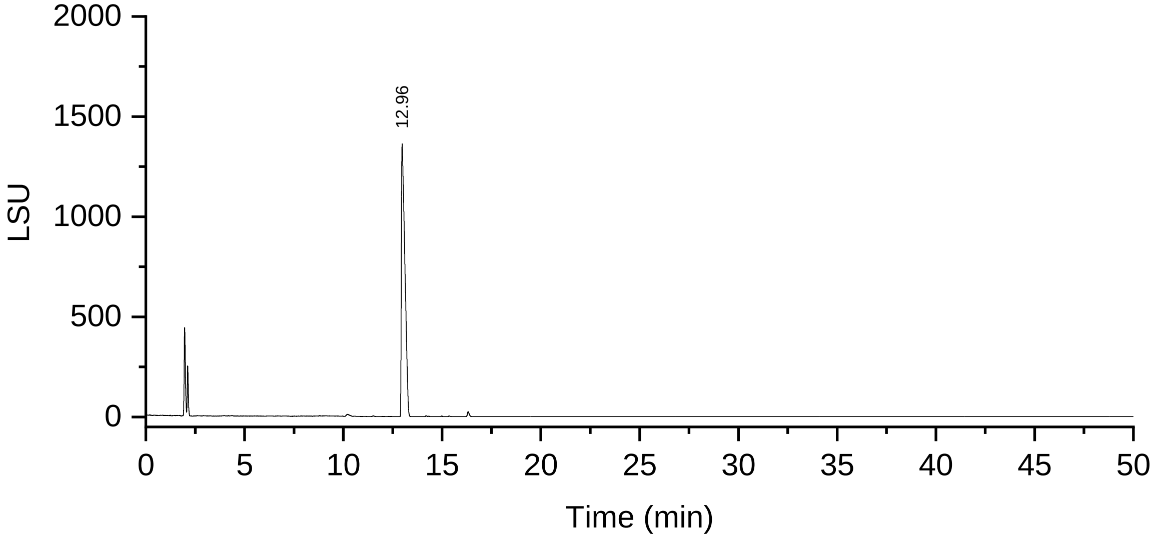

10.7 Synthesis of 11

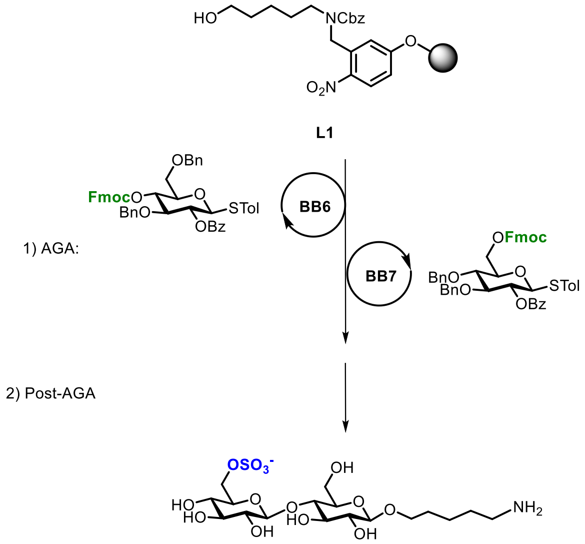

11

Step		Modules	Notes
AGA		**A**	
	**BB6**	**B, C1, D, E1**	**C1:** (**BB6**, -20 °C for 5 min, 0 °C for 20 min)
	**BB7**	**B, C1, D, E1**	**C1:** (**BB7**, -20 °C for 5 min, 0 °C for 20 min)
Post-AGA	**Sulfation**	**F**	
	**Hydrolysis**	**G**	**G:** (12 h)
	**Hydrogenolysis**	**I**	**I:** 10% Pd/C (12 h)
	**Purification**	**J(B1), J(A1), K**	

Compound **11** was obtained as a white solid (1.7 mg, 25% overall yield).

Analytical data for **11**: ^{1}H NMR (400 MHz, D_2O) δ 4.48 (d, *J* = 7.9 Hz, 1H), 4.44 (d, *J* = 8.1 Hz, 1H), 4.30 (dd, *J* = 11.2, 2.2 Hz, 1H), 4.16 (dd, *J* = 11.2, 5.7 Hz, 1H), 3.90 (dt, *J* = 13.0, 9.4 Hz, 2H), 3.78 – 3.53 (m, 6H), 3.50 – 3.39 (m, 2H), 3.27 (td, *J* = 9.1, 7.9 Hz, 2H), 2.99 – 2.91 (m, 2H), 1.70 – 1.58 (m, 4H), 1.42 (q, *J* = 8.2 Hz, 2H); ^{13}C NMR (101 MHz, D_2O) δ 102.5, 101.8, 79.3, 75.2, 74.6, 74.2, 73.6, 72.9, 72.7, 69.9, 69.0, 66.9, 60.0, 39.2, 28.0, 26.3, 22.0; HRMS (QToF): Calcd for $C_{17}H_{32}N_1O_{14}S$ $[M]^-$ 506.1549; found 506.1537.

^{1}H NMR of 11 (400 MHz, D_2O)

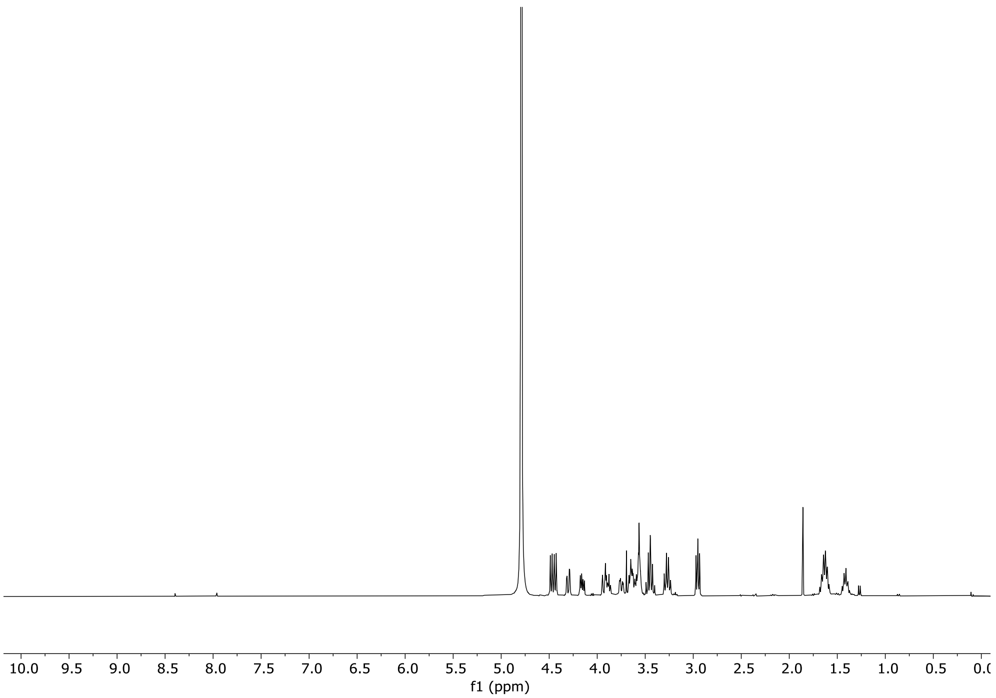

^{13}C NMR of 11 (101 MHz, D_2O)

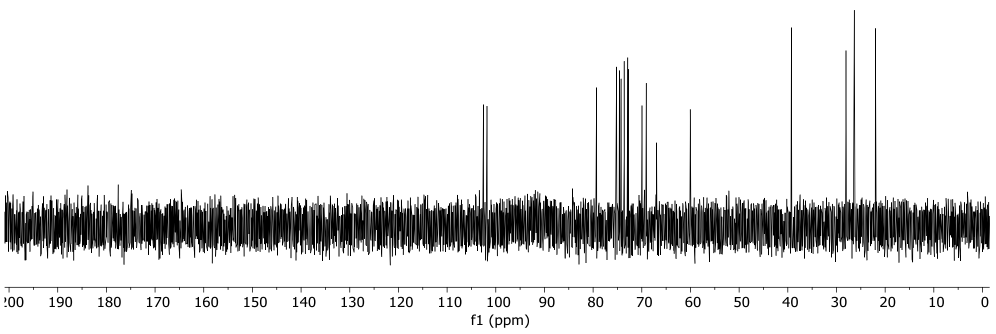

HSQC NMR of 11 (D_2O)

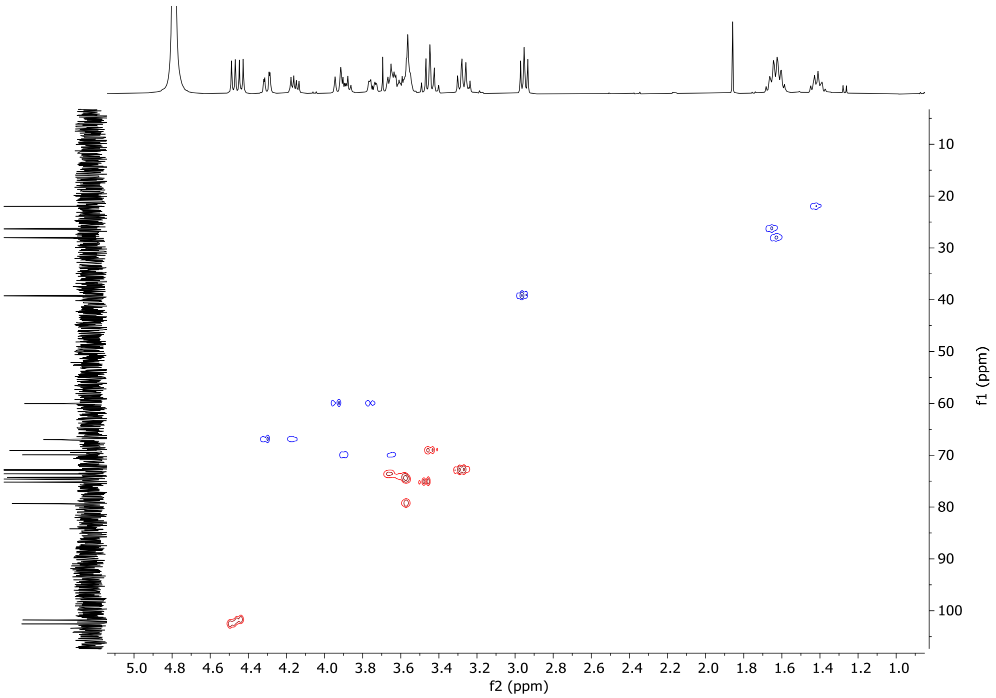

RP-HPLC of 11 (ELSD trace, Method C1, t_R= 17.0 min)

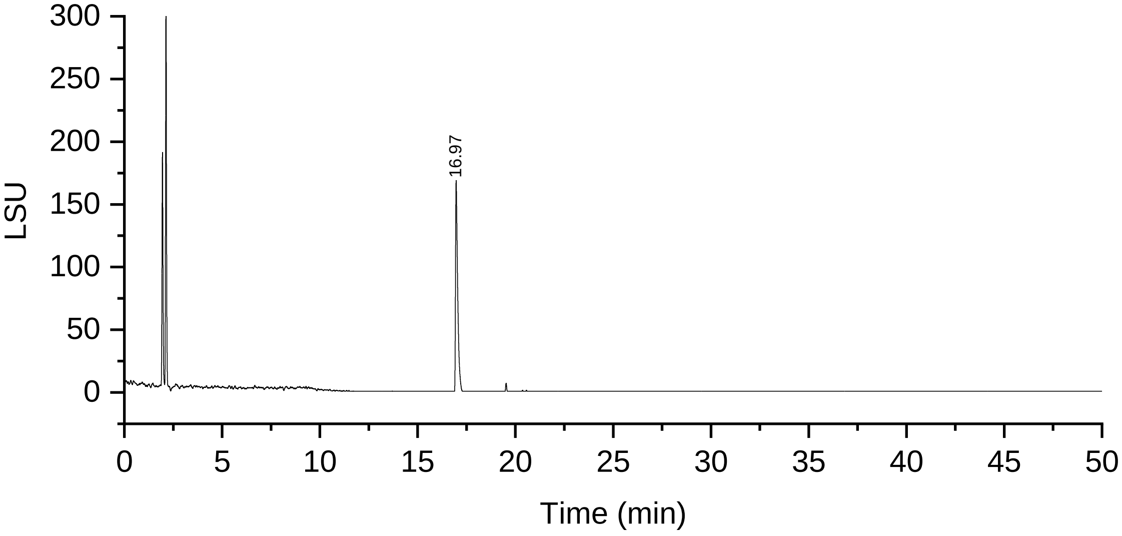

10.8 Synthesis of 12

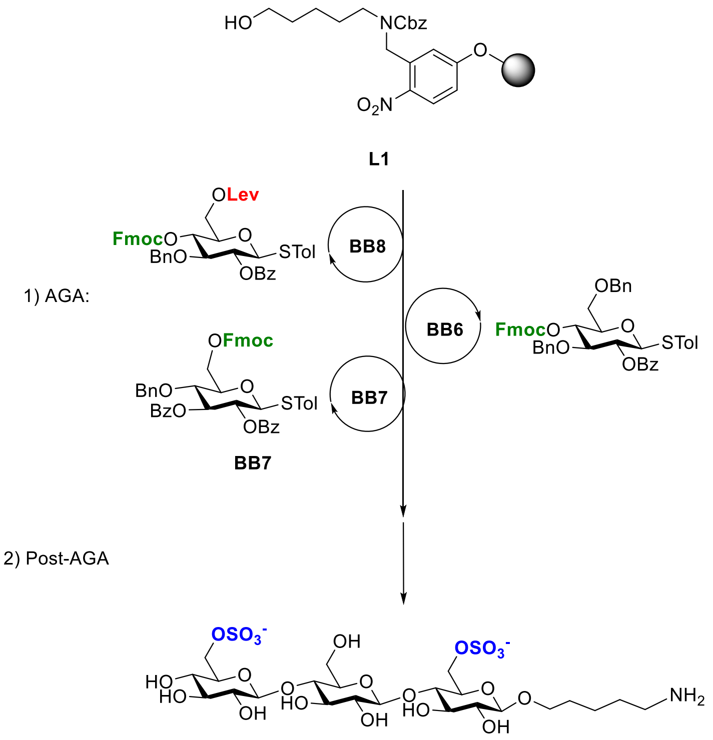

12

Step		Modules	Notes
AGA		**A**	
	**BB8**	**B, C1, D, E1**	**C1:** (**BB8**, -20 °C for 5 min, 0 °C for 20 min)
	**BB6**	**B, C1, D, E1**	**C1:** (**BB6**, -20 °C for 5 min, 0 °C for 20 min)
	**BB7**	**B, C1, D, E1, E2**	**C1:** (**BB7**, -20 °C for 5 min, 0 °C for 20 min)
Post-AGA	**Sulfation**	**F**	
	**Hydrolysis**	**G**	**G:** (24 h)
	**Hydrogenolysis**	**I**	**I:** 10% Pd/C (20 h)
	**Purification**	**J(B1), J(A1), K**	

Compound **12** was obtained as a white solid (1.2 mg, 16% overall yield).

Analytical data for **12**: ^{1}H NMR (700 MHz, D_2O) δ 4.59 (d, *J* = 7.9 Hz, 1H), 4.51 (dd, *J* = 11.1, 8.0 Hz, 2H), 4.40 (d, *J* = 10.9 Hz, 1H), 4.36 (d, *J* = 11.1 Hz, 1H), 4.30 (dd, *J* = 11.2, 4.5 Hz, 1H), 4.22 (dd, *J* = 11.2, 5.4 Hz, 1H), 3.99 (d, *J* = 12.2 Hz, 1H), 3.92 (dd, *J* = 10.7, 6.2 Hz, 1H), 3.86 – 3.60 (m, 9H), 3.55 – 3.45 (m, 2H), 3.34 (p, *J* = 9.2 Hz, 3H), 3.01 (t, *J* = 7.5 Hz, 2H), 1.69 (dp, *J* = 14.3, 7.0 Hz, 4H), 1.47 (dq, *J* = 15.6, 7.0 Hz, 2H); ^{13}C NMR (176 MHz, D_2O) δ 102.7, 102.1, 101.8, 79.0, 77.4, 75.2, 74.7, 74.2, 74.0, 73.7, 73.0, 72.9, 72.8, 72.5, 70.3, 69.1, 67.0, 66.3, 59.9, 39.4, 28.2, 26.3, 22.0; HRMS (QToF): Calcd for $C_{23}H_{41}N_1O_{22}S_2$ $[M]^{2-}$ 373.5786; found 373.5791.

1H NMR of 12 (700 MHz, D_2O)

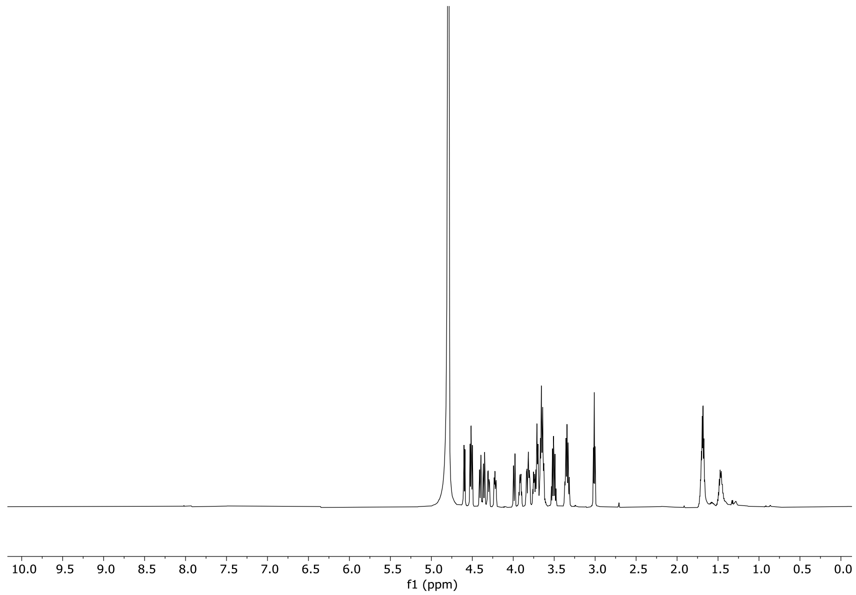

^{13}C NMR of 12 (176 MHz, D_2O)

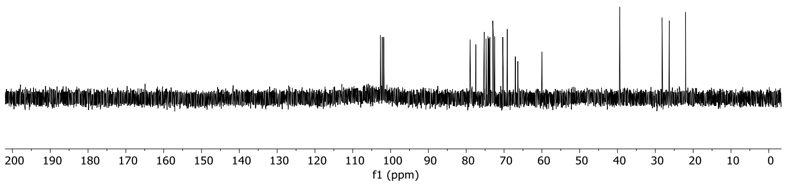

HSQC NMR of 12 (D_2O)

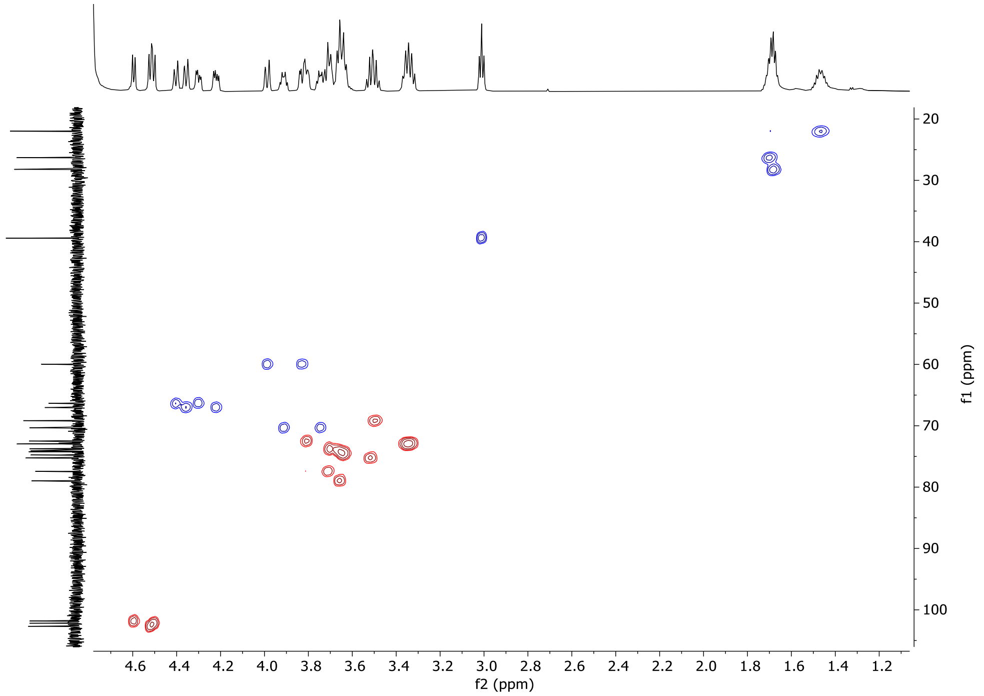

RP-HPLC of 12 (ELSD trace, Method C1, t_R= 19.3 min)

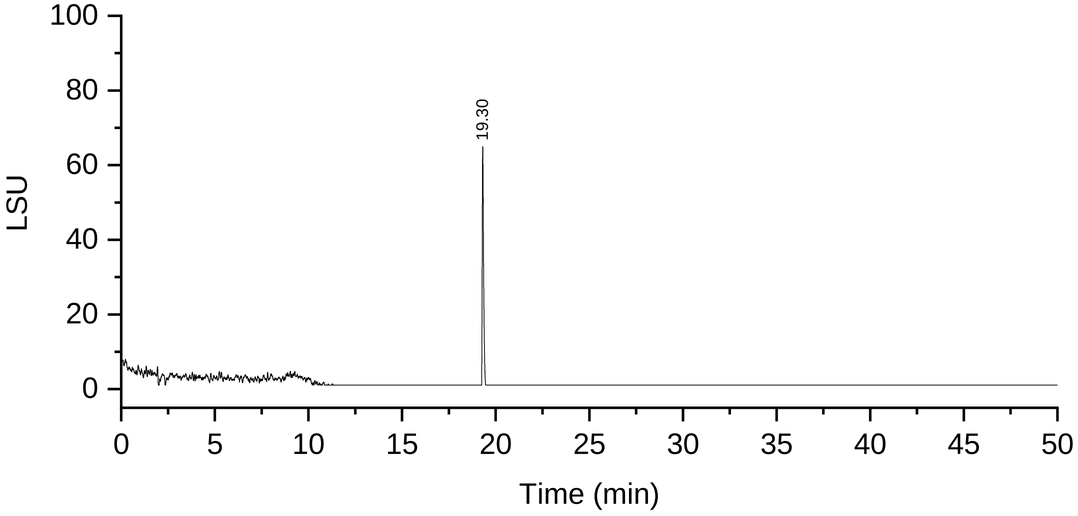

10.9 Synthesis of 13

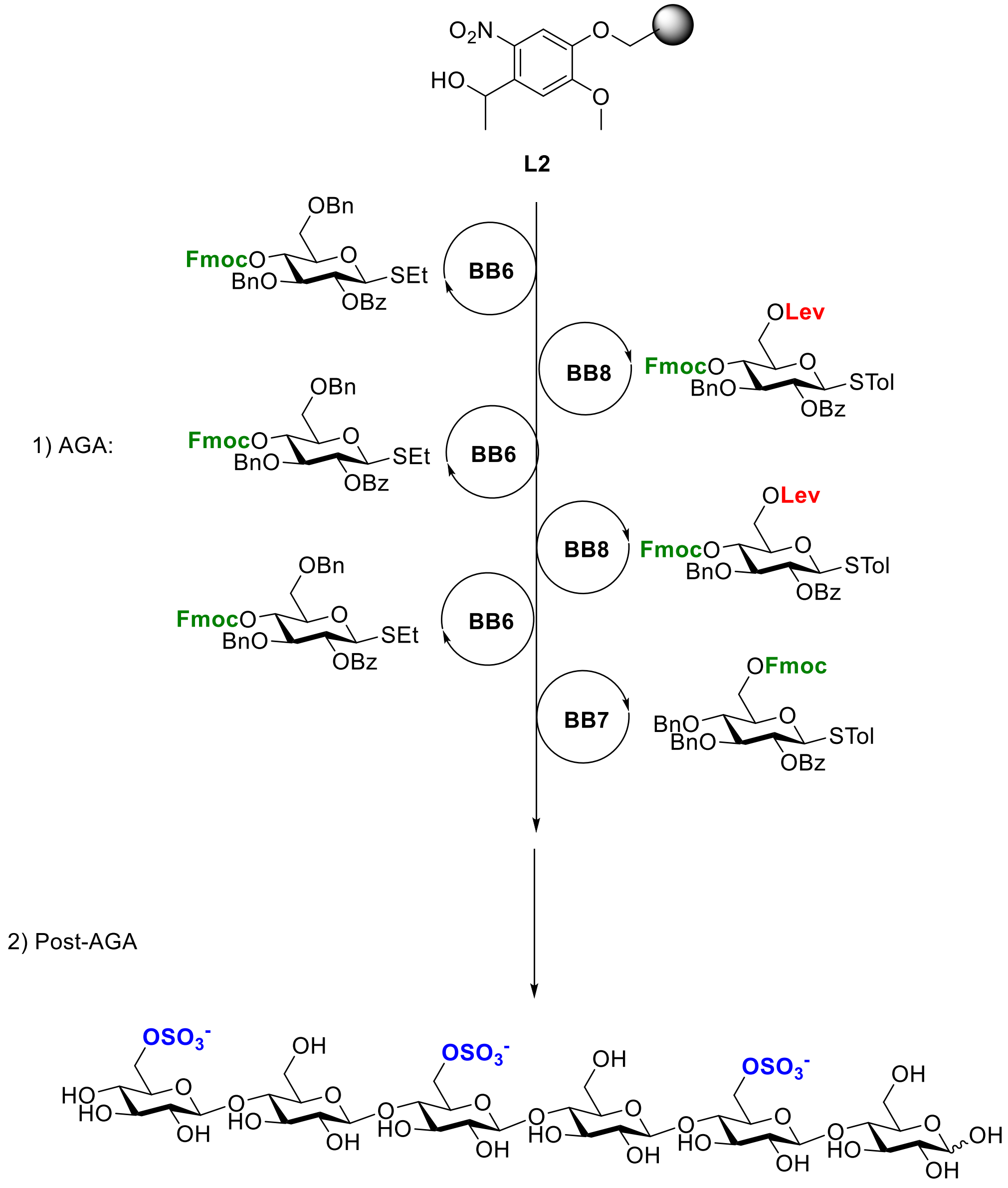

Step		Modules	Notes
AGA		**A**	
	**BB6**	**B, C1, D, E1**	**C1:** (**BB6**, -20 °C for 5 min, 0 °C for 20 min)
	**BB8**	**B, C1, D, E1**	**C1:** (**BB8**, -20 °C for 5 min, 0 °C for 20 min)
	**BB6**	**B, C1, D, E1**	**C1:** (**BB6**, -20 °C for 5 min, 0 °C for 20 min)
	**BB8**	**B, C1, D, E1**	**C1:** (**BB8**, -20 °C for 5 min, 0 °C for 20 min)
	**BB6**	**B, C1, D, E1**	**C1:** (**BB6**, -20 °C for 5 min, 0 °C for 20 min)
	**BB7**	**B, C1, D, E1, E2**	**C1:** (**BB7**, -20 °C for 5 min, 0 °C for 20 min)
Post-AGA	**Sulfation**	**F**	
	**Hydrolysis**	**G**	**G:** (72 h)
	**Hydrogenolysis**	**I**	**I:** 10% Pd/C (48 h)
	**Purification**	**J(B1), J(A1), K**	

Compound **13** was obtained as a white solid (10 mg, 60% overall yield).

Analytical data for **13**: ^{1}H NMR (700 MHz, D_2O) δ 5.17 (d, *J* = 3.8 Hz, 1H), 4.61 (d, *J* = 8.0 Hz, 1H), 4.56 – 4.45 (m, 5H), 4.39 – 4.24 (m, 5H), 4.17 (dd, *J* = 11.2, 5.3 Hz, 1H), 3.92 (dd, *J* = 16.0, 12.8 Hz, 4H), 3.83 – 3.73 (m, 6H), 3.73 – 3.67 (m, 2H), 3.67 – 3.52 (m, 10H), 3.50 – 3.40 (m, 3H), 3.37 – 3.21 (m, 4H); ^{13}C NMR (151 MHz, D_2O) δ 102.7, 102.4, 101.8, 101.7, 95.6, 79.2, 79.0, 78.9, 78.6, 77.1, 77.0, 75.1, 74.6, 74.1, 73.9, 73.9, 73.7, 73.6, 72.8, 72.8, 72.7, 72.7, 72.4, 71.2, 69.9, 69.0, 66.9, 66.1, 60.0, 59.8; HRMS (QToF): Calcd for $C_{36}H_{59}O_{40}S_3$ $[M]^{3-}$ 409.0587; found 409.0597.

1H NMR of 13 (700 MHz, D2O)

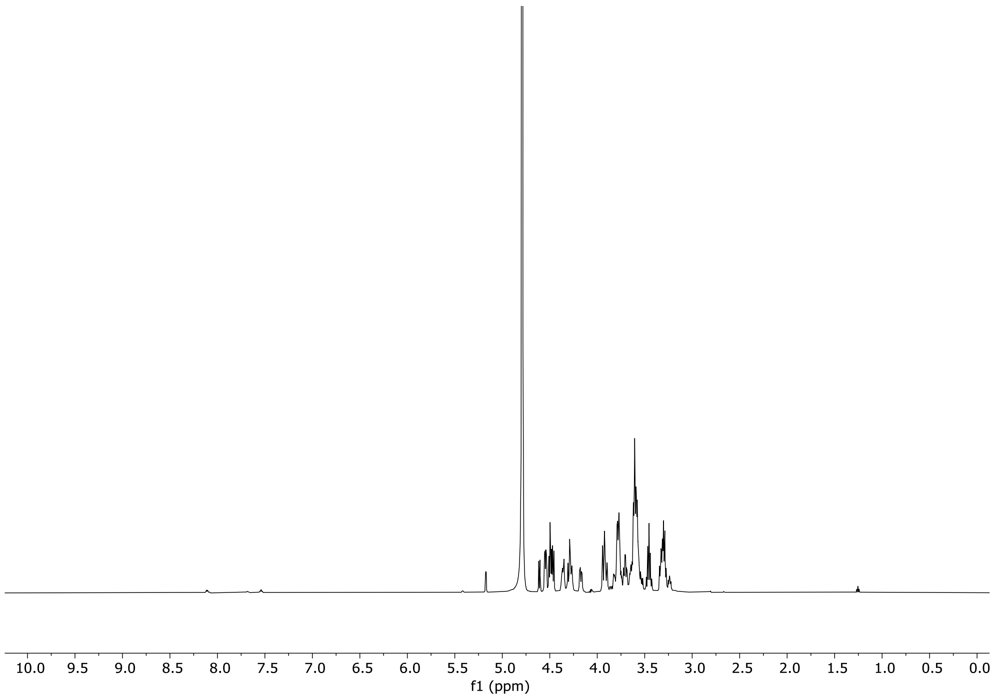

^{13}C NMR of 13 (176 MHz, D_2O)

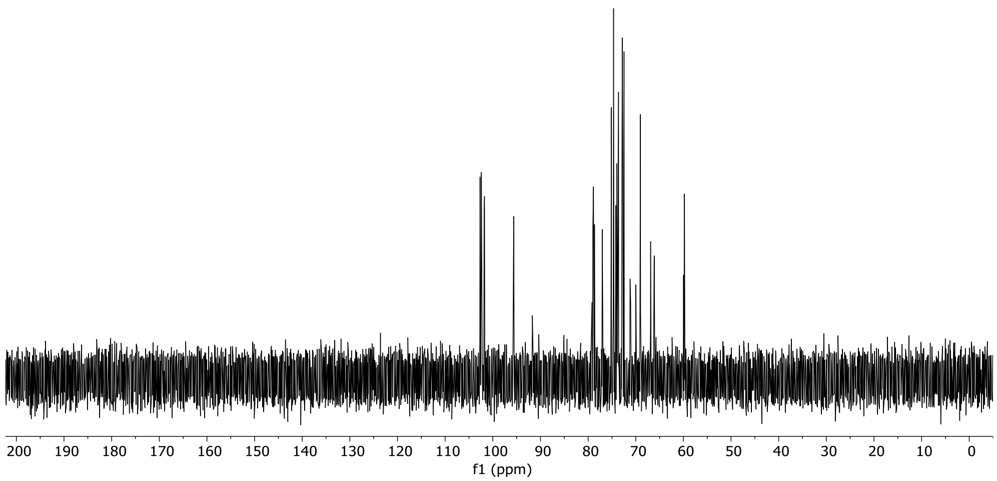

HSQC NMR of 13 (D_2O)

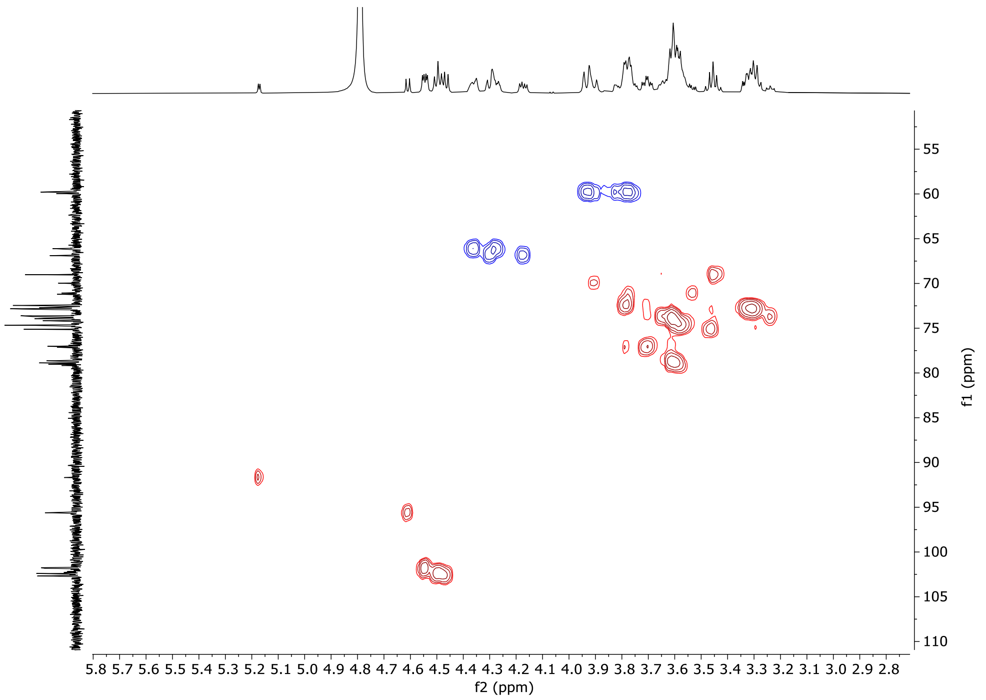

RP-HPLC of 13 (ELSD trace, Method C1, t_R= 23.70 min and 25.0 min)

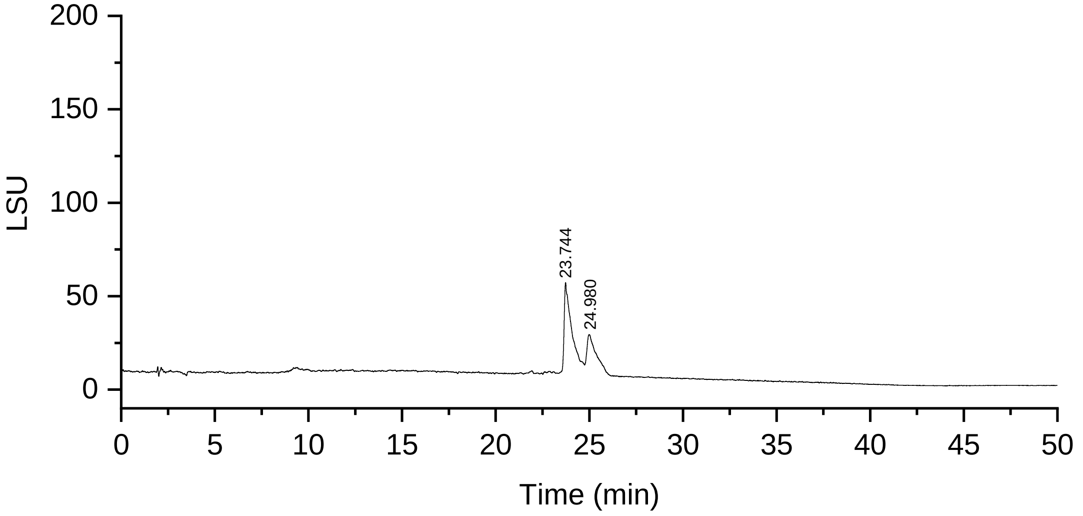

10.10 Synthesis of 14

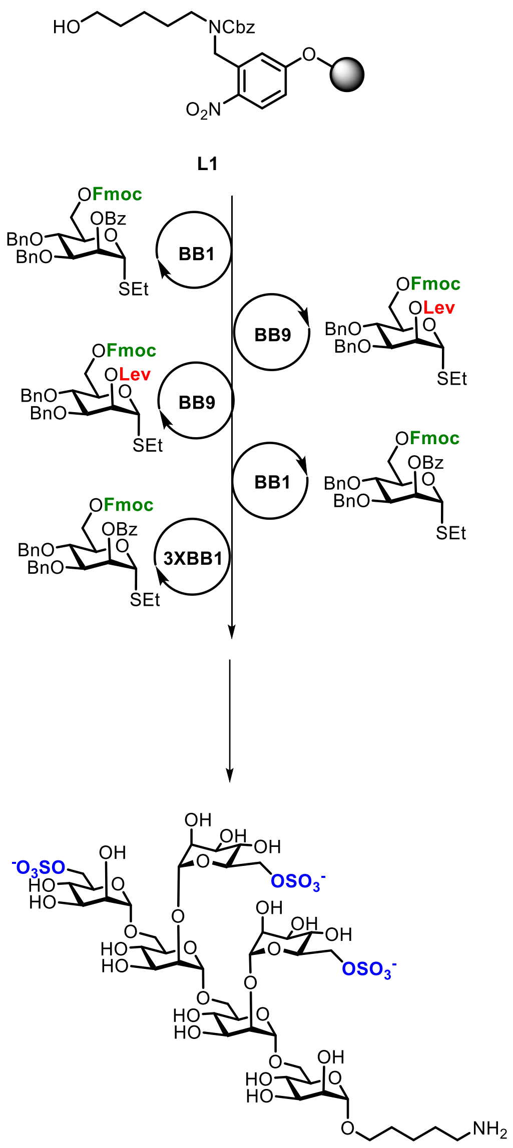

1) AGA:

2) Post-AGA

14

Step		Modules	Notes
AGA		**A**	
	**BB1**	**B, C1, D, E1**	**C1:** (**BB1**, -20 °C for 5 min, 0 °C for 20 min)
	**BB9**	**B, C1, D, E1**	**C1:** (**BB9**, -20 °C for 5 min, 0 °C for 20 min)
	**BB9**	**B, C1, D, E1**	**C1:** (**BB9**, -20 °C for 5 min, 0 °C for 20 min)
	**BB1**	**B, C1, D, E2**	**C1:** (**BB1**, -20 °C for 5 min, 0 °C for 20 min)
	**BB1**	**B, C1(x3), D, E1**	**C1:** (**BB1**, -20 °C for 5 min, 0 °C for 20 min)
Post-AGA	**Sulfation**	**F**	
	**Hydrolysis**	**G**	**G:** (12 h)
	**Hydrogenolysis**	**I**	**I:** 10% Pd/C (12 h)
	**Purification**	**J(B1), J(A1), K**	

Compound **14** was obtained as a white solid (4.8 mg, 30% overall yield).

Analytical data for **14**: ^{1}H NMR (600 MHz, D_2O) δ 5.08 (dd, *J* = 12.2, 1.8 Hz, 2H), 5.05 – 4.98 (m, 2H), 4.98 – 4.86 (m, 1H), 4.83 (d, *J* = 1.8 Hz, 1H), 4.36 – 4.17 (m, 6H), 4.11 – 3.54 (m, 32H), 2.98 (q, *J* = 7.2 Hz, 2H), 1.67 (td, *J* = 15.1, 7.4 Hz, 4H), 1.49 – 1.37 (m, 2H); ^{13}C NMR (151 MHz, D_2O) δ 92.7, 92.7, 90.2, 89.9, 88.3, 88.2, 69.5, 69.0, 61.5, 61.4, 61.2, 61.1, 61.0, 60.8, 60.7, 60.6, 60.6, 60.4, 60.2, 60.2, 57.9, 57.70, 57.57, 57.1, 56.9, 56.7, 56.6, 56.5, 55.9, 55.5, 29.7, 18.3, 16.8, 12.8; HRMS (QToF): Calcd for $C_{41}H_{71}NO_{40}S_3$ $[M+H]^{2-}$ 656.6362; found 656.6385.

1H NMR of 14 (600 MHz, D_2O)

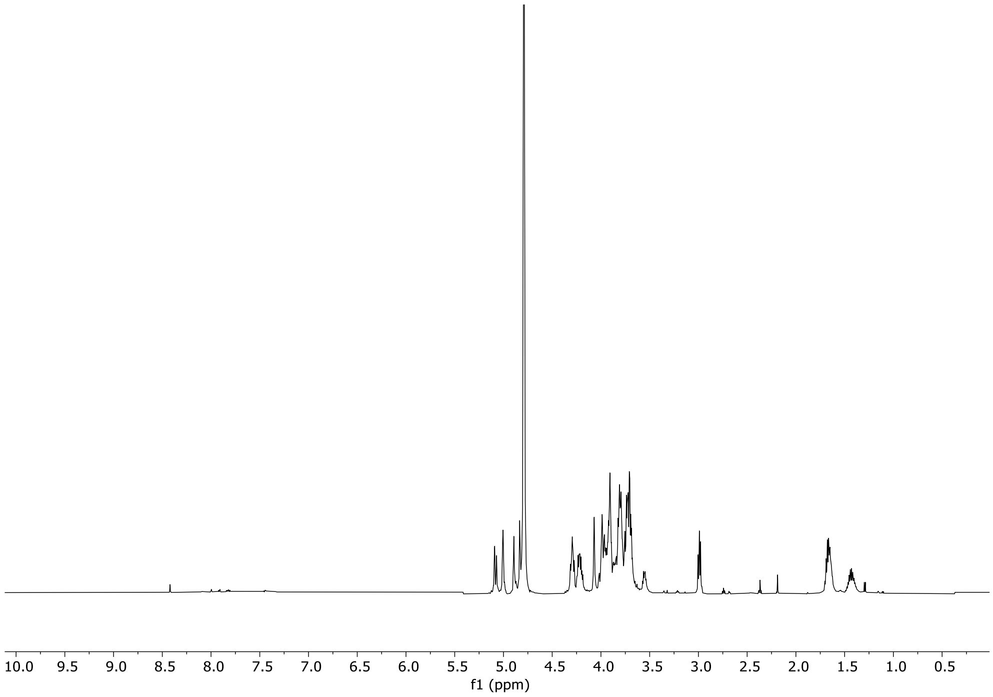

^{13}C NMR of 14 (151 MHz, D_2O)

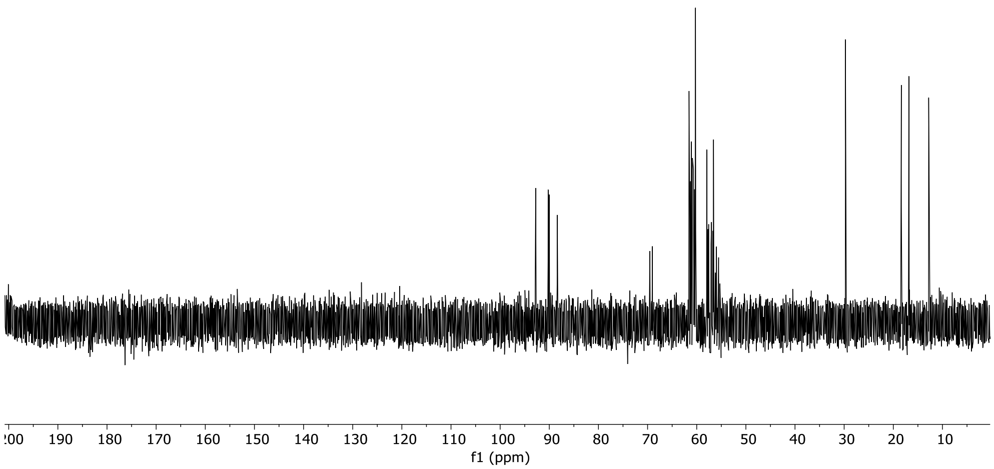

HSQC NMR of 14 (D_2O)

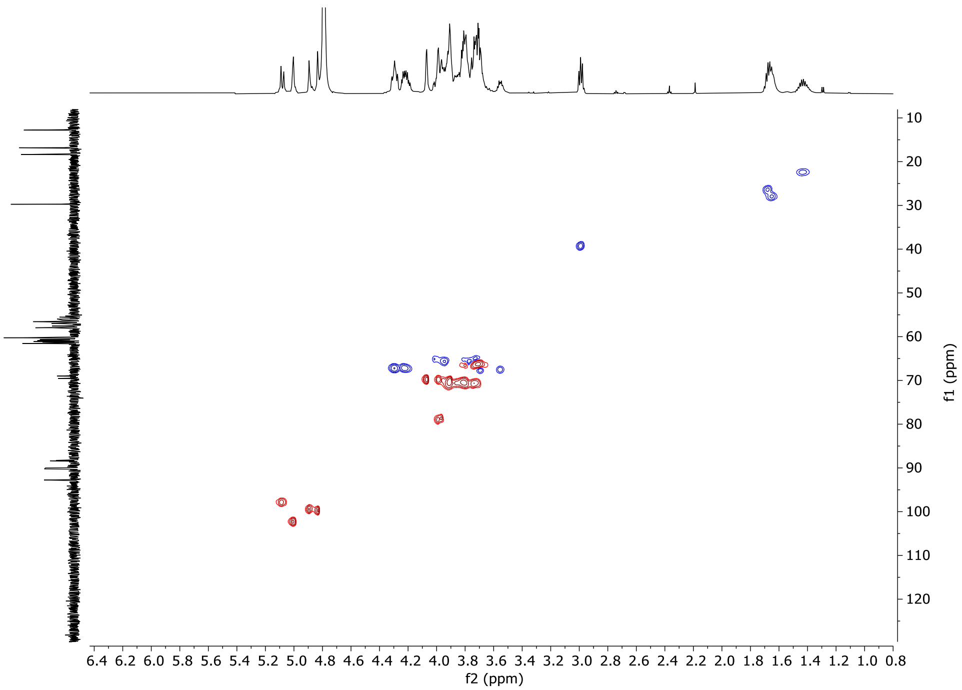

RP-HPLC of 14 (ELSD trace, Method C1, t_R= 14.48 min)

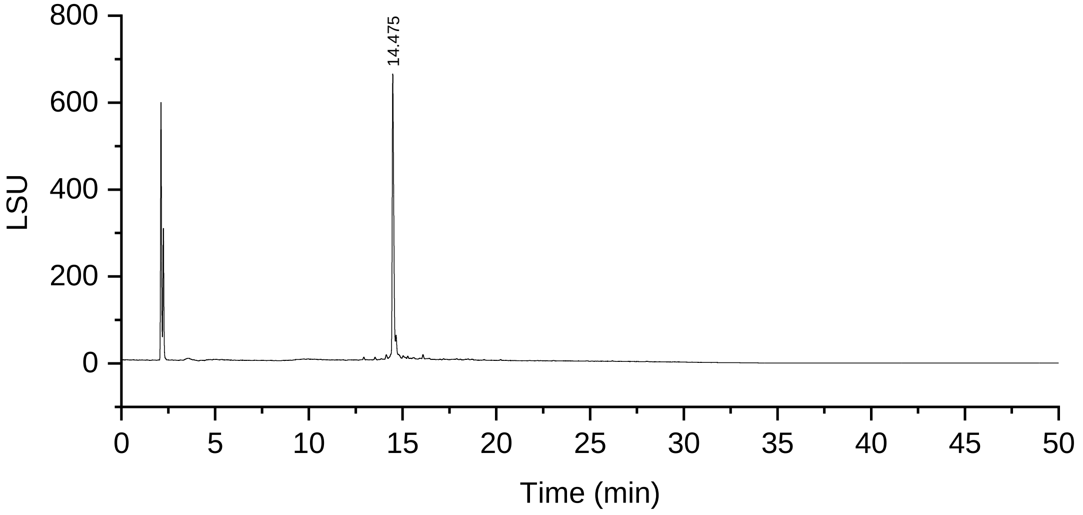

10.11 Synthesis of 15

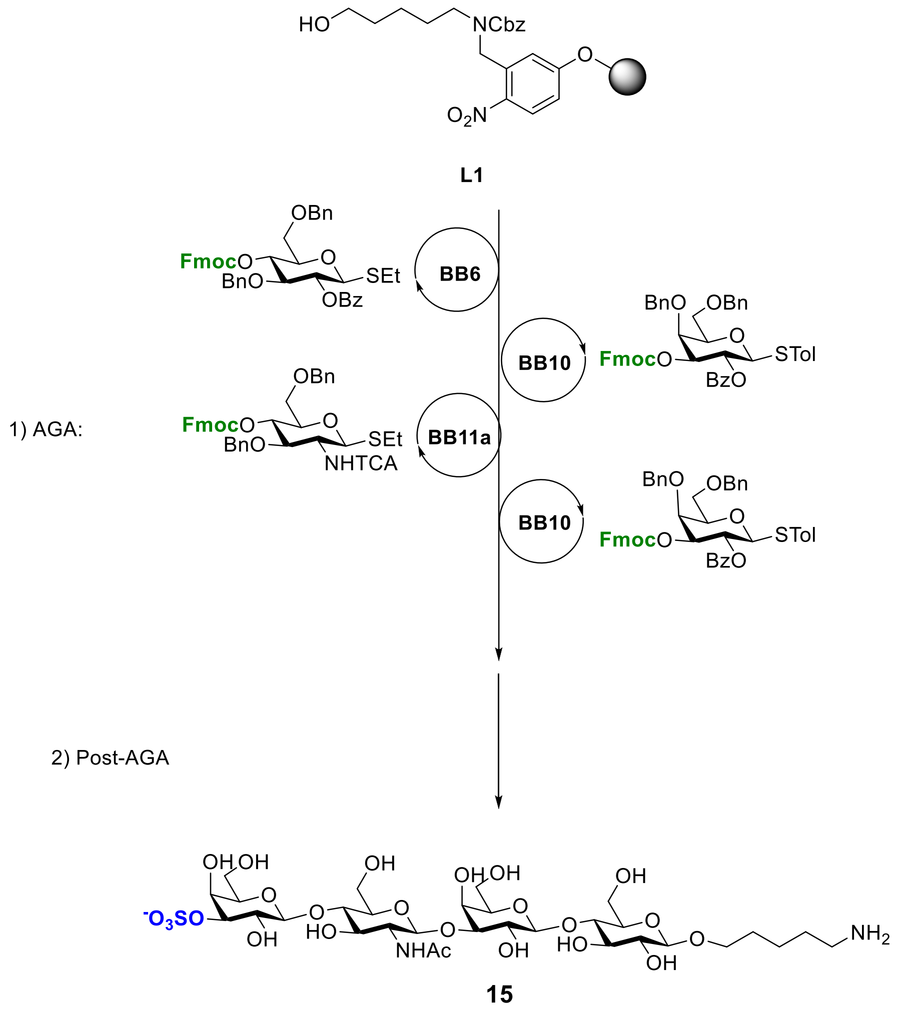

Step		Modules	Notes
		**A**	
AGA	**BB6**	**B, C1(x2), D, E1**	**C1:** (**BB6**, -20 °C for 5 min, 0 °C for 20 min)
	**BB10**	**B, C1(x2), D, E1**	**C1:** (**BB10**, -20 °C for 5 min, 0 °C for 20 min)
	**BB11a**	**B, C1(x2), D, E1**	**C1:** (**BB11a**, -20 °C for 5 min, 0 °C for 20 min)
	**BB10**	**B, C1(x2), D, E1,**	**C1:** (**BB10**, -20 °C for 5 min, 0 °C for 20 min)
Post-AGA	**Sulfation**	**F**	
	**Hydrolysis**	**G**	**G:** (120 h)
	**Hydrogenolysis**	**I1**[a]	**I1:** 10-20% $Pd(OH)_2/C$ (12 h)
	**Purification**	**J(B1), J(A1), K**	

[a] treated with thiourea upon completion of hydrogenolysis

Compound **15** was obtained as a white solid (2 mg, 38% overall yield).

Analytical data for **15**: ^{1}H NMR (700 MHz, D_2O) δ 4.71 (d, *J* = 8.4 Hz, 1H), 4.60 (d, *J* = 7.8 Hz, 1H), 4.49 (d, *J* = 8.0 Hz, 1H), 4.44 (d, *J* = 7.9 Hz, 1H), 4.35 (dd, *J* = 9.9, 3.3 Hz, 1H), 4.30 (d, *J* = 3.3 Hz, 1H), 4.17 (d, *J* = 3.4 Hz, 1H), 4.01 – 3.91 (m, 3H), 3.91 – 3.56 (m, 16H), 3.01 (t, *J* = 7.6 Hz, 3H), 2.04 (s, 3H), 1.69 (dp, *J* = 14.7, 7.2 Hz, 4H), 1.50 – 1.44 (m, 2H)*;* ^{13}C NMR (176 MHz, D_2O) ^{13}C NMR (176 MHz, D_2O) δ 174.9, 102.9, 102.8, 102.4, 101.9, 82.0, 80.0, 78.4, 78.01, 75.0, 74.9, 74.8, 74.5, 74.4, 72.8, 72.2, 70.0, 70.0, 69.1, 68.3, 66.8, 61.0, 60.9, 60.1, 59.8, 55.2, 39.4, 28.1, 26.4, 23.2, 22.2, 22.0*;* HRMS (QToF)**:** Calcd for $C_{31}H_{55}N_2O_{24}S$ $[M]^-$ 871.2870; found 871.2866.

1H NMR of 15 (700 MHz, D_2O)

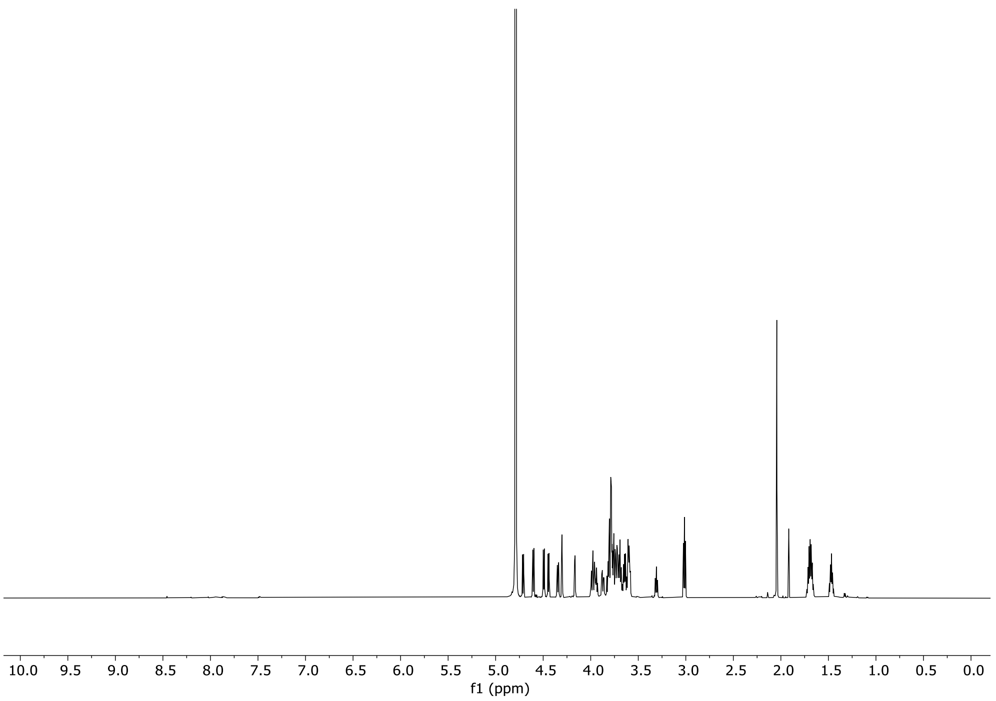

^{13}C NMR of 15 (176 MHz, D_2O)

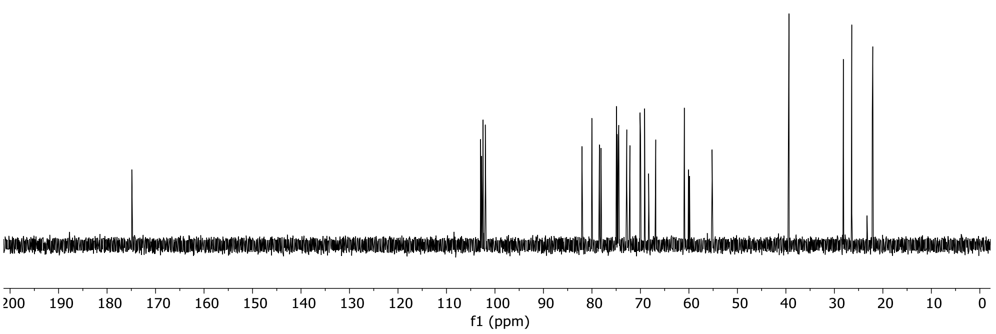

HSQC NMR of 15 (D_2O)

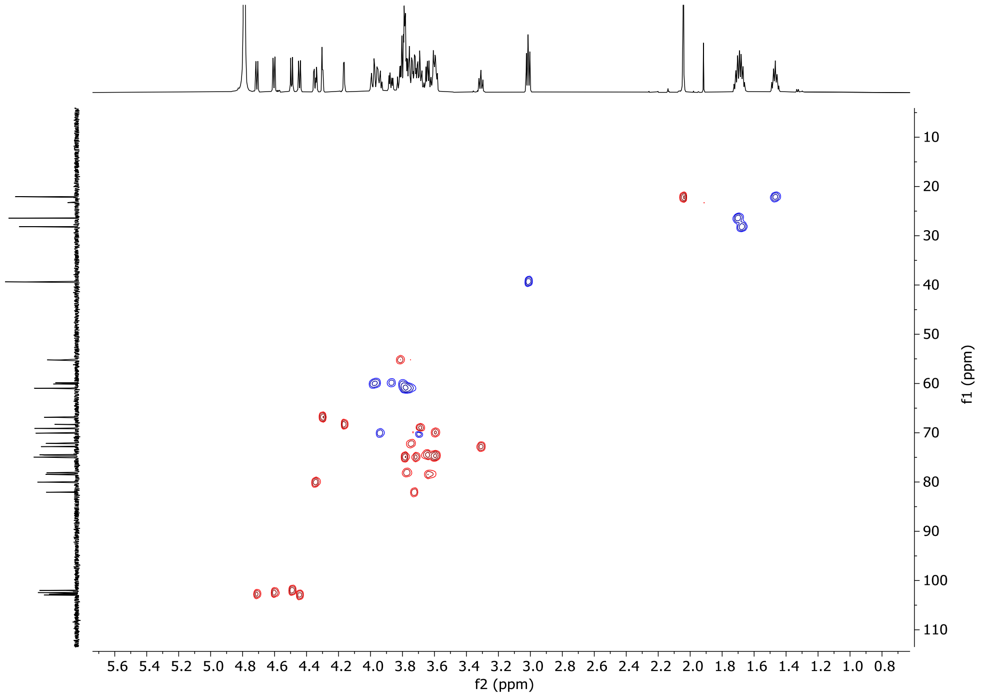

RP-HPLC of 15 (ELSD trace, Method C1, t_R= 18.50 min)

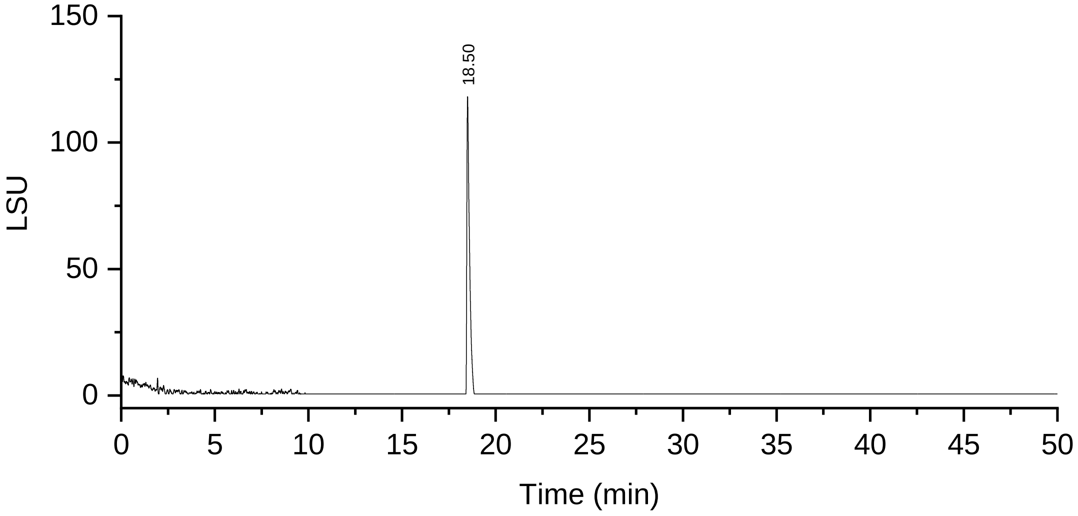

10.12 Synthesis of 16

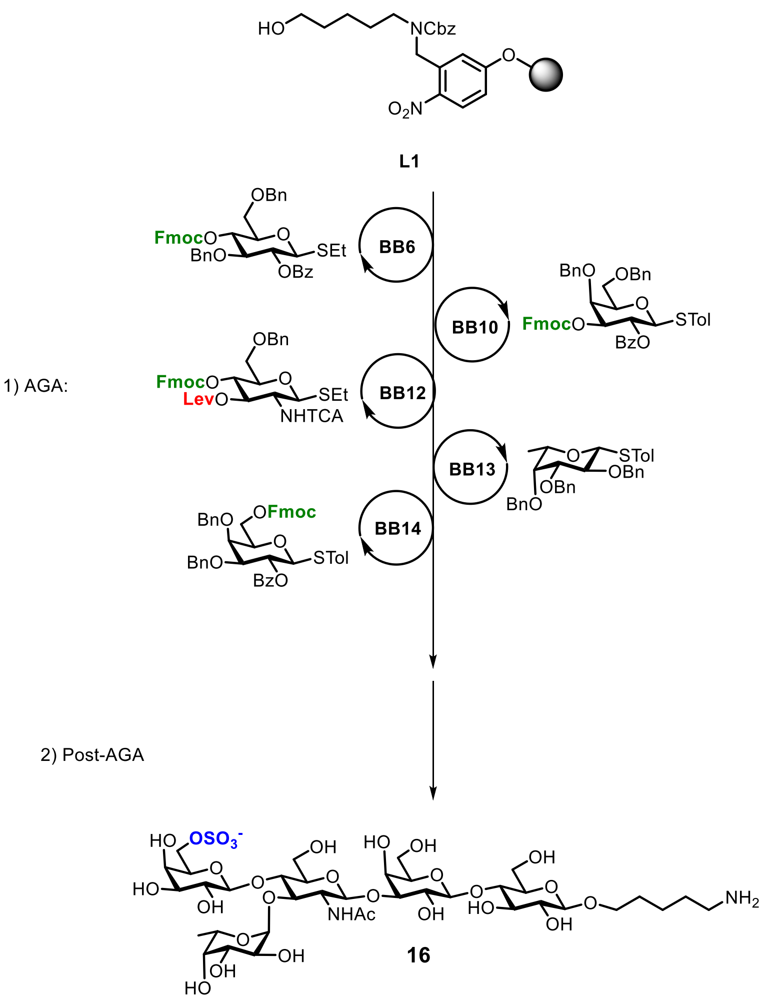

Step		Modules	Notes
AGA		**A**	
	**BB6**	**B, C1, D, E1**	**C1:** (**BB6**, **-**20 °C for 5 min, 0 °C for 20 min)
	**BB10**	**B, C1, D, E1**	**C1:** (**BB10**, **-**20 °C for 5 min, 0 °C for 20 min)
	**BB12**	**B, C1(x2), D, E2**	**C1:** (**BB12**, **-**20 °C for 5 min, 0 °C for 20 min)
	**BB13**	**B, C1(x2), D, E1**	**C1:** (**BB13**, **-**20 °C for 5 min, 0 °C for 20 min)
	**BB14**	**B, C1(x2), D, E1,**	**C1:** (**BB14**, **-**20 °C for 5 min, 0 °C for 20 min)
Post-AGA	**Sulfation**	**F**	
	**Hydrolysis**	**G**	**G:** (120 h)
	**Hydrogenolysis**	**I1**[a]	**I1:** 10-20% $Pd(OH)_2$/C (12 h)
	**Purification**	**J(B1), J(A1), K**	

[a] treated with thiourea upon completion of hydrogenolysis

Compound **16** was obtained as a white solid (1 mg, 7% overall yield).

Analytical data for **16**: ^{1}H NMR (700 MHz, D_2O) δ 5.12 (d, *J* = 4.0 Hz, 1H), 4.72 (d, *J* = 8.3 Hz, 1H), 4.49 (d, *J* = 8.0 Hz, 2H), 4.44 (d, *J* = 7.8 Hz, 1H), 4.21 – 4.15 (m, 3H), 4.00 – 3.51 (m, 26H), 3.01 (t, *J* = 7.5 Hz, 2H), 2.03 (s, 3H), 1.69 (dt, *J* = 14.8, 7.6 Hz, 4H), 1.47 (p, *J* = 7.9 Hz, 2H), 1.18 (d, *J* = 6.6 Hz, 3H)*;* ^{13}C NMR (176 MHz, D_2O) δ 174.6, 102.9, 102.5, 102.0, 101.6, 98.5, 82.1, 78.45, 75.2, 74.0, 74.9, 74.8, 74.5, 73.4, 72.8, 72.3, 72.2, 72.0, 70.9, 70.0, 69.9, 69.1, 68.3, 67.9, 67.8, 67.0, 66.7, 61.0, 60.1, 59.8, 56.0, 39.4, 28.1, 26.4, 22.3, 22.0, 20.0, 15.3*;* HRMS (QToF): Calcd for $C_{37}H_{65}N_2O_{28}S$ $[M]^-$ 1017.3450; found 1017.3487.

1H NMR of 16 (700 MHz, D_2O)

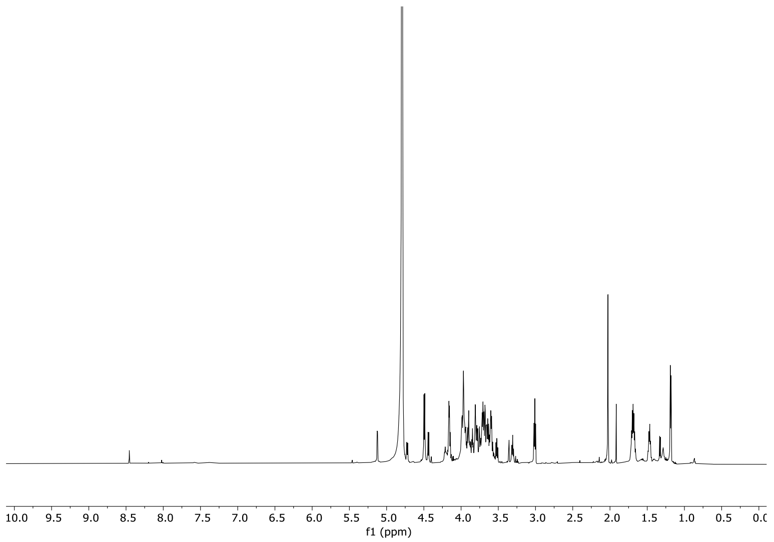

^{13}C NMR of 16 (176 MHz, D_2O)

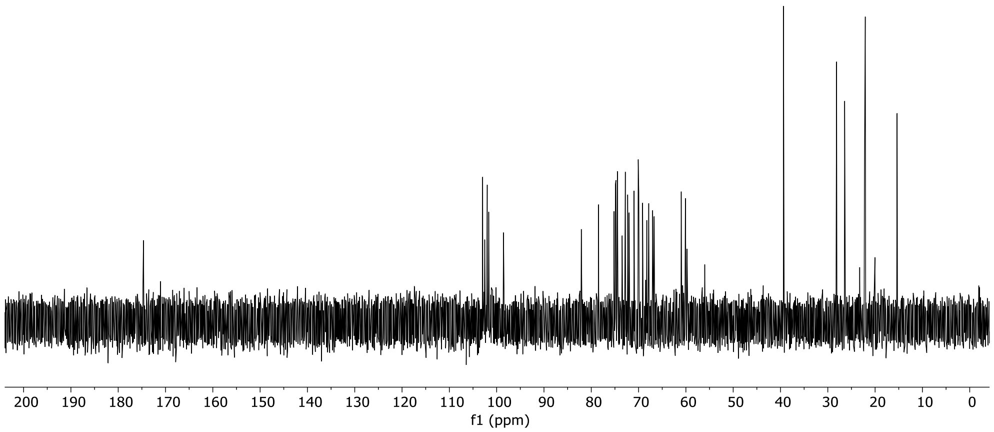

HSQC NMR of 16 (D_2O)

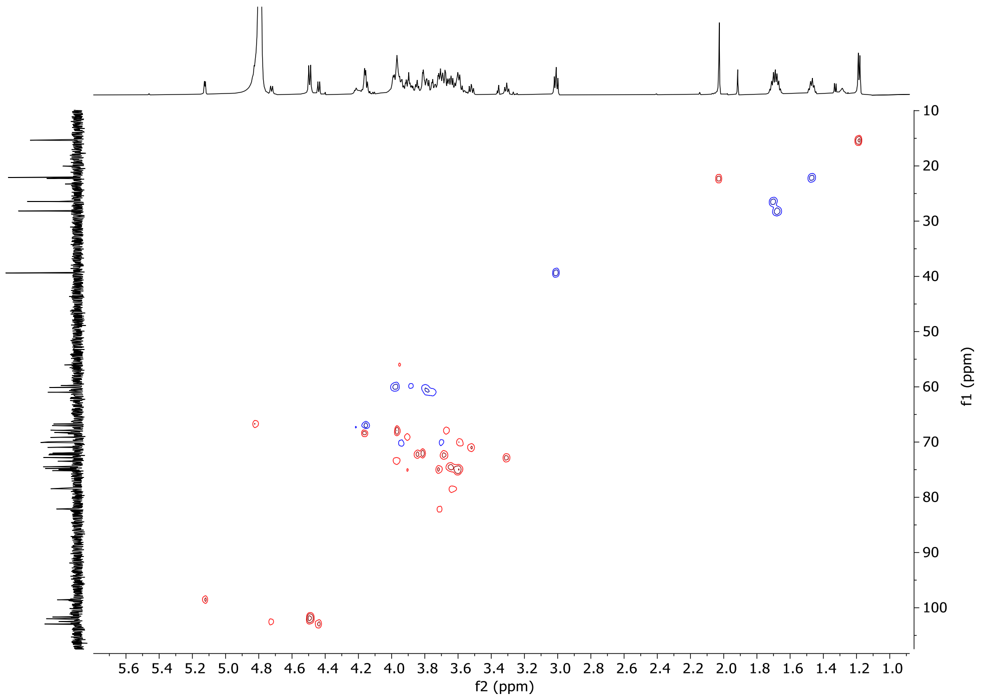

RP-HPLC of 16 (ELSD trace, Method C1, t_R= 16.12 min)

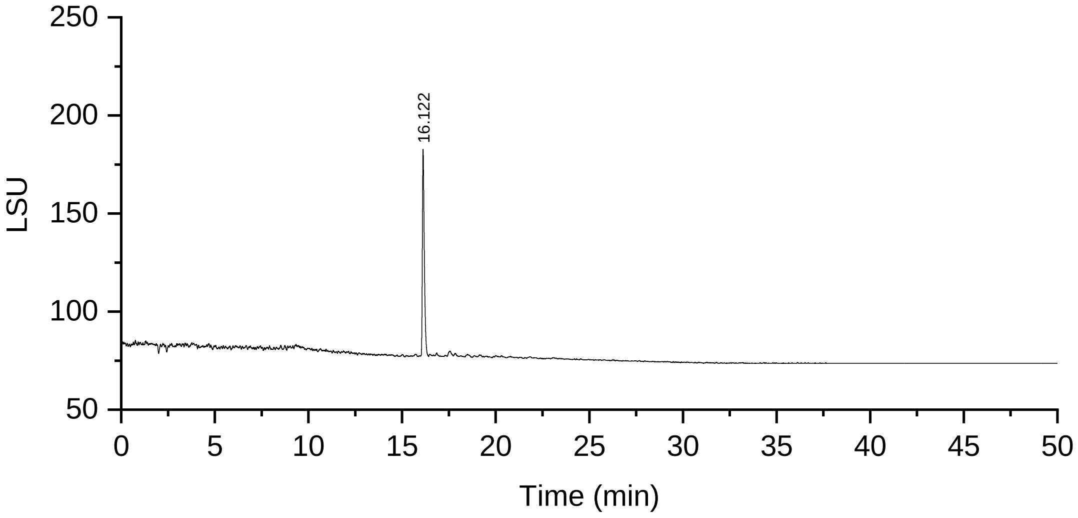

10.13 Synthesis of 17

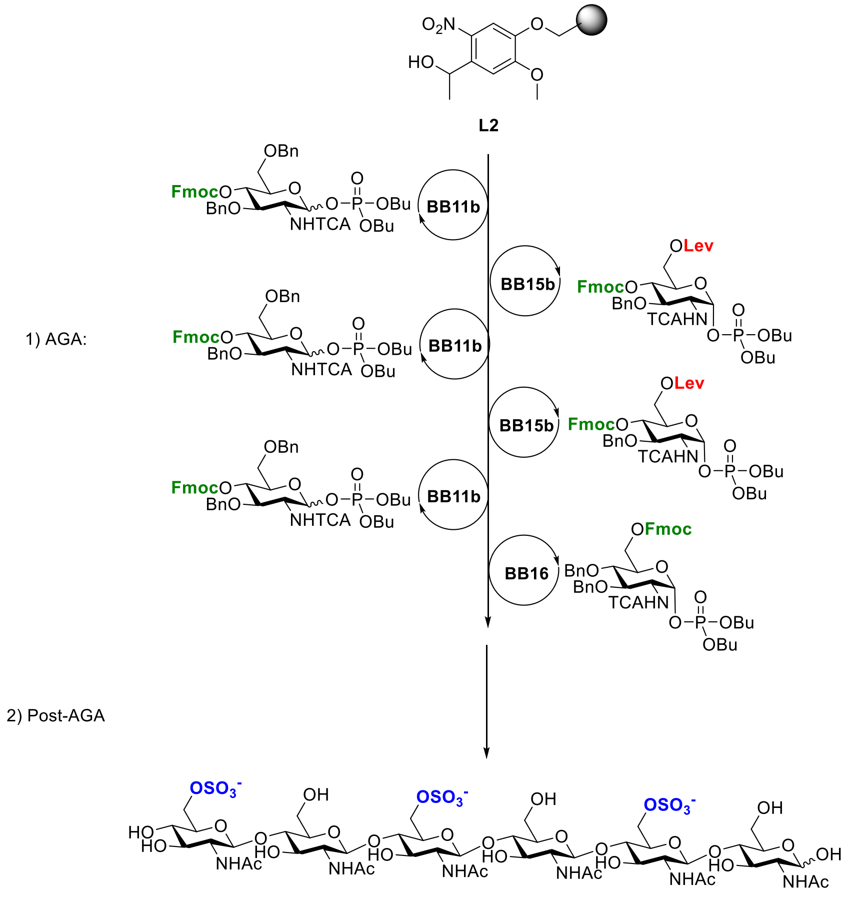

17

Step		Modules	Notes
AGA		**A**	
	**BB11b**	**B, C2, D, E1**	**C2:** (**BB11b**, -30 °C for 5 min, -10 °C for 40 min)
	**BB15b**	**B, C2, D, E1**	**C2:** (**BB15b**, -30 °C for 5 min, -10 °C for 40 min)
	**BB11b**	**B, C2, D, E1**	**C2:** (**BB11b**, -30 °C for 5 min, -10 °C for 40 min)
	**BB15b**	**B, C2, D, E1**	**C2:** (**BB15b**, -30 °C for 5 min, -10 °C for 40 min)
	**BB11b**	**B, C2, D, E1**	**C2:** (**BB11b**, -30 °C for 5 min, -10 °C for 40 min)
	**BB16**	**B, C2, D, E1, E2**	**C2:** (**BB16**, -30 °C for 5 min, -10 °C for 40 min)
Post-AGA	**Sulfation**	**F**	
	**Hydrogenolysis**	**I1**	**I1:** 10-20% $Pd(OH)_2/C$ (24 h)
	**Purification**	**J(B1), J(A1), K**	

Compound **17** was obtained as a white solid (0.7 mg, 6% overall yield).

Analytical data for **17**: ^{1}H NMR (700 MHz, D_2O) δ 5.20 (d, *J* = 3.7 Hz, 0.60H, α-H1), 4.69 (d, *J* = 6.1 Hz, 0.40H, β-H1), 4.61 (dt, *J* = 14.0, 6.5 Hz, 5H), 4.36 (d, *J* = 10.8 Hz, 1H), 4.29 (d, *J* = 10.8 Hz, 2H), 4.23 (dd, *J* = 11.3, 5.5 Hz, 1H), 4.12 (dd, *J* = 12.7, 5.9 Hz, 2H), 3.95 – 3.46 (m, 31H), 2.11 – 2.03 (m, 18H); ^{13}C NMR (176 MHz, D_2O) δ 101.5, 101.4, 101.3, 101.2, 101.1, 94.6, 90.2.*Only the anomeric carbons are reported due to low amount; HRMS (QToF): Calcd for $C_{48}H_{77}N_6O_{40}S_3$ $[M]^{3-}$ 491.1118; found 491.1127.

^{1}H NMR of 17 (700 MHz, D_2O)

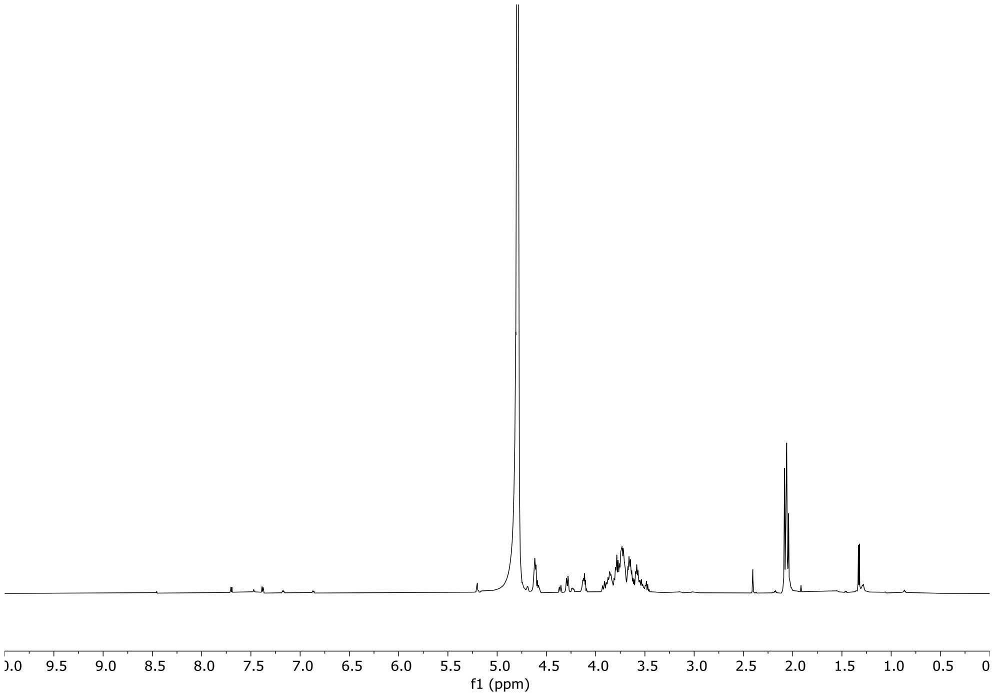

^{13}C NMR of 17 (176 MHz, D_2O)

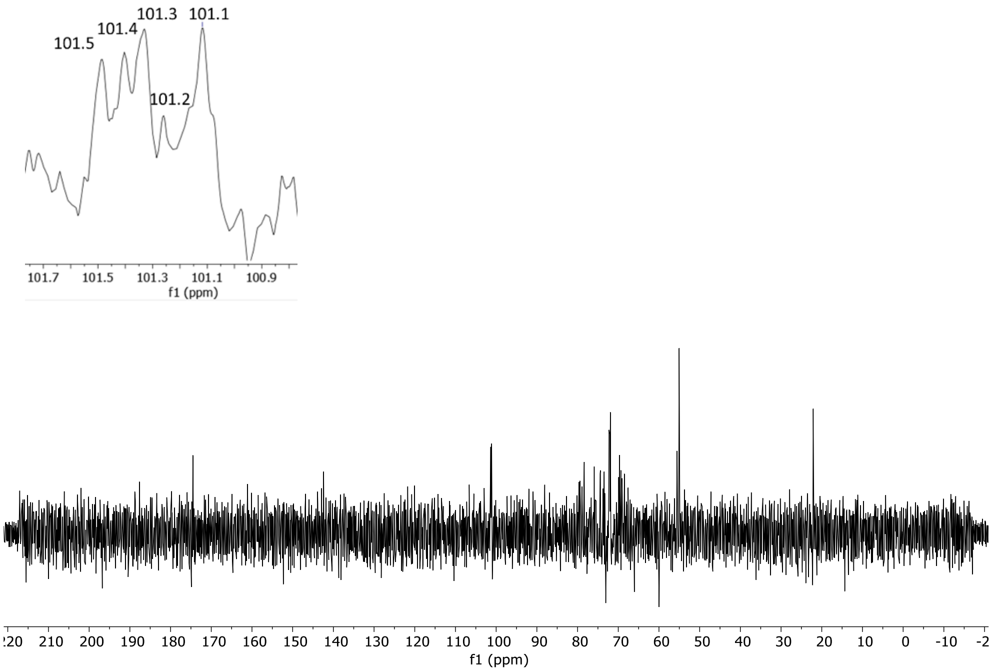

HSQC NMR of 17 (D_2O)

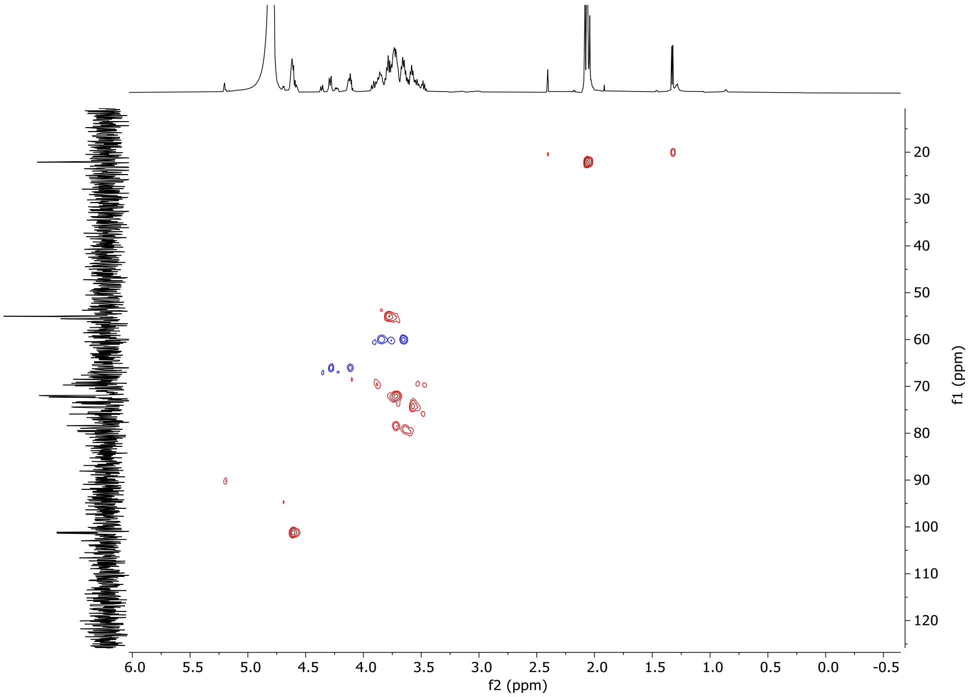

RP-HPLC of 17 (ELSD trace, Method C1, t_R= 16.2 min)

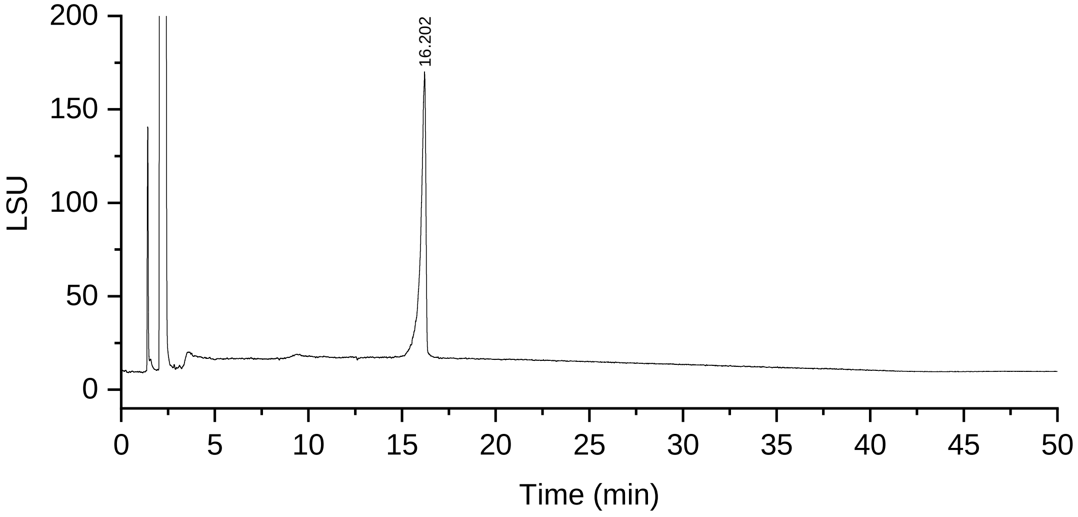

10.14 Synthesis of 18

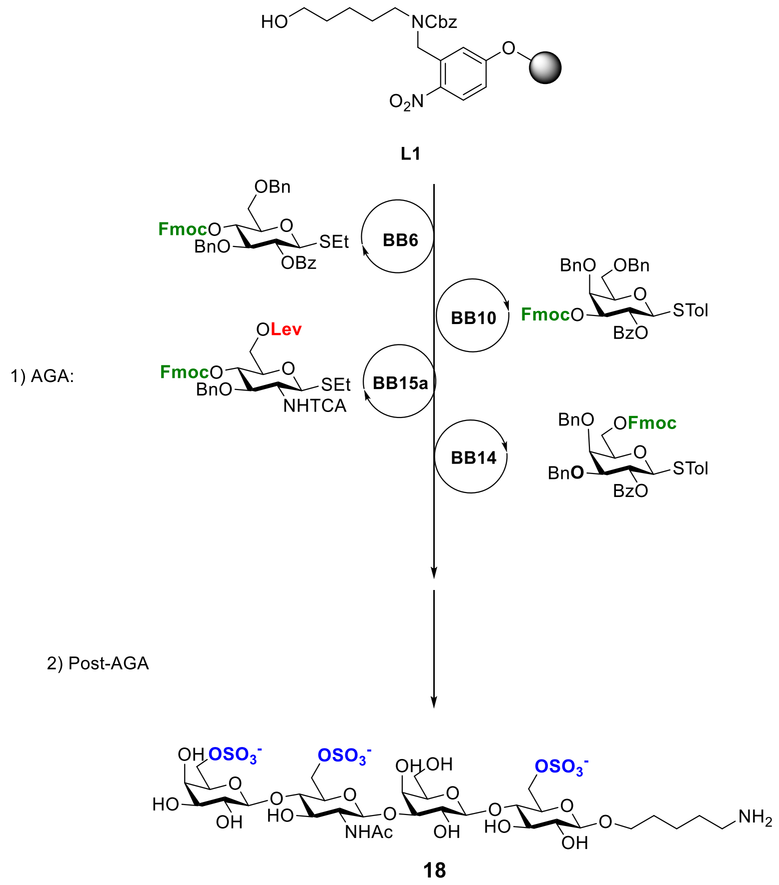

Step		Modules	Notes
		**A**	
	**BB6**	**B, C1(x2), D, E1**	**C1:** (**BB6**, -20 °C for 5 min, 0 °C for 20 min)
AGA	**BB10**	**B, C1(x2), D, E1**	**C1:** (**BB10**, -20 °C for 5 min, 0 °C for 20 min)
	**BB15a**	**B, C1(x2), D, E1**	**C1:** (**BB15a**, -20 °C for 5 min, 0 °C for 20 min)
	**BB14**	**B, C1(x2), D, E1, E2**	**C1:** (**BB14**, -20 °C for 5 min, 0 °C for 20 min)
	**Sulfation**	**F**	
	**Hydrolysis**	**G**	**G:** (120 h)
Post-AGA	**Acetylation**		**G1**
	**Hydrogenolysis**	**I1[a]**	**I1:** 10-20% $Pd(OH)_2/C$ (12 h)
	**Purification**	**J(B1), J(A1), K**	

[a] treated with thiourea upon completion of hydrogenolysis

Compound **18** was obtained as a white solid (0.7 mg, 6% overall yield).

Analytical data for **18**: ^{1}H NMR (700 MHz, D_2O) δ 4.74 – 4.71 (m, 1H), 4.57 – 4.48 (m, 3H), 4.44 – 4.39 (m, 1H), 4.34 – 4.18 (m, 5H), 4.02 – 3.52 (m, 20H), 3.37 – 3.30 (m, 1H), 3.02 (t, *J* = 7.6 Hz, 2H), 2.05 (s, 2H), 1.69 (dp, *J* = 18.0, 6.6 Hz, 4H), 1.52 – 1.42 (m, 2H); ^{13}C NMR (176 MHz, D_2O) δ 174.9, 102.9, 102.8, 102.7, 102.1, 82.4, 78.7, 77.7, 75.0, 74.4, 74.4, 72.7, 72.5, 72.3, 72.3, 72.2, 72.1, 70.8, 70.3, 69.9, 68.3, 68.2, 67.0, 66.6, 66.4, 61.1, 55.1, 39.4, 39.3, 28.2, 26.2, 22.2, 22.2, 22.0; HRMS (QToF): Calcd for $C_{31}H_{54}N_2O_{30}S_3^{3-}$ $[M]^{2-}$ 515.0967; found 515.0961.

^{1}H NMR of 18 (700 MHz, D_2O)

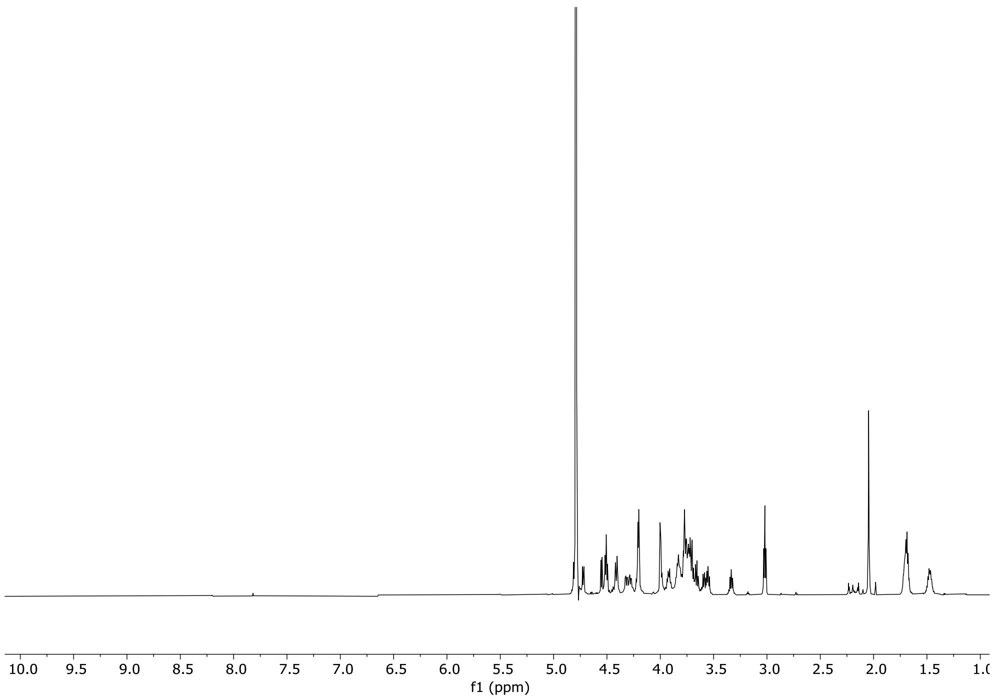

^{13}C NMR of 18 (176 MHz, D_2O)

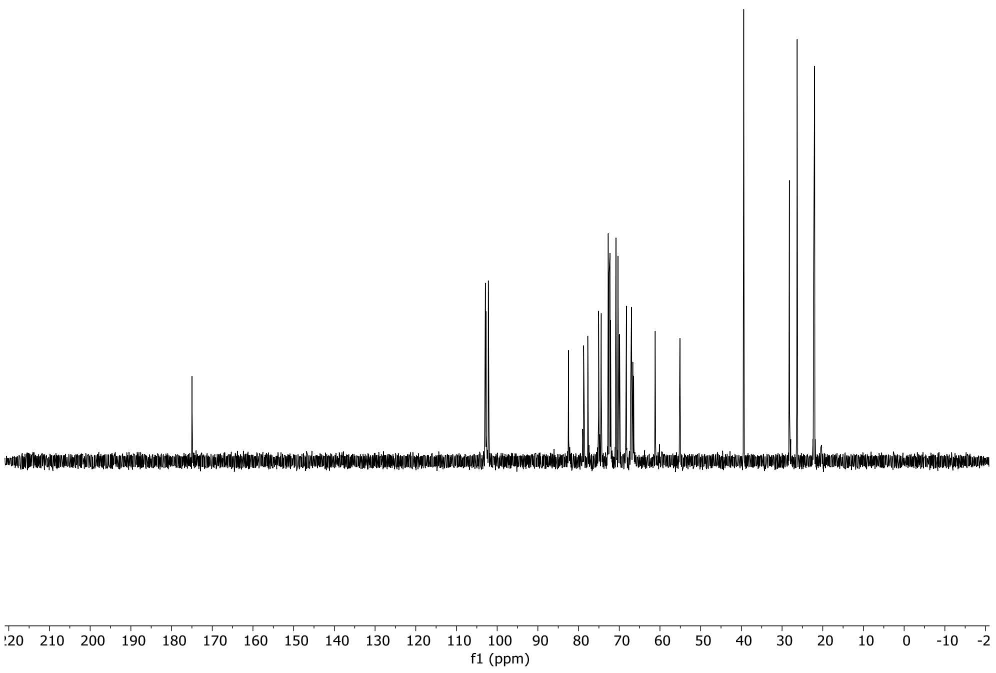

HSQC NMR of 18 (D_2O)

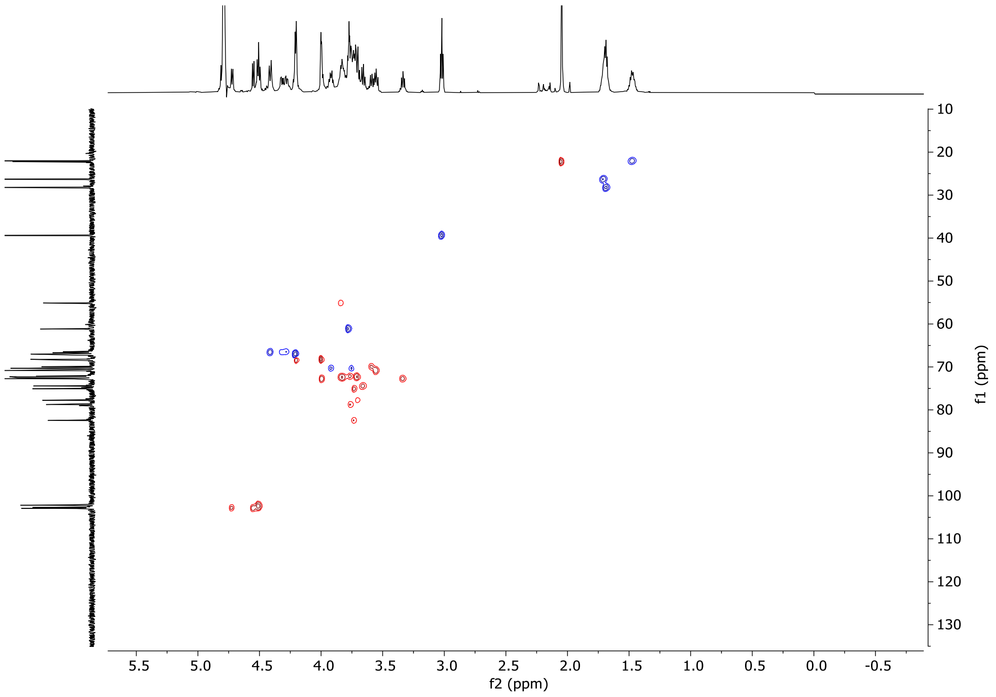

RP-HPLC of 18 (ELSD trace, Method C1, t_R= 17.42 min)

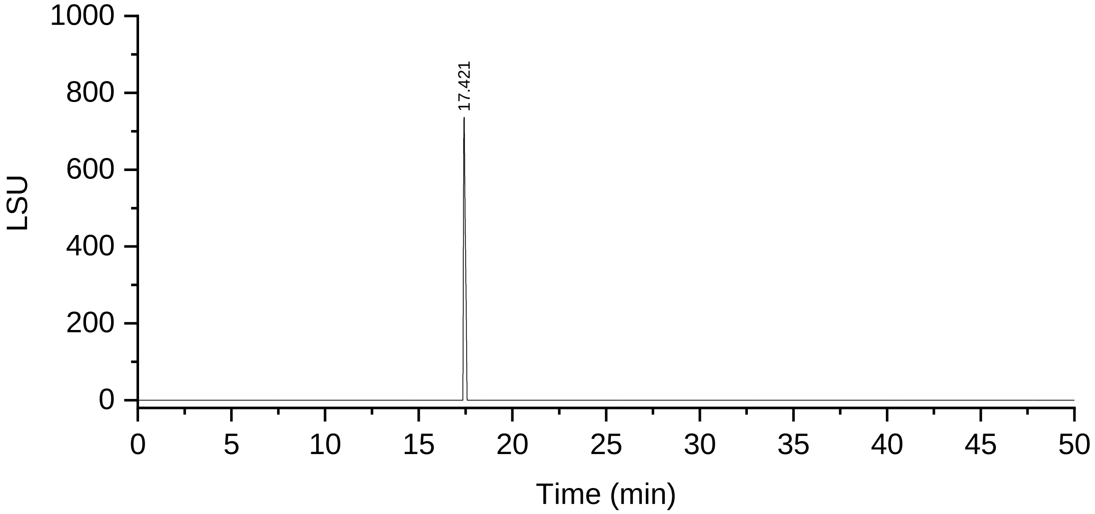

10.15 Synthesis of 19

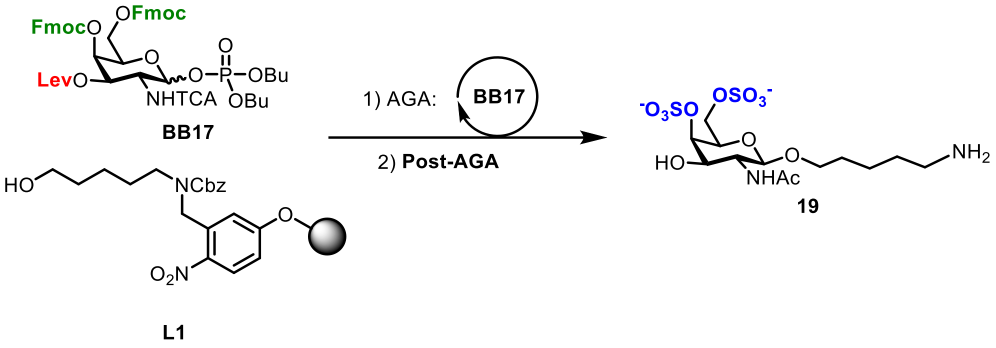

Step		Modules	Notes
AGA		**A**	
	**BB17**	**B, C2, D, E1**	**C2:** (**BB17,**-30 °C for 5 min, -10 °C for 40 min)
Post-AGA	**Sulfation**	**F**	
	**Hydrolysis**	**G**	**G:** (12 h)
	**Acetylation**		**G1**
	**Hydrolysis**	**G**	**G:** (6 h)
	**Hydrogenolysis**	**I**	**I:** 10% Pd/C (12 h)
	**Purification**	**J(B1), J(A1), K**	

Compound **19** was obtained as a white solid (2 mg, 31% overall yield).

Analytical data for **19**: ^{1}H NMR (400 MHz, D_2O) δ 4.71 – 4.67 (m, 1H), 4.53 – 4.48 (m, 1H), 4.30 (dd, *J* = 11.4, 2.7 Hz, 1H), 4.20 (t, *J* = 10.3 Hz, 1H), 4.08 – 4.01 (m, 1H), 3.92 – 3.83 (m, 3H), 3.70 – 3.61 (m, 1H), 2.97 (t, *J* = 7.8 Hz, 2H), 2.02 (s, 3H), 1.71 – 1.56 (m, 4H), 1.49 – 1.34 (m, 2H); ^{13}C NMR (101 MHz, D_2O) δ 174.7, 101.4, 75.5, 72.1, 70.3, 69.9, 67.9, 52.6, 39.3, 28.1, 26.3, 22.1, 21.9.; HRMS (QToF): Calcd for $C_{13}H_{25}N_2O_{12}S_2$ $[M+H]^-$ 465.0865; found 465.0875.

^{1}H NMR of 19 (400 MHz, D_2O)

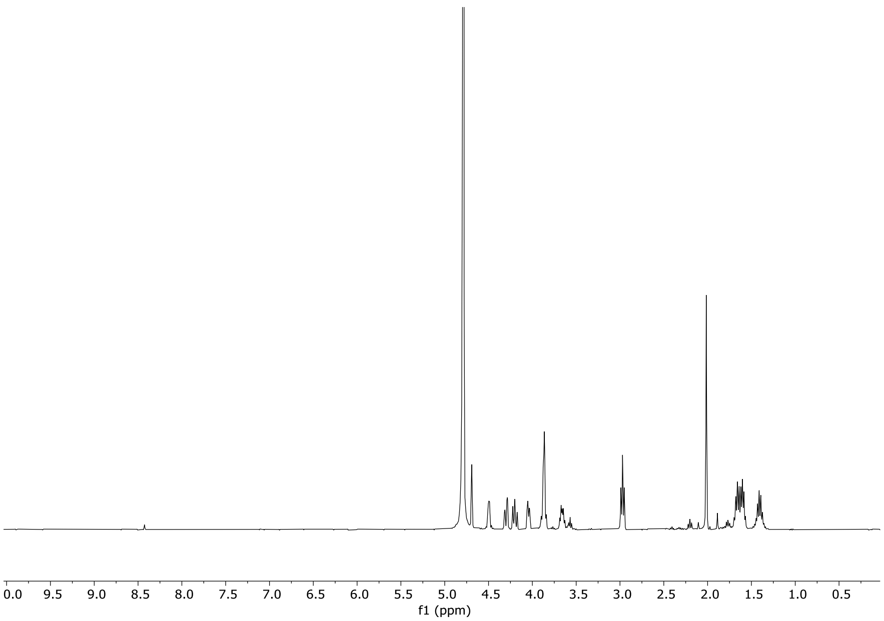

^{13}C NMR of 19 (101 MHz, D_2O)

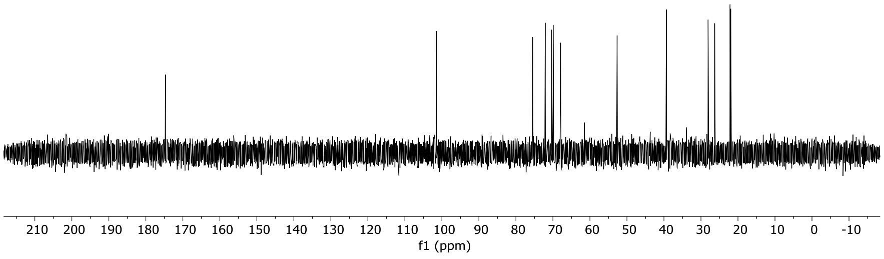

HSQC NMR of 19 (D_2O)

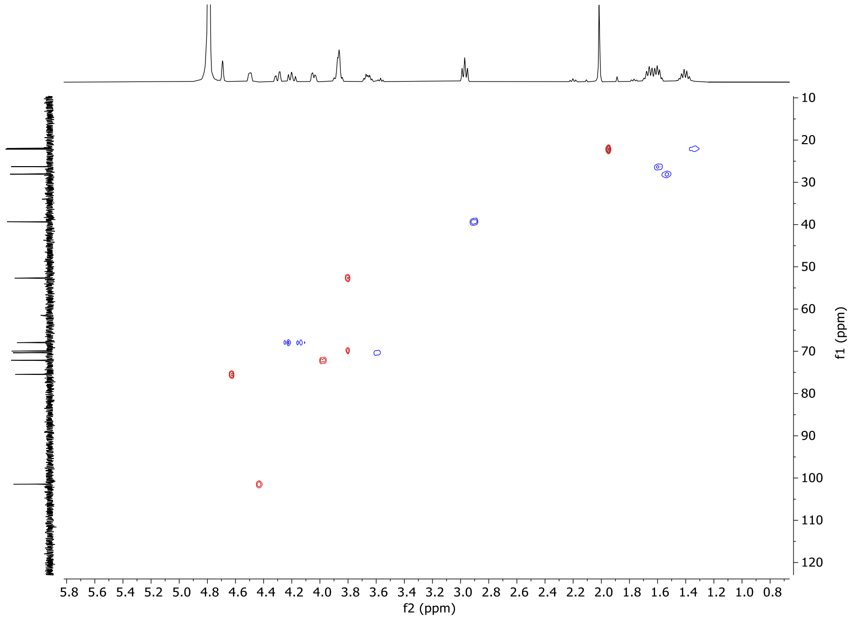

RP-HPLC of 19 (ELSD trace, Method C_1, t_R= 13.22 min)

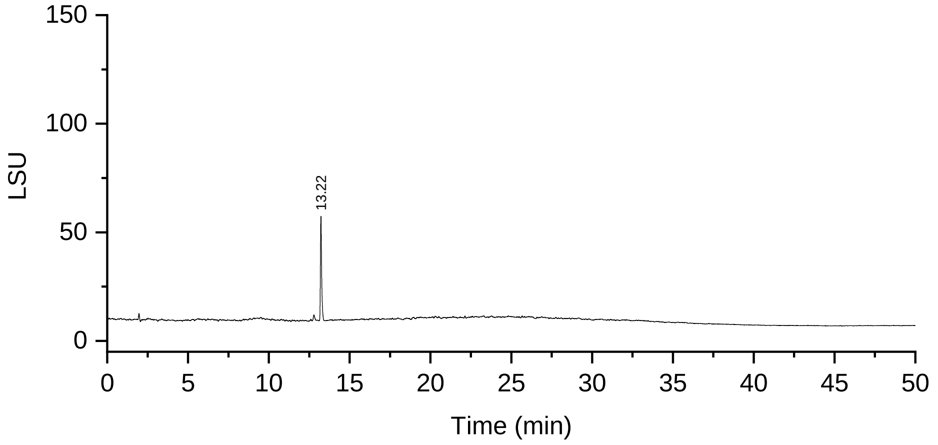

10.16 Synthesis of 20

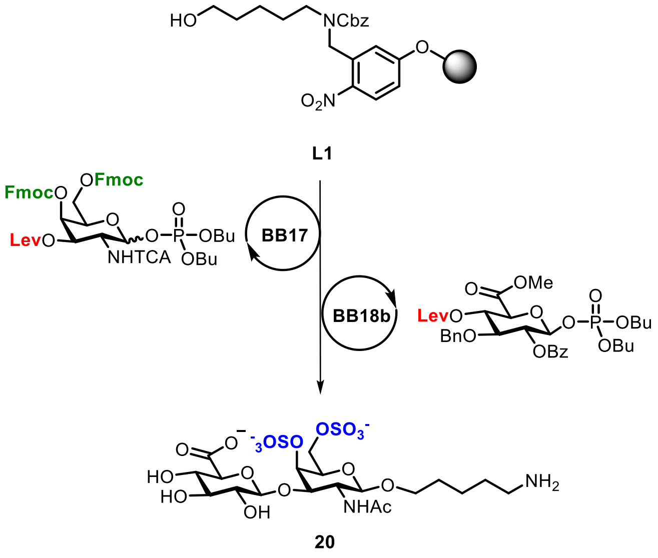

Step		Modules	Notes
AGA		**A**	
	**BB17**	**B, C2, D, E2**	**C2:** (**BB17,**-30 °C for 5 min, -10 °C for 40 min)
	**BB18b**	**B, C2(x2), D, E2, E1***	**C2:** (**BB18b,**-30 °C for 5 min, -10 °C for 40 min)
Post-AGA	**Sulfation**	**F**	
	**Hydrolysis**	**G**	**G:** (12 h)
	**Acetylation**		**G1**
	**Hydrolysis**	**G**	**G:** (6 h)
	**Hydrogenolysis**	**I**	**I:** 10% Pd/C (12 h)
	**Purification**	**J(B1), J(A1), K**	

*To avoid possible elimination side-reactions, the Fmoc deprotection E1 was carried out with 5% of TEA in DMF .

Compound **20** was obtained as a white solid (1.5 mg, 17% overall yield).

Analytical data for **20**: ^{1}H NMR (600 MHz, D_2O) δ 4.51 (d, *J* = 8.3 Hz, 1H), 4.47 (d, *J* = 7.8 Hz, 1H), 4.29 (dd, *J* = 11.4, 2.8 Hz, 1H), 4.19 (dd, *J* = 11.4, 9.0 Hz, 1H), 4.10 – 4.02 (m, 3H), 3.88 (dt, *J* = 10.3, 6.2 Hz, 1H), 3.70 – 3.67 (m, 1H), 3.65 (d, *J* = 9.8 Hz, 1H), 3.52 (t, *J* = 9.4 Hz, 1H), 3.45 (t, *J* = 9.2 Hz, 1H), 3.36 (dd, *J* = 9.4, 7.8 Hz, 1H), 2.98 (t, *J* = 7.6 Hz, 2H), 2.01 (s, 3H), 1.70 – 1.59 (m, 4H), 1.46 – 1.37 (m, 2H); ^{13}C NMR (151 MHz,

D_2O) δ 175.9, 174.6, 103.0, 101.2, 76.4, 76.1, 75.1, 74.3, 72.4, 72.2, 71.7, 70.3, 68.0, 51.7, 39.3, 28.0, 26.2, 22.2, 21.9*;* HRMS (QToF): Calcd for $C_{19}H_{33}N_2O_{18}S_2$ [M+H+H]$^-$ 641.1175; found 641.1179.

^{1}H NMR of 20 (600 MHz, D_2O)

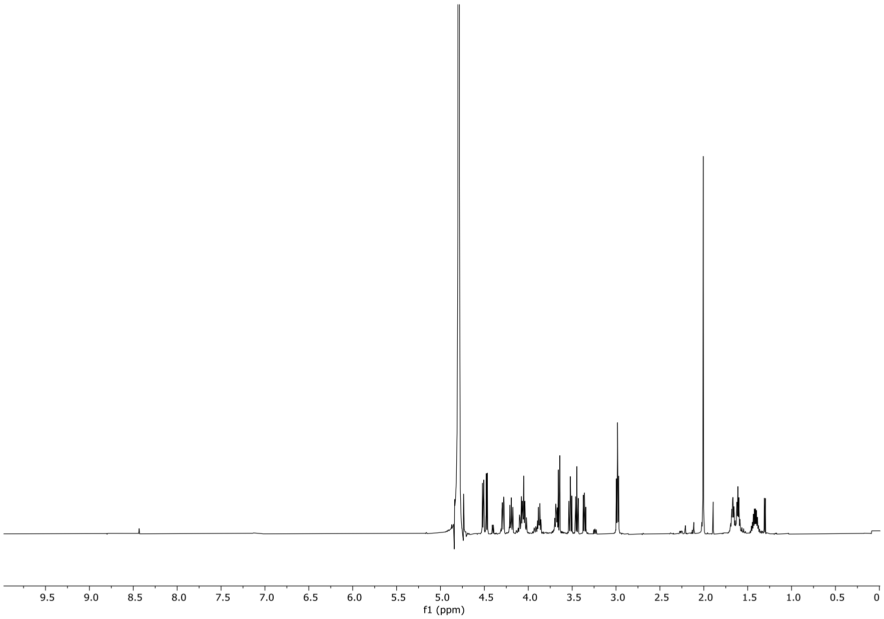

^{13}C NMR of 20 (151 MHz, D_2O)

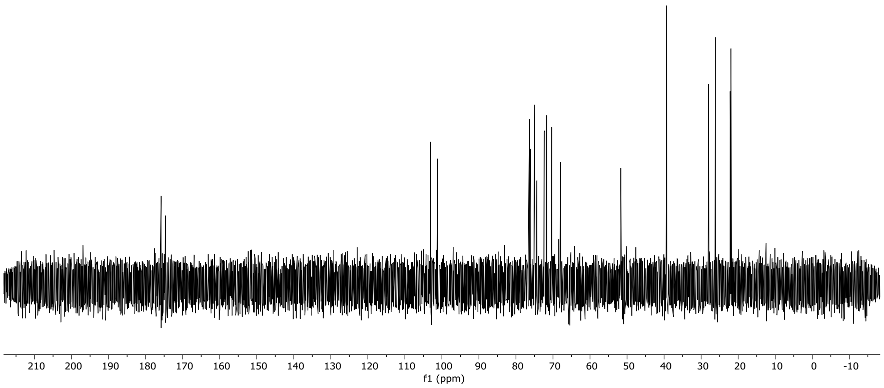

HSQC NMR of 20 (D_2O)

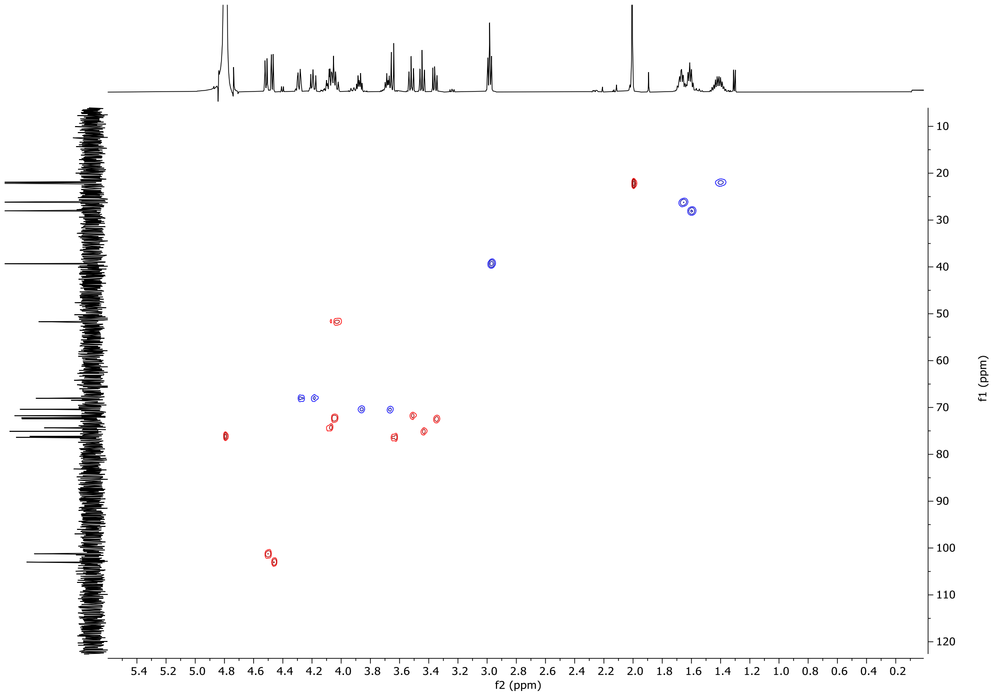

RP-HPLC of 20 (ELSD trace, Method C1, t_R= 11.76 min)

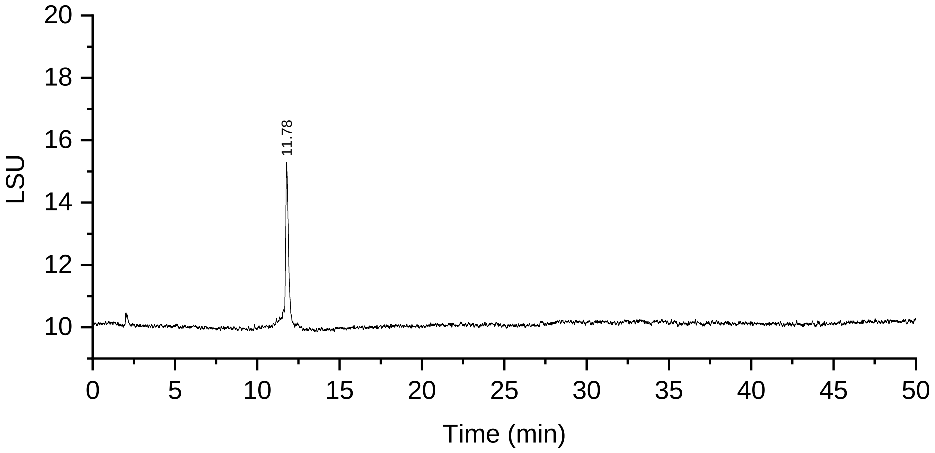

11 Mass spectrometry and additional information

Representative examples of reaction monitoring are reported. MALDI and/or ESI-MS is performed after microcleavage from the solid support at each step of the synthetic process.

11.1 Mass spectrometry analysis of the intermediate steps for the synthesis of 5

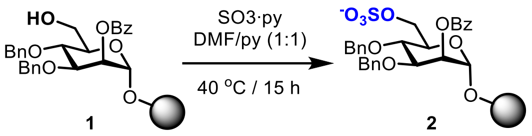

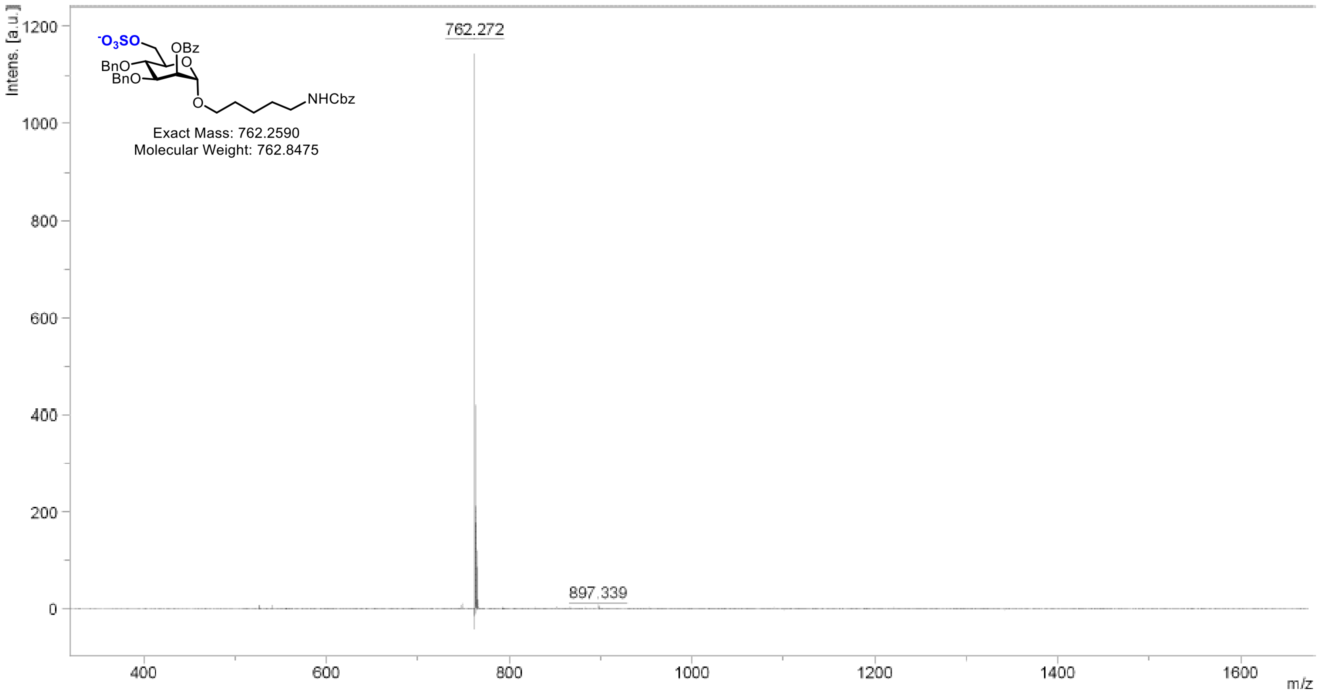

Figure S2. MALDI-TOF of compound **2** after microcleavage (negative mode).

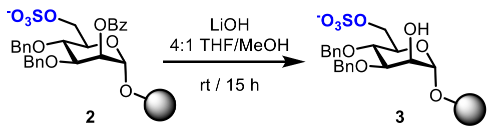

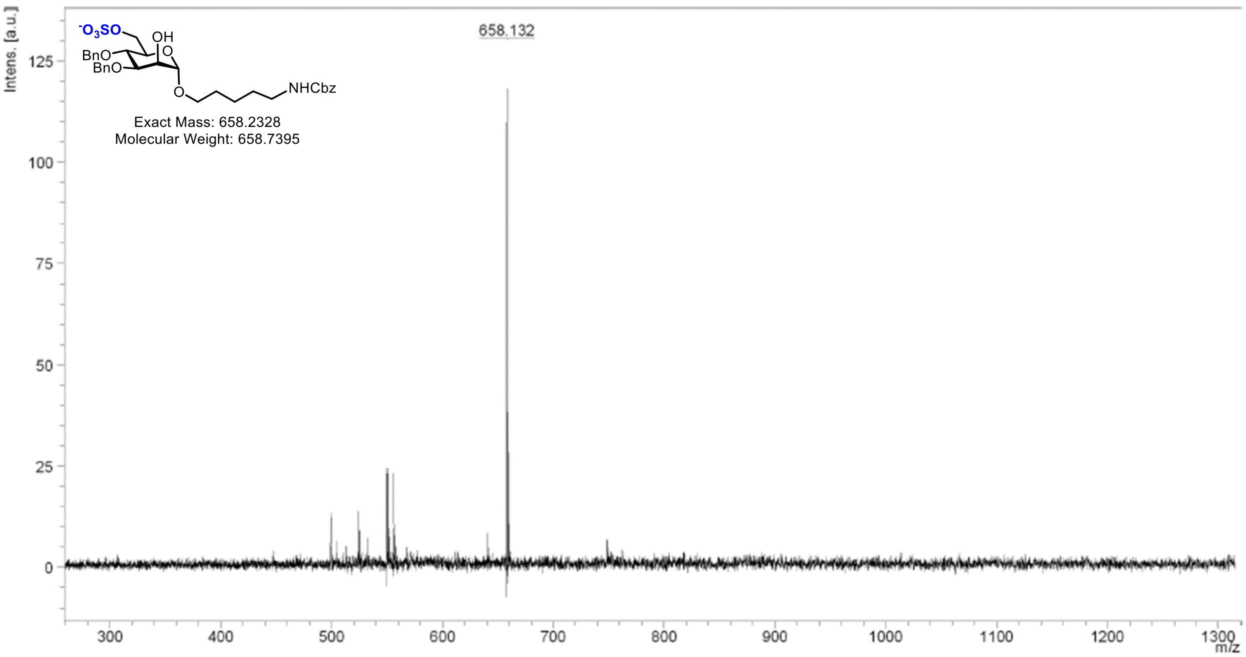

Figure S3. MALDI-TOF of compound **3** after microcleavage (negative mode).

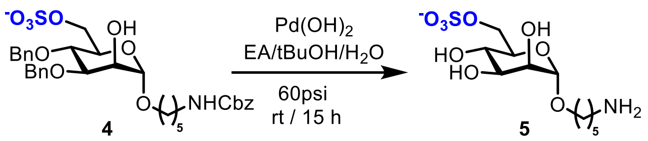

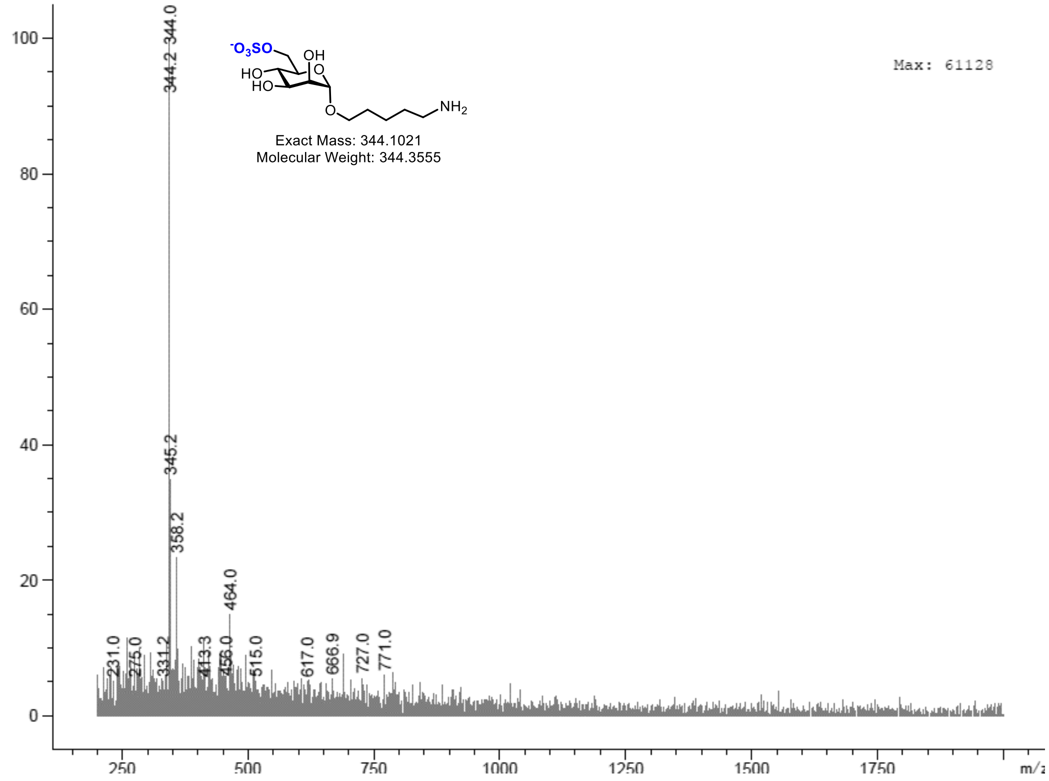

Figure S4.ESI-MS of crude compound **5** (negative mode).

11.2 Mass spectrometry analysis of the intermediate steps for the synthesis of 14

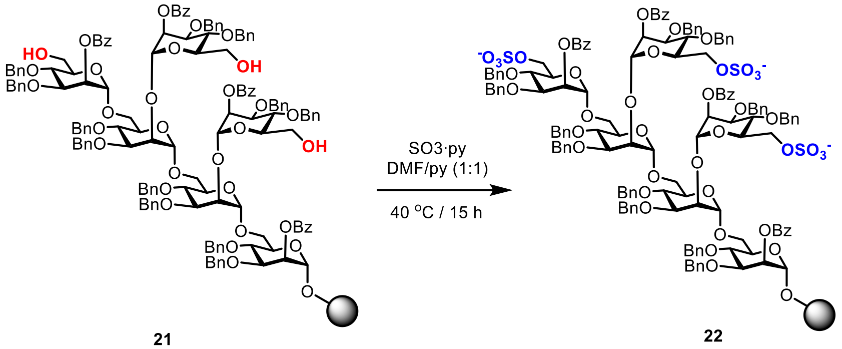

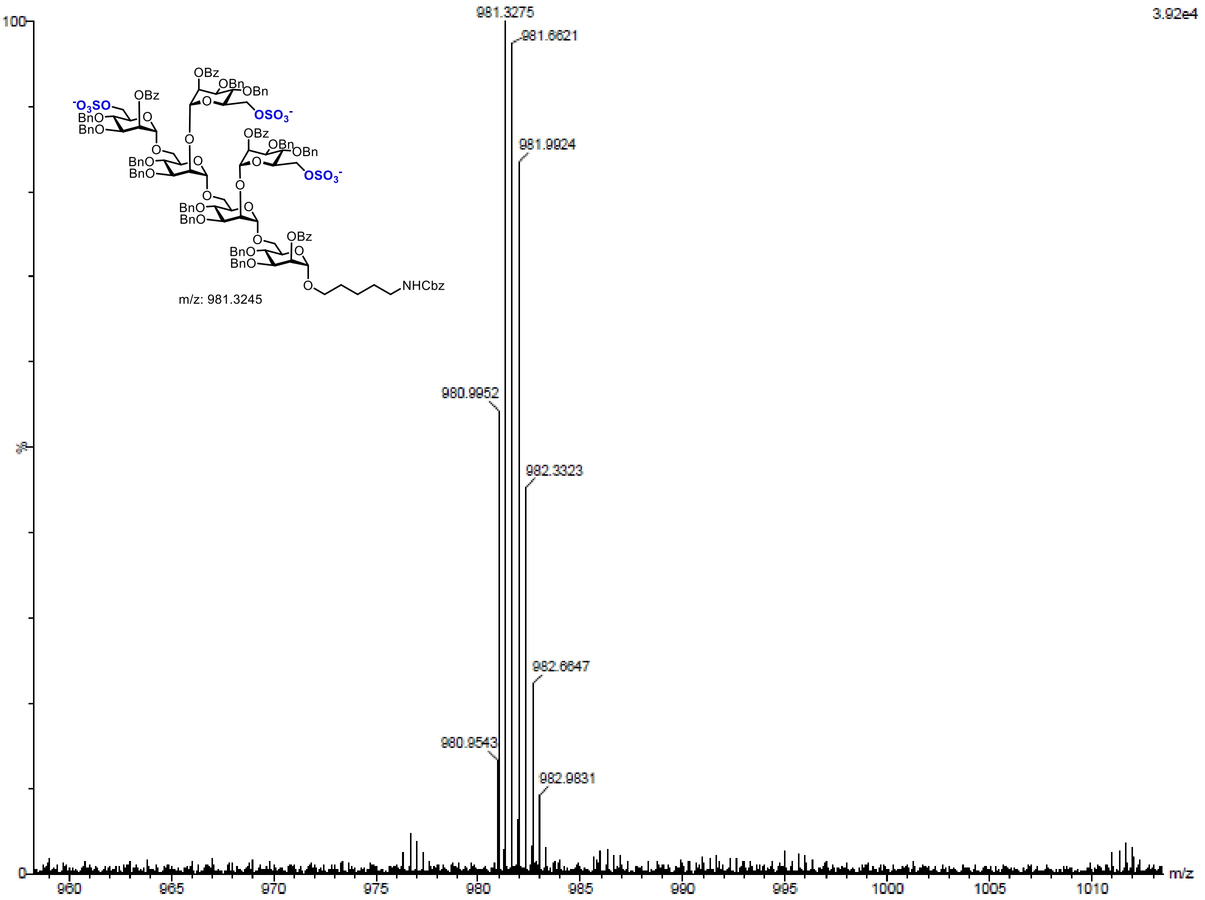

Figure S5. QTOF-MS of compound **22** after microcleavage (negative mode).

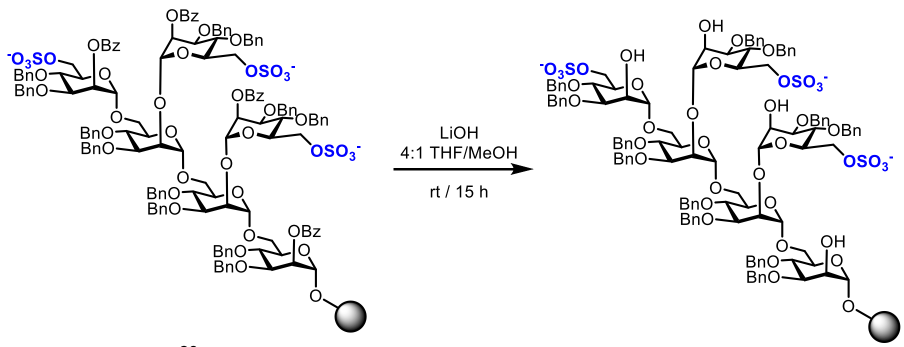

23 **24**

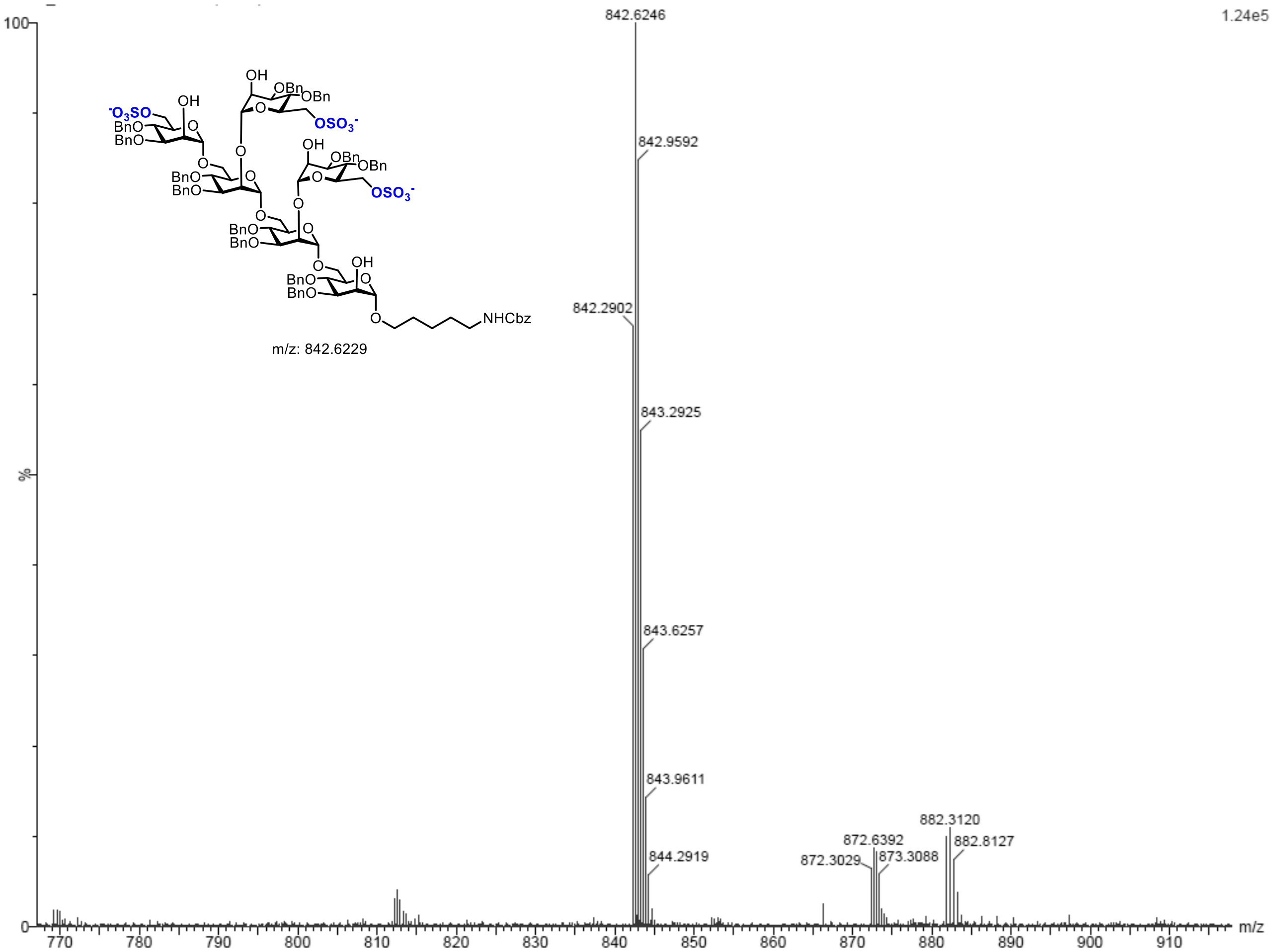

Figure S6. QTOF-MS of compound **24** after microcleavage (negative mode).

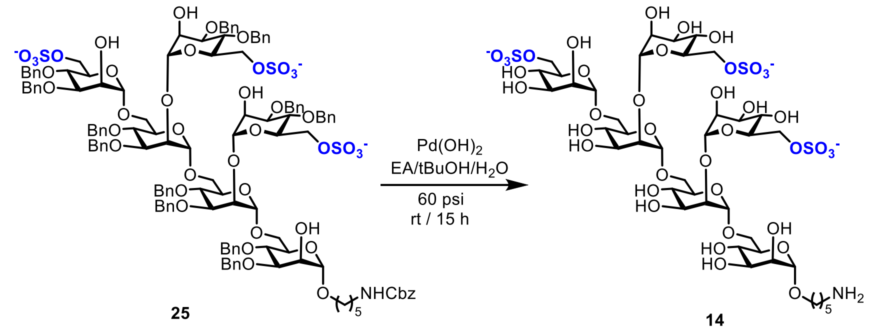

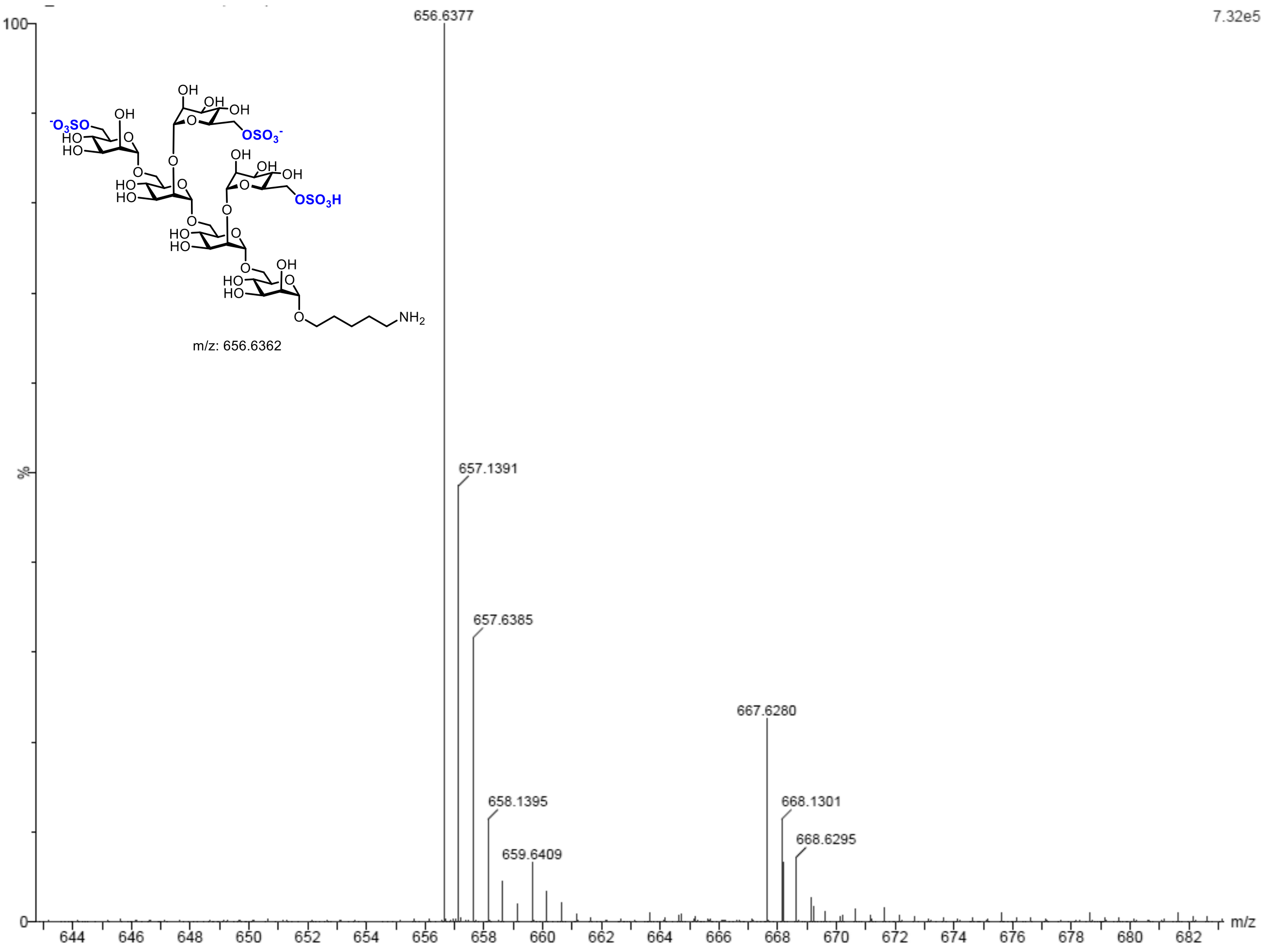

Figure S7. QTOF-MS of compound **24** after hydrogenolysis (negative mode).

11.3 Mass spectrometry analysis of the intermediate steps for the synthesis of 11

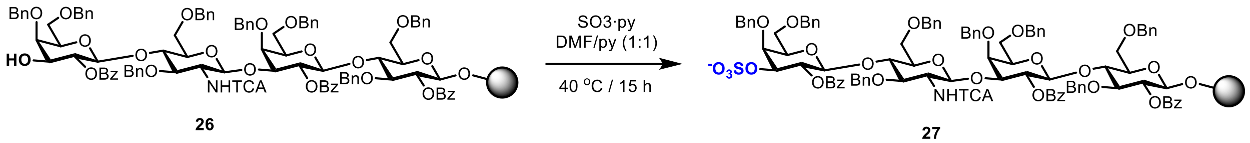

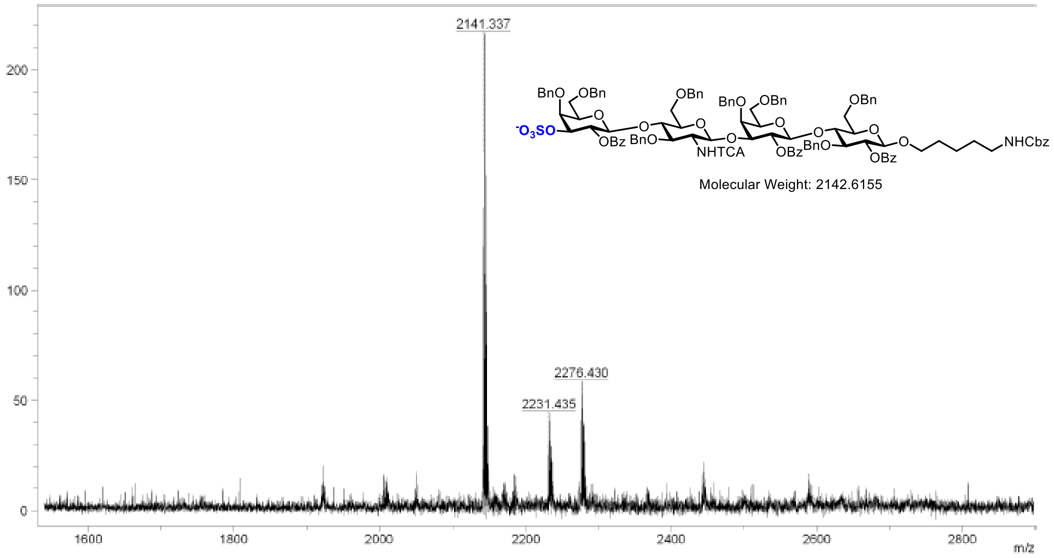

Figure S8. MALDI-TOF of compound **27** after microcleavage (negative mode).

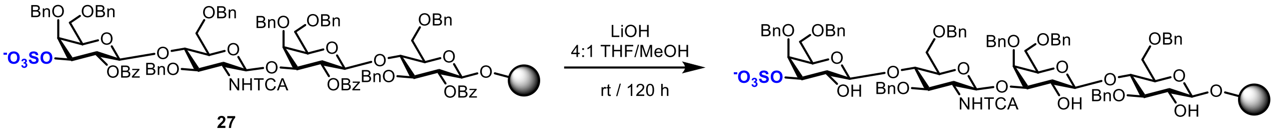

27

28

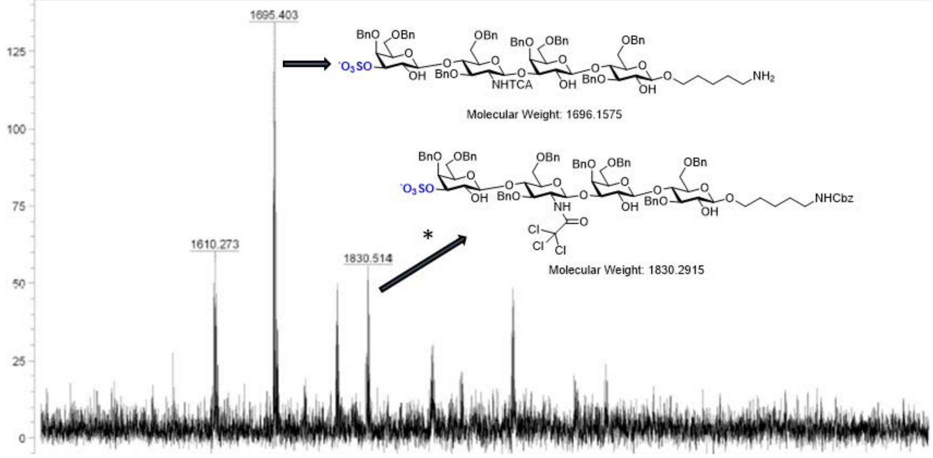

*Note: The loss of Cbz protecting group is commonly detected by MALDI and it is due to the long time exposed to light during microcleavage.

Figure S9. MALDI-TOF of compound **28** after microcleavage (negative mode).

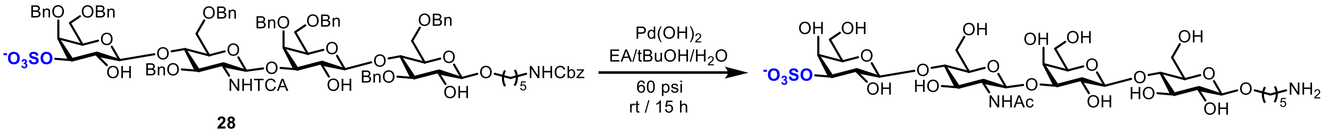

28

11

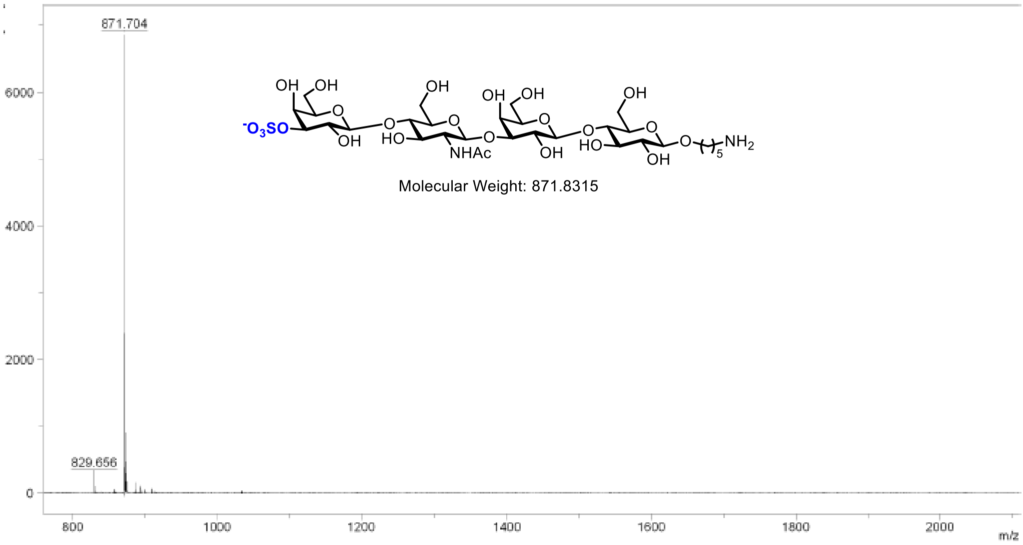

Figure S10. MALDI-TOF of compound **11** after hydrogenolysis (negative mode).

11.4 Mass spectrometry analysis of the intermediate steps for the synthesis of 24 and 27

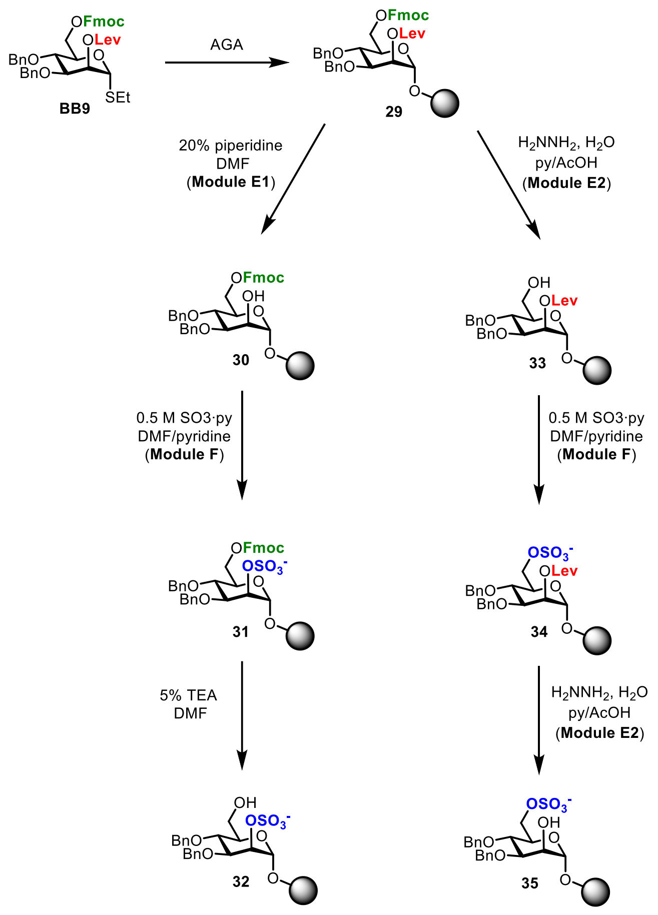

Figure S11. On resin selective removal of Fmoc and Lev PGs after sulfation.

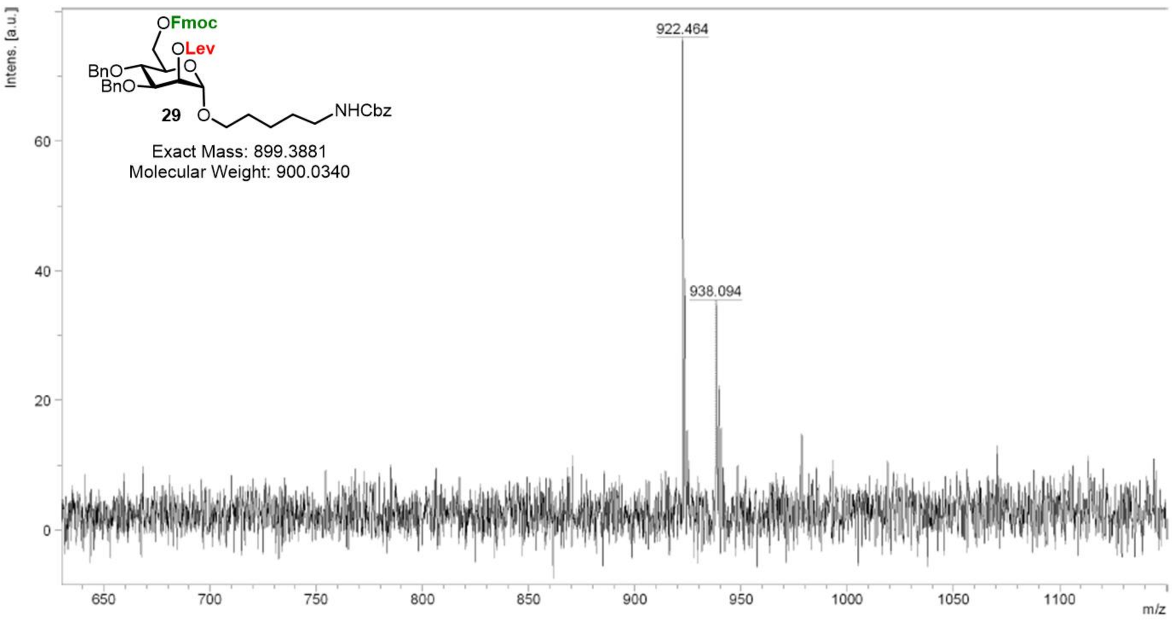

Figure S12. MALDI-TOF of compound **29** after microcleavage (positive mode).

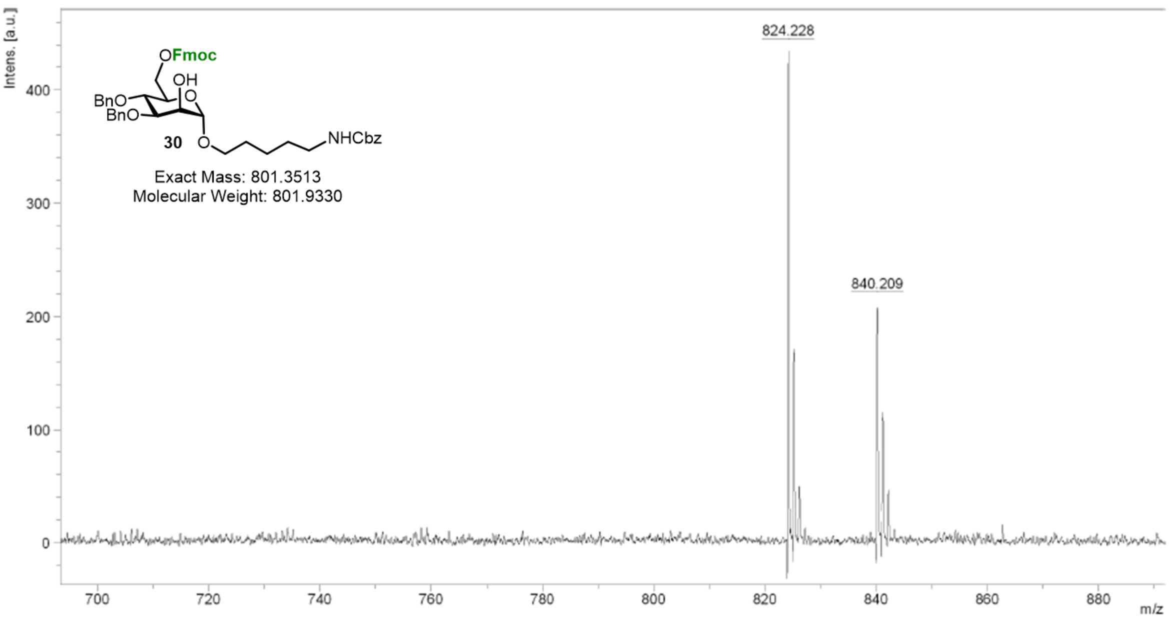

Figure S13. MALDI-TOF of compound **30** after microcleavage (positive mode).

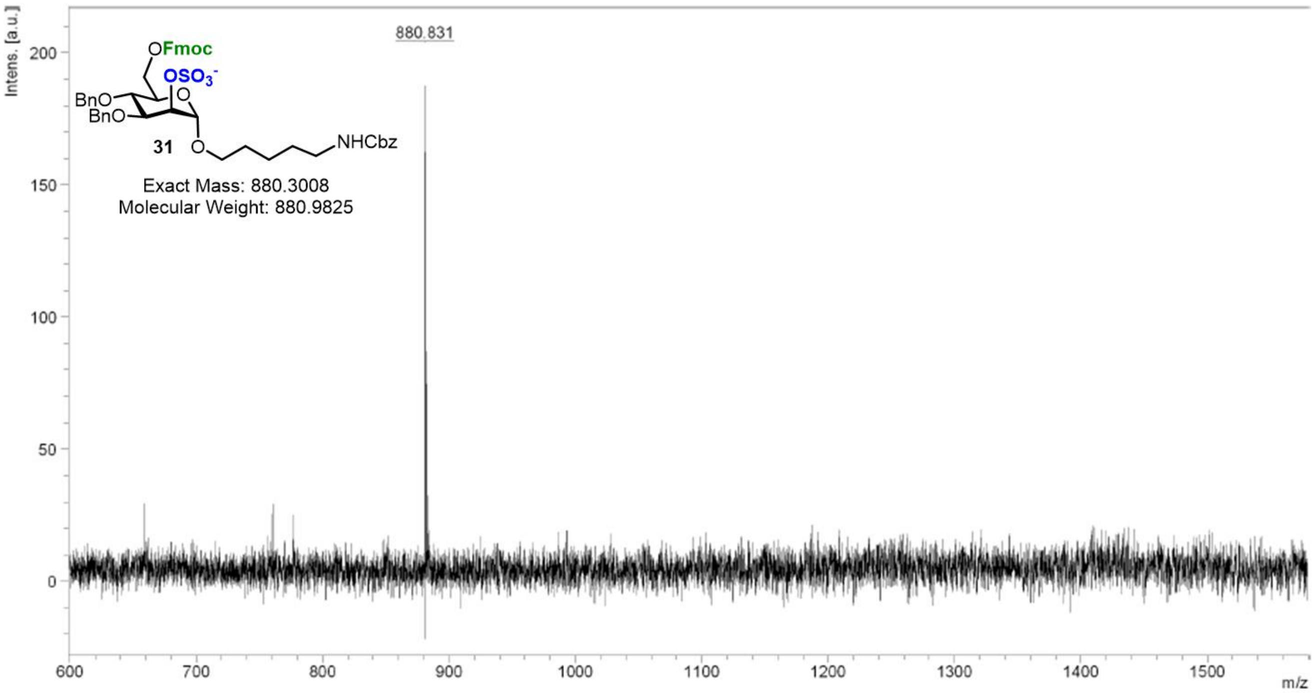

Figure S14. MALDI-TOF of compound **31** after microcleavage (negative mode).

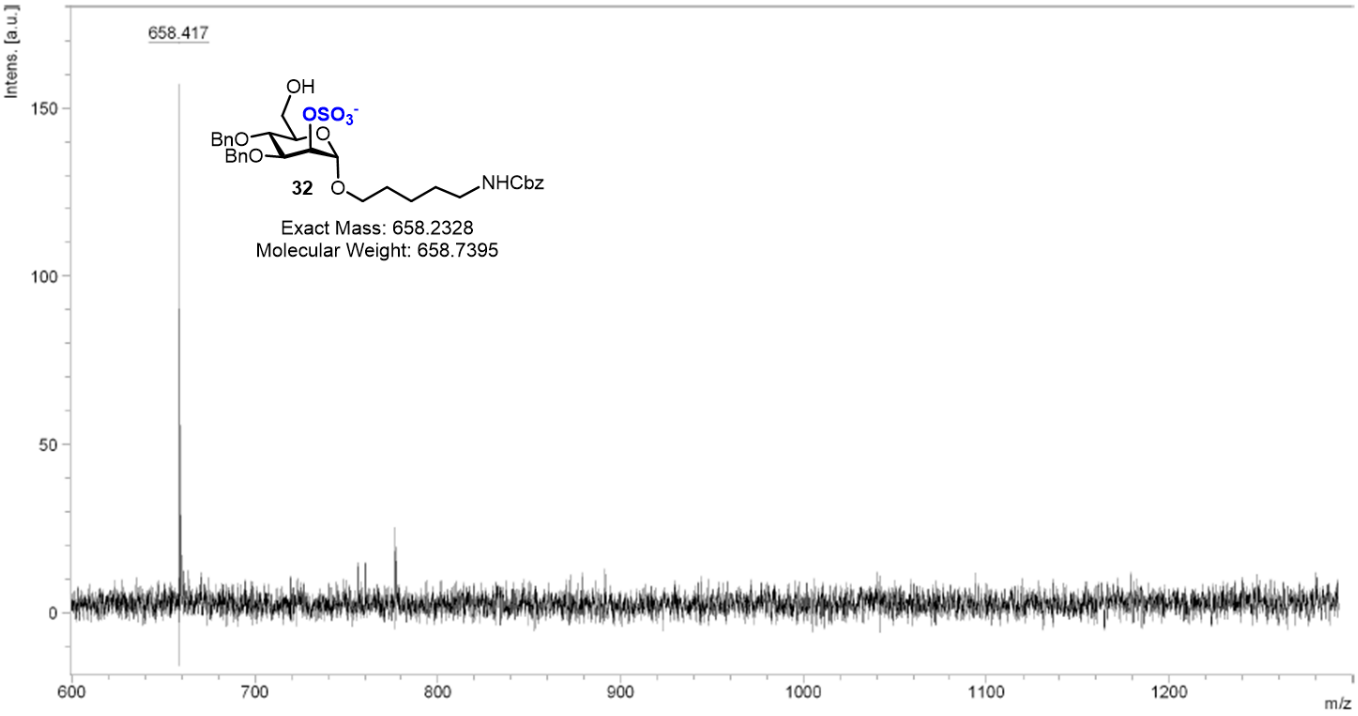

Figure S15. MALDI-TOF of compound **32** after microcleavage (negative mode).

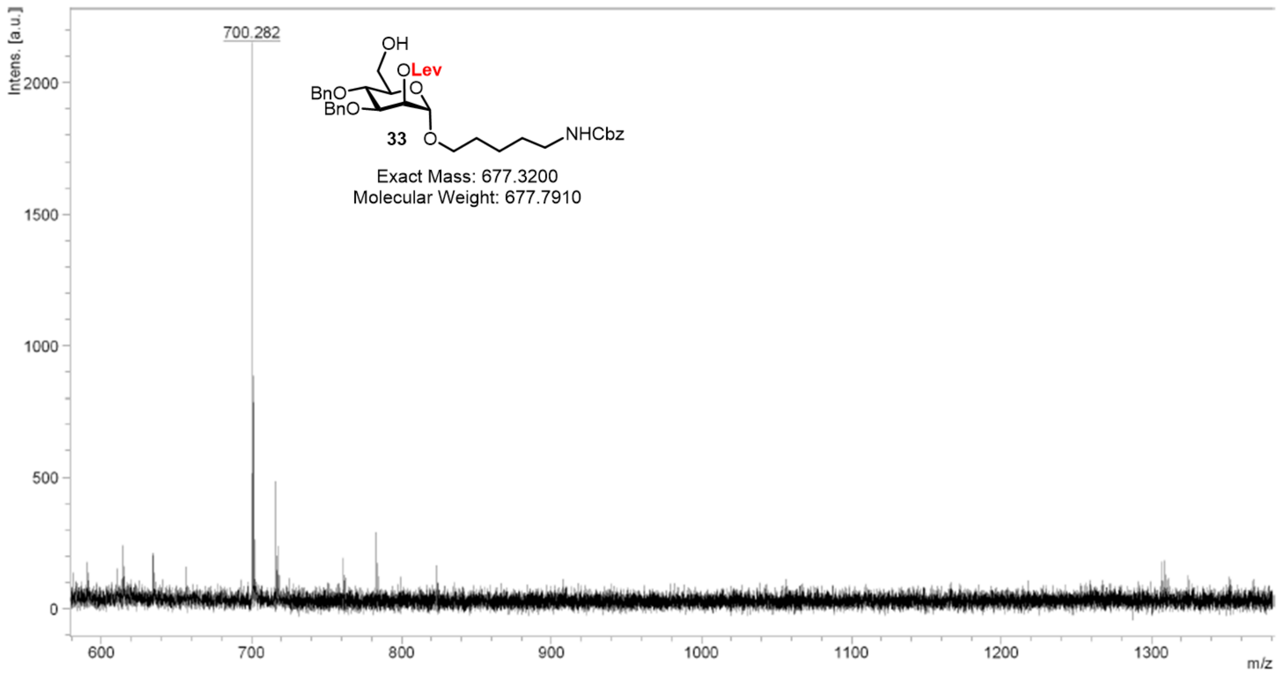

Figure S16. MALDI-TOF of compound **33** after microcleavage (positive mode).

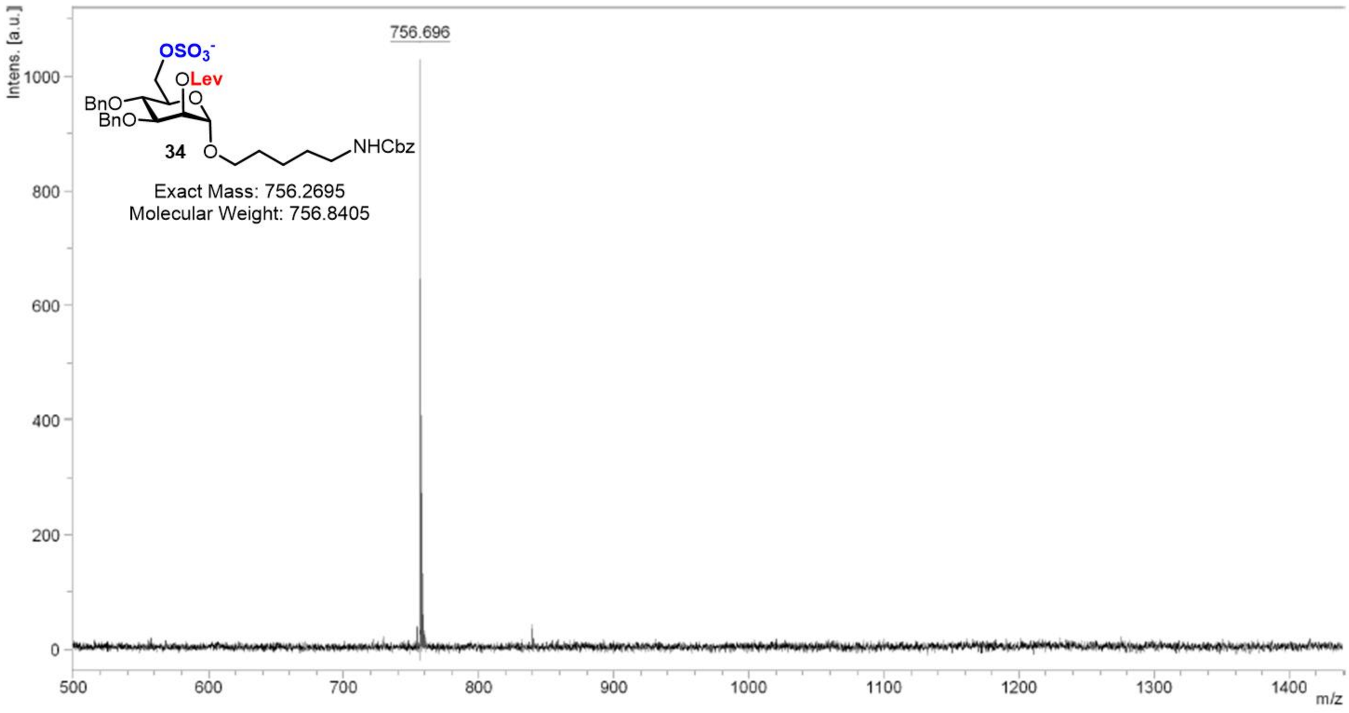

Figure S17. MALDI-TOF of compound **34** after microcleavage (negative mode).

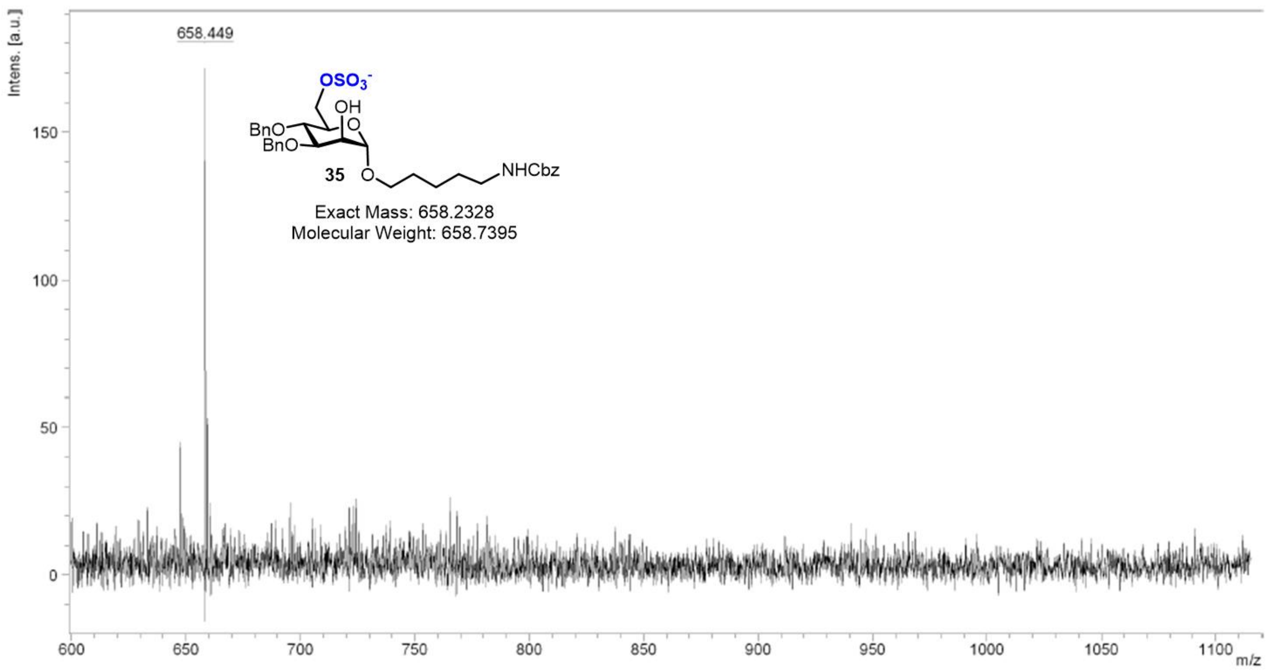

Figure S18. MALDI-TOF of compound **35** after microcleavage (negative mode).

11.5 Analysis of the hydrogenolysis reaction of compound 9a

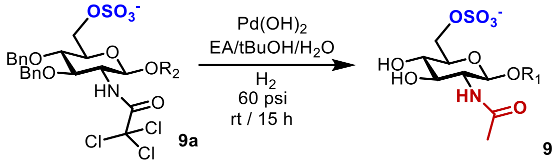

A)

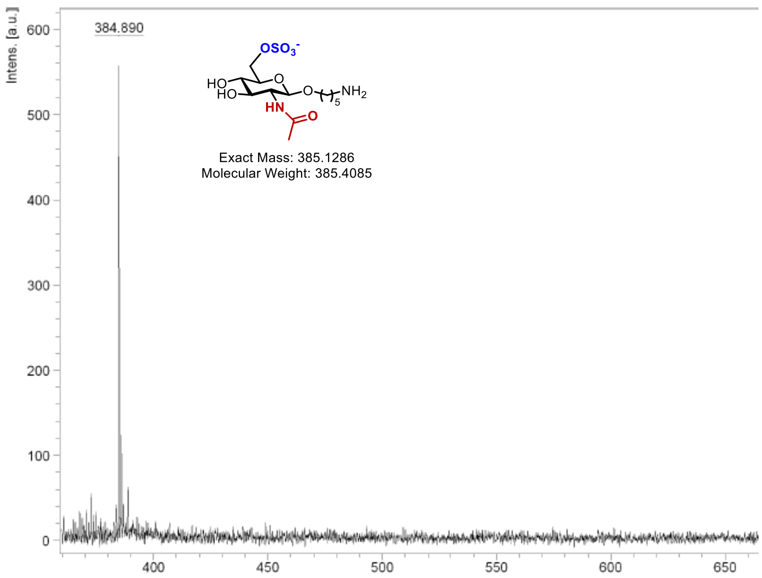

B)

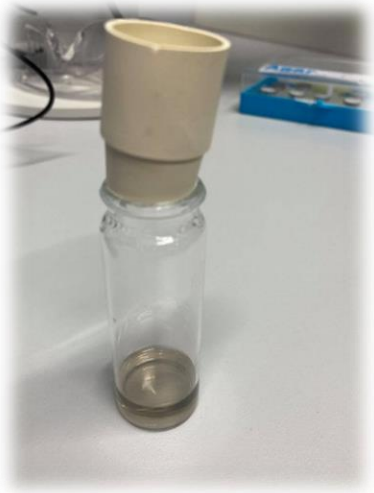

C)

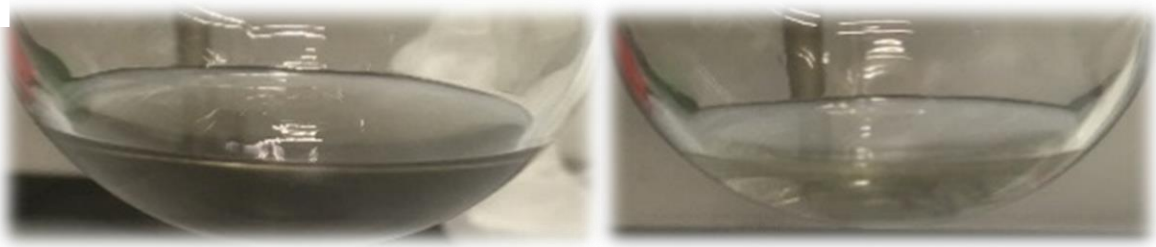

Figure S19. A) MALDI-TOF analysis of the crude reaction indicating completion of the hydrogenolysis step (negative mode). B) Upon completion of hydrogenolysis, filtration resulted in a black solution confirming the presence of residual Pd/C catalysts. C) Picture of the sample solutions obtained upon filtartion after hydogenolysis without (left) and with (right) thiourea treatment.

A)

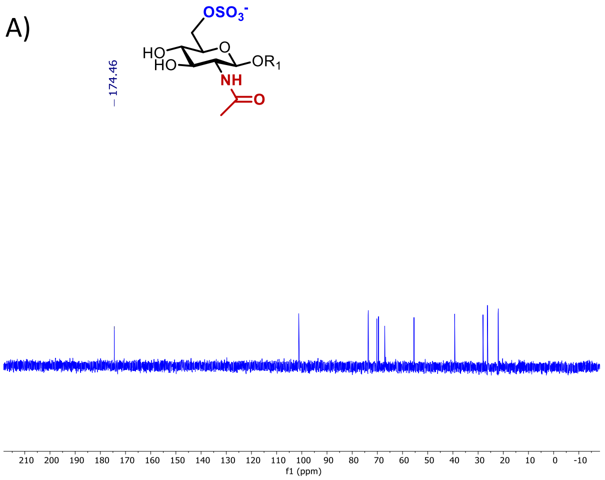

B)

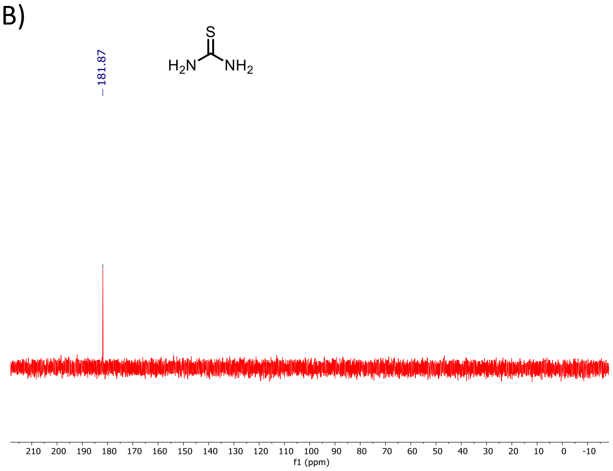

C)

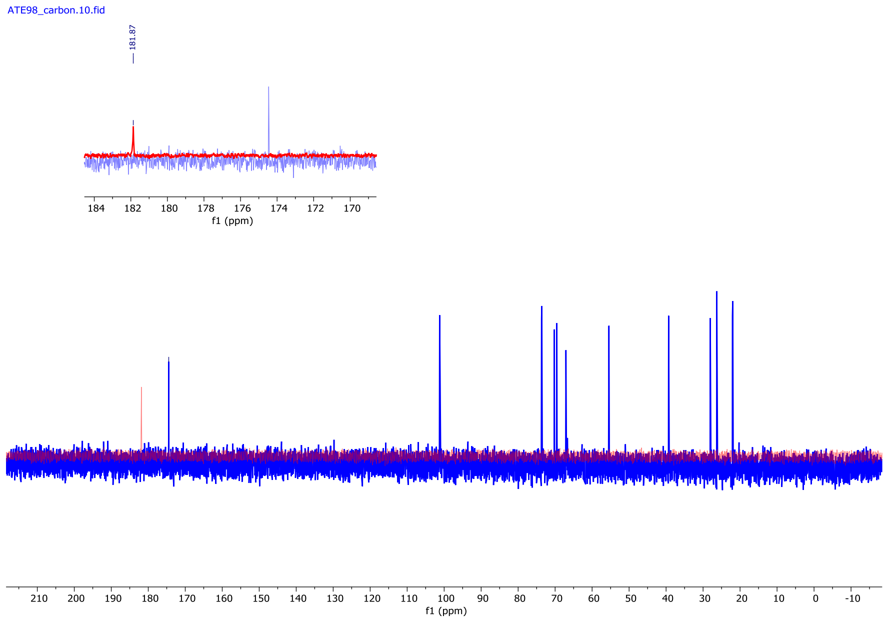

Figure S20. A) ^{13}C NMR of **9** (151 MHz, D_2O), B) ^{13}C NMR of thiourea (151 MHz, D_2O) , C) stacked spectra to confirm the complete removal of thiourea from the final compound.

12 References

1 K. Le Mai Hoang, A. Pardo-Vargas, Y. Zhu, Y. Yu, M. Loria, M. Delbianco and P. H. Seeberger, *J. Am. Chem. Soc.*, 2019, **141**, 9079–9086.

2 K. Daragics and P. Fügedi, *Org. Lett.*, 2010, **12**, 2076–2079.

3 K. Daragics and P. Fügedi, *Tetrahedron*, 2010, **66**, 8036–8046.

4 P. Chassagne, L. Raibaut, C. Guerreiro and L. A. Mulard, *Tetrahedron*, 2013, **69**, 10337–10350.
